# Sirtuin 6 mediates the therapeutic effect of endometrial regenerative cell-derived exosomes in alleviation of acute transplant rejection by weakening c-myc-dependent glutaminolysis

**DOI:** 10.3389/fcell.2025.1564382

**Published:** 2025-09-18

**Authors:** Tong Liu, Chenglu Sun, Xu Liu, Pengyu Zhao, Bo Shao, Yini Xu, Yiyi Xiao, Hongda Wang, Qiang Chen, Guangmei Yang, Hao Wang

**Affiliations:** ^1^ Department of General Surgery, Tianjin Medical University General Hospital, Tianjin, China; ^2^ Tianjin General Surgery Institute, Tianjin, China; ^3^ Tianjin Key Laboratory of Precise Vascular Reconstruction and Organ Function Repair, Tianjin, China

**Keywords:** endometrial regenerative cells, exosomes, SIRT6, CD4 + T cell differentiation, glutaminolysis, acute rejection

## Abstract

**Background:**

Despite the rapid development of immunosuppressive drugs, acute rejection (AR) remains a cause of allograft dysfunction and allograft failure. Although endometrial regenerative cell-derived exosomes (ERC-Exos) effectively alleviate AR, more research is required to fully understand the underlying mechanisms. Thus, this study aimed to determine whether sirtuin 6 (SIRT6) mediates the therapeutic effect of ERC-Exos on AR and elucidate the underlying mechanisms.

**Methods:**

The expression of SIRT6 was verified in ERC-Exos by Western blot. ERC-Exos with extremely low expression of SIRT6 (SIRT6-KD-ERC-Exos) were obtained by transducing shRNA-SIRT6 in ERCs. C57BL/6 recipient mice were transplanted with heart grafts from BALB/c donor mice and divided into three groups: untreated, ERC-Exo-treated, and SIRT6-KD-ERC-Exo-treated groups. Recipient mice were sacrificed on post-operative day 8 for the determination of graft pathological changes, intra-graft immunocyte infiltration, splenic CD4^+^ T cell populations, and serum cytokine levels *in vivo*. The proportion of CD4^+^ T cells and their secreting cytokine levels were determined *in vitro*. Besides, the underlying mechanisms were also investigated *in vitro*.

**Results:**

ERC-Exos expressed SIRT6, and cardiac graft survival was increased by SIRT6-expressing ERC-Exos. Graft pathological damage, intra-graft CD4^+^ T cell infiltration, and intra-graft inflammatory (Th1 and Th17) cell infiltration decreased, and intra-graft and serum inflammatory cytokine (interferon (IFN)-γ and interleukin (IL)-17) levels decreased in the SIRT6-expressing ERC-Exo-treated mice. Furthermore, in the recipient mice, ERC-Exo treatment markedly increased the differentiation of regulatory T cells (Tregs) while significantly decreasing that of Th1 and Th17 cells. In a similar vein, ERC-Exo therapy raised the levels of the anti-inflammatory cytokine IL-10 *in vitro* while decreasing those of IFN-γ and IL-17. By suppressing the expression of important proteins linked to glutaminolysis and further deactivating the mammalian target of rapamycin complex 1 (mTORC1) pathway, ERC-Exos reduced the uptake and use of glutamine in naïve CD4^+^ T cells, according to mechanism exploration. In contrast, SIRT6-KD-ERC-Exos considerably reversed these trends and changes both *in vivo* and *in vitro*.

**Conclusion:**

SIRT6 is crucial in mediating ERC-Exos to remodel CD4^+^ T cell differentiation by weakening c-Myc-dependent glutaminolysis, thereby alleviating AR.

## Introduction

Despite the availability of alternative treatments, organ transplantation remains the most reliable means to treat end-stage organ failure (Shah et al., 2022; Kittleson and Kobashigawa, 2017). Acute rejection (AR) usually occurs from a few days to 2 weeks after transplantation, which is the most common rejection in organ transplantation and affects transplanted organs’ early survival (Stewart et al., 2007). AR is mainly caused by the HLA complex incompatibility between donor and recipient, and more involved in T cells (Stewart et al., 2007). Besides, long-term use of immunosuppressive drugs will lead to many negative effects, including tumorigenesis and lethal infections (Söderlund and Rådegran, 2015). Hence, safer and more effective immunotherapeutic methods should be explored to alleviate AR.

Due to their extremely low expression of major histocompatibility complex (MHC) class II and absence of MHC class I as well as co-stimulatory molecules, mesenchymal stem cells (MSCs) are classified as “immune privileged cells” (Quaranta et al., 2016). MSCs are immune-privileged, so they cannot induce rejection and can perform their original functions, such as tissue repair and immune regulation. As a result, MSC injection is thought to be a viable therapy option for a variety of disorders. For instance, the preliminary findings of a multicenter randomized controlled trial revealed that injecting umbilical cord-derived MSCs following kidney transplantation is safe and effective in reducing AR incidence (Sun et al., 2018). However, the invasive harvesting procedure, restricted availability, and potential risk of allogeneic use limit the method’s widespread clinical adoption.

Endometrial regenerative cells (ERCs) are a new type of MSCs collected from menstrual blood with the advantages of non-invasive acquisition and autologous stem cell reinfusion therapy (Darzi et al., 2016). We previously reported that ERC therapy can ameliorate a variety of immune-related disorders *in vivo*, including transplant rejection, ulcerative colitis, and autoimmune hepatitis, via modulating CD4^+^ T cells (Hu et al., 2021; Li et al., 2019; Wang et al., 2021). In heart transplantation model mice, ERC therapy can alleviate transplantation rejection by decreasing the helper T cell 1 (Th1) and 17 (Th17) proportion, while increasing the percentage of regulatory T cells (Tregs) (Hu et al., 2021). Although ERC treatment shows promise, its clinical uses have some limits. As a kind of MSC, ERCs are inevitably associated with certain common problems of cell therapy, such as diminished therapeutic efficacy caused by pulmonary network blockage or cell embolism from repeated injections (Klabukov et al., 2023). Therefore, cell-free therapeutic strategies are being developed. Similarly, our prior research found that ERC-derived exosomes (ERC-Exos) show a significant therapeutic effect on AR, which could be attributed to their regulatory function in CD4^+^ T cell differentiation.

Sirtuin 6 (SIRT6), an important member of the NAD^+^-dependent histone deacetylase SIRT family, is a multifunctional protein involved in various enzymatic activities, including histone deacetylation, de-fatty acylation, and mono-ADP-ribosylation (Li et al., 2022). It was initially identified as an anti-aging protein, but more investigations have revealed numerous activities of SIRT6, such as regulating the inflammatory response, resisting oxidative stress, and maintaining metabolism (Li et al., 2022; Guo et al., 2022). For example, SIRT6 inhibits IL-6 secretion in macrophages by weakening the NF-κB pathway and downregulating STAT3 mRNA transcription, thus preventing macrophages from polarizing towards a pro-inflammatory phenotype (Lee et al., 2017). Aside from its regulatory function on macrophage polarization, considering that NF-κB pathway activation is important in T cell activation (Schmitz et al., 2003), SIRT6 may have some regulatory effects on T cells. A follow-up study showed that in rheumatoid arthritis, SIRT6 inhibition reduces the percentage of Tregs in the peripheral blood and synovial fluid (Wang et al., 2019), indicating that SIRT6 has the potential to regulate CD4^+^ T cell differentiation. Studies have also shown that SIRT6 can negatively regulate c-Myc, a key target expressed in large quantities during naïve CD4^+^ T cell activation, through histone deacetylation (mainly histone H3 lysine K9 and histone H3 lysine K56) (Sebastián et al., 2012). Specifically, the upregulation of c-Myc during naïve CD4^+^ T cell activation can promote the transcription of downstream glutaminolysis-related genes, such as glutaminase 1 (*GLS1*) and solute carrier family 1 member 5 (*SCL1A5*), an encoding gene of alanine-serine-cysteine transporter system 2 (ASCT2) protein (Wang et al., 2011). This process is also known as c-Myc-dependent glutaminolysis (Marchingo et al., 2020). Thus, SIRT6 shows great potential in the regulation of glutaminolysis in naïve CD4^+^ T cells.

Glutamine, an immune regulatory nutrient, is frequently used in large amounts for cellular energy and facilitates the intracellular synthesis of genetic material via glutaminolysis in rapidly proliferating and dividing cells (Yang et al., 2017). For naïve CD4^+^ T cells to proliferate and differentiate, they need to rapidly take up a large amount of glutamine right after activation and subsequently initiate glutaminolysis (Geltink et al., 2018). Naïve CD4^+^ T cells can differentiate into different subtypes following antigen stimulation via different metabolic patterns (Liu et al., 2023). Specifically, the differentiation of Th1 and Th17 cells tends to involve glutaminolysis, whereas that of Tregs does not (Geltink et al., 2018). Even under specific cell differentiation conditions, impaired glutaminolysis can substantially affect the fate of peripheral naïve CD4^+^ T cells during differentiation (Yang et al., 2021). For example, even under Th1 polarization, naïve CD4^+^ T cells with glutamine deficiency also tilt toward Foxp3^+^ Tregs and hinder their differentiation into Th1 cells (Klysz et al., 2015). AR is accompanied by disordered CD4^+^ T cell differentiation, especially increased Th1 and Th17 cell proportions and decreased Treg proportions. Therefore, remodeling disordered CD4^+^ T cell differentiation by modulating glutaminolysis appears to be a viable way to alleviate AR.

Early research found that SIRT6 is widely expressed in various cells and is mainly localized in the nucleus (Pillai and Gupta, 2020). However, subsequent research showed that SIRT6 is also expressed and accumulated in cytoplasm (Hou et al., 2022). Thus, research on SIRT6 gradually shifted from intracellular to extracellular investigations. A recent study has reported that SIRT6 can express in the bone marrow-derived MSC exosomes and exert its deacetylation to inhibit phosphate-induced aortic calcification (Wei WQ. et al., 2021). Given SIRT6’s encouraging immunomodulatory potential, we looked into how it might mediate the therapeutic effects of ERC-Exos. As mentioned previously, SIRT6 shows a promising ability to remodel disordered CD4^+^ T cell differentiation by regulating c-Myc-dependent glutaminolysis. Therefore, this study aimed to explore whether ERC-Exos expresses SIRT6 and whether the ERC-Exo therapy effect in alleviating allogeneic immune response is mediated by SIRT6. We also investigated whether SIRT6-expressing ERC-Exos can improve aberrant CD4^+^ T cell differentiation on AR by weakening glutaminolysis.

## Materials and methods

### Isolation, culture, and detection of ERCs

All human ERCs used in this study were isolated from menstrual blood provided by healthy female volunteers aged 20–30 years, and the procedure was approved by Tianjin Medical University General Hospital (IRB2024-YX-013-01, Tianjin, China). First, menstrual blood is filtered to remove blood clots, and the bottom red precipitate is obtained by centrifugation. Then, these red precipitates were resuspended by PBS, slowly added to the same volume of human peripheral blood lymphocyte separation solution, and ERCs were obtained by density gradient centrifugation. Finally, the extracted ERCs were cultured in Dulbecco’s modified Eagle’s medium/Ham’s F12 (DMEM/F12) nutrient medium supplemented with 10% fetal bovine serum (FBS) and 1% P/S solution in a 37 °C 5% CO_2_ incubator. After being incubated for approximately 1–2 ° weeks, ERCs were harvested and phenotypically identified using flow cytometry. The identified ERCs were then used for further studies.

### Three-lineage experiment

In order to further identify the stem cell characteristics of ERCs, the three-line differentiation experiments were carried out. 1. Osteogenic differentiation: 0.1% gelatin was added into a 12-well plate, with 500 μL in each hole, so that it covered the whole bottom surface. Then the gelatin coated 12-well plate was placed in a 37 °C incubator for at least 30 min. After 30 min, the gelatin was discarded and used to inoculate cells after the Petri dish was dried. ERCs were inoculated into a 12-well plate coated with gelatin in the amount of 4 × 10^4^ cells per well, and cultured in serum-free medium, and the medium was changed once a day. When the confluence degree of ERCs reached 70%, the medium was discarded by suction, and 1 mL of human mesenchymal stem cells osteogenic differentiation medium was added to each well to start the induced differentiation. After 21–30 days of ERC osteogenesis induction, the precipitation of calcium nodules was observed under the microscope, and the osteogenic differentiation was identified by alizarin red S staining. 2. Chondrogenic differentiation: 3 × 10^5^ ERCs were transferred to a 15 mL centrifuge tube, and the centrifuge was 300 *g* for 5 min. After centrifugation, the supernatant was poured out, 500 μL of human mesenchymal stem cells were added into chondrogenic differentiation medium, and the cells were resuspended. Centrifuge: 300 *g* for 5 min. The centrifuge tube cover was unscrewed for gas exchange, and it was kept in a cell incubator for 24 h. After 24–48 °h, when the cells aggregated, the bottom of the centrifuge tube was flicked to make the cartilage ball separate from the bottom of the tube and suspend in the liquid. The cell culture medium was changed every 2–3° days to prevent the cartilage ball from being scattered. After 21–28° days of chondrogenic differentiation of ERCs, the differentiation medium was discarded, and the chondrocytes were washed twice with 3 mL PBS, and then fixed with 3 mL 4% paraformaldehyde. The fixed chondrocytes were embedded in paraffin, and the chondrogenic differentiation was identified by alixin blue staining. 3. Adipogenic differentiation: ERCs were inoculated into 12-well plates with the number of 4 × 10^4^ cells per well, and cultured in serum-free medium. When the confluence of ERCs reached 100%, the medium was discarded, and 1 mL of adipogenic differentiation medium was added to each well to start differentiation induction. Adipogenic differentiation was induced for 9–35° days. After the formation of lipid droplets was observed under the microscope, the adipogenic differentiation was identified by oil red O staining.

### Construction of ERCs with low expression of SIRT6 (SIRT6-KD-RECs)

To knock down SIRT6 in ERCs and further obtain ERC-Exos with low expression of SIRT6 (SIRT6-KD-ERC-Exos), We bought shRNA-SIRT6 lentivirus containing green fluorescent protein (GFP) and puromycin resistance gene from Gene-Chem company (Shanghai, China). The sequence of shRNA-SIRT6 was as follows: 5′-CGAGGATGTCGGTGAATTATTCAAGAGATAATTCACCGACATCCTCG-3’. Specifically, the SIRT6-Knockdown virus consisted of Lv-hU6-MCS (shRNA-SIRT6 sequence insertion site) -CBh-gcGFP-IRES-puromycin. Similarly, the empty vector virus had the same architecture. Empty vector virus inserted an unintentional sequence (5′-TTCTCCGAACGTGTCACGT-3′) at MSC site, which would not affect the expression of SIRT6. Lentiviral transduction was carried out following the instructions from Gene-Chem, using an optimal multiplicity of infection (MOI = 20). ERCs at passage 2 were plated onto a 6-well plate with a density of 15,000/well, and lentiviral transduction was carried out when the confluence reached 50%–60%. The transduction time was 12–16 °h, and the transduction is terminated according to the cell condition. After transduction, the cells were washed by PBS and replaced with fresh medium without lentivirus. To select for successfully transduced cells, puromycin (2 μg/mL, Solarbio, China) was incorporated into the culture medium. At the same time, puromycin was added to the ERCs of un-transduced lentivirus with the same generation and density, and the screening was finished when it died completely. After the cell screening, the GFP positive cells were detected by fluorescence microscope to be more than 90%, and then Western blot was carried out to detect SIRT6 expression level in ERCs. After successfully constructing SIRT6-KD-ERCs, the supernatant was collected to isolate SIRT6-KD-ERC-Exos. Besides, SIRT6-NC-ERCs could be obtained by successfully transducing ERCs with empty vector virus. Similarly, the further obtained exosome was SIRT6-NC-ERC-Exos.

### Isolation and characterization of ERC-derived exosomes

ERCs were cultured in 10-cm dishes, washed with phosphate buffered saline (PBS), and maintained in FBS-free medium (Transgen, Beijing, China) for 48 h upon reaching nearly 90%–95% confluence. Then, the exosomes were obtained by centrifugation (Beckman centrifuge, United States) under the following conditions: 3,000 *g*, 4 °C, 10 °min; 10,000 *g*, 4 °C, 30 °min; 130,000 *g*, 4 °C, 70 min. The exosomes were resuspended with PBS and stored at −80 °C. The morphology of ERC-Exos was observed using transmission electron microscopy (TEM) (HT7700-SS, HITACHI, Japan). The size distribution of ERC-Exos was measured using nanoparticle tracking analysis (NTA) (Malvern Instruments Ltd., Malvern, United Kingdom). The concentration of ERC-Exos was determined using a bicinchoninic acid (BCA) protein assay kit (Solarbio, Beijing, China). In addition, exosomal markers, including CD9, CD63, TSG101, and calnexin, were detected using Western blot assay with the corresponding antibodies (dilution at 1:2000, 1:2,500, 1:2,000, and 1:3,000 respectively, abcam).

### Animals

Adult C57BL/6 and BALB/c mice (male, 6–8 ° weeks old, weighing 23–26 g) were obtained from the China Food and Drug Inspection Institute (Beijing, China). All animals used in this study were housed in a conventional experimental environment with sufficient space, water, food, and appropriate temperature at the Tianjin General Surgery Institute (Tianjin, China). All animal procedures followed the animal use protocol approved by the Animal Care and Use Committee of Tianjin Medical University (Tianjin, China), and all the experiments were performed in accordance with the guidelines of the Chinese Council on Animal Care.

### Allogeneic cardiac transplantation and experimental groups

C57BL/6 mouse recipients were randomly assigned to three experimental groups (n = 5): untreated, ERC-Exo-treated, and SIRT6-KD-ERC-Exo-treated groups (exosomes were obtained through ERC transduction by shRNA-SIRT6 lentivirus). As described in our previous study (Hu et al., 2021), an intra-abdominal heterotopic cardiac transplantation model was adopted, in which the hearts of BALB/c mice were transplanted into the abdomen of C57BL/6 mouse recipients. Specifically, the donor aorta and the recipient abdominal aorta, and the donor pulmonary artery and the recipient inferior vena cava anastomosed end to side. After the operation, the recipient mice were fed separately under appropriate conditions. In ERC-Exo and SIRT6-KD-ERC-Exo treatment groups, the corresponding exosome treatment was given respectively by tail vein injection on the first, third and fifth day after operation. In detail, each mouse was injected with 200 μg exosomes at a time on the injection day. The heartbeats of the grafts were recorded daily by the same member of our research team who was blinded to the treatment details. According to the force of the graft heartbeat, the pulsation was divided into three degrees: A, beating strongly; B, noticeable decline in the intensity of pulsation; and C, complete arrest of the graft heartbeat. For the graft survival time (n = 5), the days until the graft heartbeat was completely arrested were recorded. Based on our previous experience (Hu et al., 2021), for the assessment of efficacy and immune microenvironment, the recipient mice were euthanized on post-operative day (POD) 8, and the grafts and spleens were extracted for subsequent analyses.

### Histology assessment

After being formalin-fixed, dehydrated, and paraffin-embedded, the heart graft samples were sliced into sections with 5-μm thickness. The sections were stained with hematoxylin and eosin (H&E). All the finished products were used to evaluate the severity of rejection under a light microscope. The presence of myocyte necrosis, interstitial hemorrhage, lymphocyte infiltration, vasculitis, and intravascular thrombosis was detected and scored in accordance with previously described criteria (Wang et al., 2003). On comparison with normal tissues, the following scores can be obtained: 0, no rejection; 1, mild interstitial or perivascular infiltrate without necrosis; 2, focal interstitial or perivascular infiltrate with necrosis; 3, multifocal interstitial or perivascular infiltrate with necrosis; and 4, widespread infiltrate with hemorrhage and/or vasculitis. The pathological score was calculated according to the degree of heart graft injury in each recipient mouse.

### Immunohistochemistry assessment

As described above, 5-μm heart tissue slices were obtained from the paraffin-embedded grafts. After programmed dewaxing, the slices were first incubated with ethylene diamine tetra-acetic acid (EDTA) antigen repair solution (Solarbio, Beijing, China), and antigen repair was conducted for 15 min at 100 °C in a microwave oven. They were then cultured with 3% hydrogen peroxide to eliminate endogenous peroxides. After being blocked with 10% goat serum, the sections were incubated with rabbit anti-mouse CD4 (dilution at 1:1,000, Abcam), IFN-γ (dilution at 1:100, ABclonal), IL-17A (dilution at 1:100, ABclonal), and Foxp3 (dilution at 1:100, affinity) antibody overnight at 4 °C and then with 100 μL of enhanced enzyme-labeled goat anti-rabbit IgG polymer (DAB kit, ZSGB-BIO, Beijing, China) for 20 min the next day at room temperature. Finally, the slices were stained with hematoxylin for 1 min and then observed under an optical microscope to evaluate the severity of CD4^+^ T cell infiltration. The percentage of the area of the section occupied by positive staining using ImageJ software.

### Enzyme-linked immunosorbent assay

The serum levels of interferon gamma (IFN-γ), IL-17, and IL-10 in the recipient mice were measured using their corresponding ELISA kits (DAKEWE, Shenzhen, China) in accordance with the manufacturer’s instructions. In this experiment, 100 μL serum samples were required for each test well, and three representative cytokines were tested in this experiment. CD4^+^ T cells were isolated from the C57BL/6 mice and co-cultured with or without ERC-Exos or SIRT6-KD-ERC-Exos in 12-well plates. Then, the levels of IFN-γ, IL-17, and IL-10 in the cell supernatants were detected using their corresponding ELISA kits (DAKEWE, Shenzhen, China) in accordance with the manufacturer’s instructions.

### Glutamine uptake analysis

The extracted mouse naïve CD4^+^ T cells were placed in 24-well plates, with about 10^6^ cells per well, which were set into three groups, namely, untreated group, ERC-Exo treatment group, and ERC-SIRT6-KD-Exo treatment group. Each group was added with the same components necessary for CD4^+^ T cell activation, including but not limited to glutamine (2 mM), CD3 (5 μg/mL), CD28 (3 μg/mL), and IL-2 (50 U/mL). After 24 h of different treatments, supernatant and cells were collected separately. Glutamine contents of naïve CD4^+^ T cells were measured by use of a glutamine content measuring kit (Solarbio, Cat. NO.: BC5305, Beijing) in accordance with the manufacturer’s protocol. The OD value of samples at A450 was tested by using a multifunctional enzyme label instrument. Then, the glutamine content was calculated according to the manufacturer’s protocol. Additionally, the content of glutamine in the supernatant was also detected. Because the total glutamine content added to the culture medium among the three groups is the same, the difference of glutamine uptake by naïve CD4^+^ T cells can be obtained by comparing the glutamine content in supernatant and cells, respectively.

### α-ketoglutaric acid (α-KG) content detection

Naïve CD4^+^ T cells were collected after 24 h of different treatments, as in the previous treatment of glutamine content detection. α-KG contents of naïve CD4^+^ T cells were measured by use of an α-KG content measuring kit (Solarbio, Cat. NO.: BC5425, Beijing) in accordance with the manufacturer’s protocol. The OD value of samples at A340 was tested by using a multifunctional enzyme label instrument. Then, the α-KG content was calculated according to the manufacturer’s protocol.

### Flow cytometry analysis

Flow cytometry was performed to identify the phenotype of the ERCs and detect the immune cell population in the spleens of the recipient mice. The collected cells were divided into 100 μL single cell suspensions and then stained with fluorescent-labeled antibodies, including Zombie Dye, anti-CD14-FITC, anti-CD90-PE, anti-CD-79a-PE, anti-HLA-DR-FITC, anti-CD73-FITC, anti-CD44-APC, anti-CD4-FITC, anti-CD25-PE, anti-IL-17A-Percp-cy5.5, anti-FOXP3-APC, and anti-IFN-γ-PE, which were purchased from eBioscience (Thermofisher, United States) and BioLegend (Biolegend, United States). Finally, the percentage of different cells was analyzed using FlowJ software. The columnar statistical chart was made by Graphpad V8.0 software.

### Immunofluorescence staining

ERCs with a good growth state at passage 5 were adopted. The day before, the suspended ERCs were inoculated in a 12-well plate containing round coverslips, with a density of about 5 × 10^4^ cells/mL/well. When the cell aggregation reached 80%–90%, the cells were taken out and washed twice with PBS, then 1 mL of 4% paraformaldehyde fixed solution was added to each well for cell fixation at room temperature for 15 min, then PBS was washed twice, and 1% BSA blocking solution was added for nonspecific antigen blocking at room temperature for 1 h. Subsequently, the excess BSA blocking solution was sucked off, and CD73 antibody (dilution at 1:100, Abcam) was added for incubation at 4 °C overnight. The next day, the incubated antibody was taken out and returned to room temperature for 30 min. After PBS washing twice, the corresponding cy3-labeled secondary antibody (dilution at 1:50, Proteintech) was incubated for 1 h at room temperature in the dark. After PBS washing twice, the round coverslip was taken out and dripped with neutral resin containing 4,6-diamino-2-phenyl indole (DAPI) staining for sealing. The cells were observed under a fluorescence microscope.

### Exosome phagocytosis detection

The uptake of exosomes by naïve CD4^+^ T cells was determined as follows. ERC-Exos and SIRT6-KD-ERC-Exos were incubated with 1 μM PKH26 (Solarbio, Beijing, China) for 15 min at 37 °C and then centrifuged at 100,000 *g* for 70 min to remove unbound dye. Naïve CD4^+^ T cells were acquired from C57BL/6 mouse spleens and co-cultured with PKH-26 labeled exosomes for 6 h. The treated cells were washed twice with PBS and fixed with 4% paraformaldehyde. Following DAPI staining, the cells were observed under a confocal microscope.

### Co-culture of ERC-Exos and C57BL/6 mouse naïve CD4^+^ T cells *in vitro*

Naïve CD4^+^ T cells were extracted from C57BL/6 mice and co-cultured with or without ERC-Exos or SIRT6-KD-ERC-Exos in 96-well plates to investigate whether SIRT6-expressing ERC-Exos can influence CD4^+^ T cell differentiation *in vitro*. In brief, a suspension of naïve CD4^+^ T cells was prepared. The sacrificed spleens were ground and individually filtered using sterilized meshes (100 meshes). After the red blood cells were lysed, the remaining cells were resuspended and counted. Based on the number of cells, CD4^+^ (L3T4) T cell-sorting magnetic beads (Miltenyi biotec, Germany) with corresponding volumes were added and allowed to stand for 10 min for complete mixing. The mixed liquid was then filtered twice by gravity under a magnetic field. Subsequently, the filter column was washed with culture medium to obtain naïve CD4^+^ T cells. Each well was covered with 2 × 10^5^ naïve CD4^+^ T cells, and the cells were cultured in 200 μL of Roswell Park Memorial Institute 1,640 (RPMI-1640) medium. Finally, ERC-Exos or SIRT6-KD-ERC-Exos were added to different groups for co-culture, and flow cytometry was conducted after 72 h.

### Naïve CD4^+^ T cell activation detection

To explore whether SIRT6-expressing ERC-Exos affect naïve CD4^+^ T cell activation *in vitro*, we extracted naïve CD4^+^ T cells from C57BL/6 mice and co-cultured them with or without ERC-Exos or SIRT6-KD-ERC-Exos in 96-well plates for 12–24 h. After being co-cultured, T cells were collected for flow cytometry. Specifically, the collected cells were divided into 100 μL single cell suspensions and then stained with fluorescent-labeled antibodies, including anti-CD4-FITC and anti-CD25-PE, which were purchased from eBioscience (Thermofisher, United States) and BioLegend (Biolegend, United States). Finally, the percentage of different cells was analyzed using FlowJ software.

### Western blot

CD4^+^ T cells *in vitro* from the untreated, ERC-Exo-treated, and SIRT6-KD-ERC-Exo-treated groups were washed and then lysed in radio immunoprecipitation assay (RIPA) lysis buffer containing a protease inhibitor. After the lysis, the lysate was centrifuged, then the supernatant was sucked, and the concentration of the sample was measured by the BCA protein assay kit. Subsequently, loading buffer (Solarbio, Beijing, China) was added and the protein sample was denatured by boiling. The equal mass protein samples were separated by sodium dodecyl sulfate polyacrylamide gel electrophoresis (SDS-PAGE) and then transferred to a polyvinylidene fluoride (PVDF) membrane. The protein-containing membrane after transfer was blocked with bovine serum albumin solution and then incubated with primary antibodies at appropriate dilution (SIRT6, 1:2000, abcam; c-Myc, 1:3,000, Affinity; ASCT2, 1:750, ABclonal; GLS1, 1:1,000, Affinity; p-S6K1, 1:500, ABclonal; S6K1, 1:1,000, ABclonal) at 4 °C overnight. The next day, the membrane was washed in tris-buffered saline with tween-20 (TBST) and then incubated in the corresponding secondary antibody dilution (goat anti-rabbit, 1:2000, Affinity) at room temperature. Finally, the membrane was washed with TBST and incubated with an enhanced chemiluminescence solution (Sparkjade, Jinan, China) for several seconds. The membrane was photographed and analyzed on an exposure platform. The quantitative analysis was performed by comparing the ratio of target proteins/internal proteins in different groups using ImageJ.

### Statistical analysis

Experimental data are presented as mean ± SD. Differences between multiple groups were calculated using one-way analysis of variance (ANOVA). The survival curve of the heart graft was constructed using the Kaplan–Meier cumulative survival method, and differences among groups were analyzed using the log-rank (Mantel–Cox) test. In the data charts, one asterisk represents *p* ≤ 0.05, two asterisks represent *p* ≤ 0.01, and three asterisks represent *p* ≤ 0.001. Differences with *p* ≤ 0.05 were considered statistically significant. GraphPad Prism (version 8.0) was used for the statistical analyses.

## Results

### Characterization of ERCs

**FIGURE 1:**
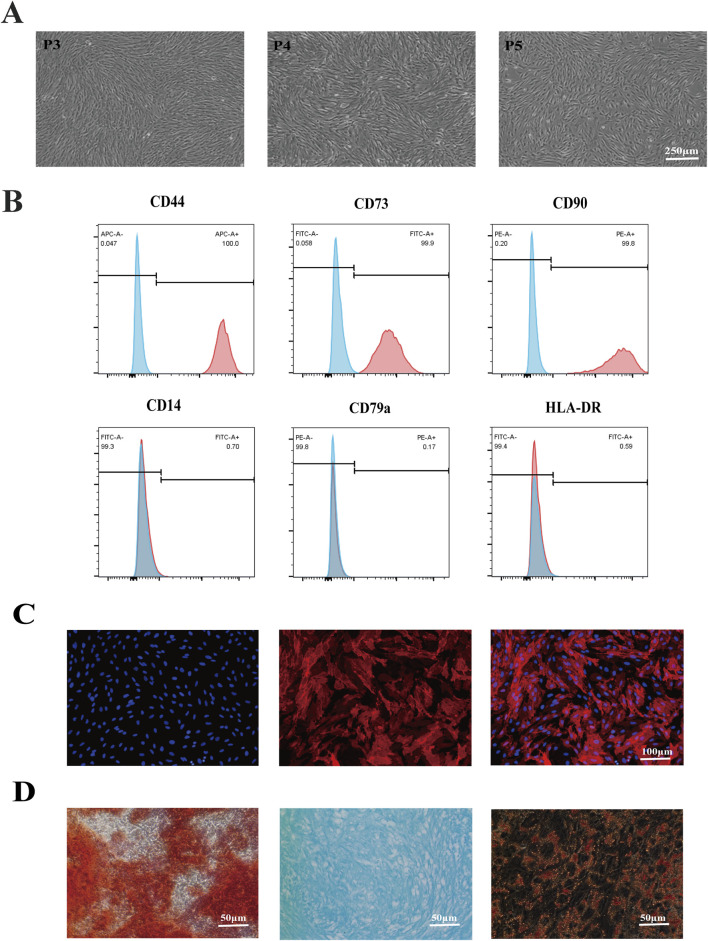
Identification of ERC features. **(A)** Morphology of ERCs at passage 3 to passage 5 (magnification 40×). ERCs were spindle-shaped, fibroblast-like, colony-forming ability and the doubling time was about 24 h. **(B)** Flow cytometry analysis of surface markers of ERCs. **(C)** Immunofluorescence staining of surface markers CD73 of ERCs (magnification 100×). **(D)** The results of three-lineage (osteogenic, chondrogenic, and adipogenic) differentiation of ERCs were stained by alizarin red, alixin blue and oil red O respectively (magnification 200×).

The ERCs’ morphology at passages 3–5 was used to assess their purity after being extracted from the volunteers’ menstrual blood. Results showed that the ERCs were spindle shaped, fibroblast-like, and colony-forming (Figure 1A). Furthermore, a significant rate of proliferation was indicated by the doubling time, which was roughly 24 h. The stem cell characteristics of the ERCs were further evaluated using common MSC surface markers. At passage 5, the ERCs were detached and stained with the MSC surface markers CD14, CD79a, HLA-DR, CD44, CD73, and CD90. Consistent with our previous reports, the ERCs showed high expression levels of CD44, CD73, and CD90 but no expression of CD14, CD79a, and HLA-DR (Figure 1B). Among these MSC surface markers, CD73, a representative ERC positive marker, was also selected for immunofluorescence staining (Figure 1C) to further verify the stem cell characteristics of ERCs for supporting these flow cytometry analyses. Additionally, ERCs’ strong osteogenic, chondrogenic, and adipogenic differentiation abilities were confirmed by the three-lineage differentiation experiment (Figure 1D). These findings demonstrated that the ERCs used in our study were MSCs with stable stem cell properties, the ability to cling to the culture dish, and the capacity to multiply quickly.

### Construction of ERC-Exos with low expression of SIRT6

**FIGURE 2:**
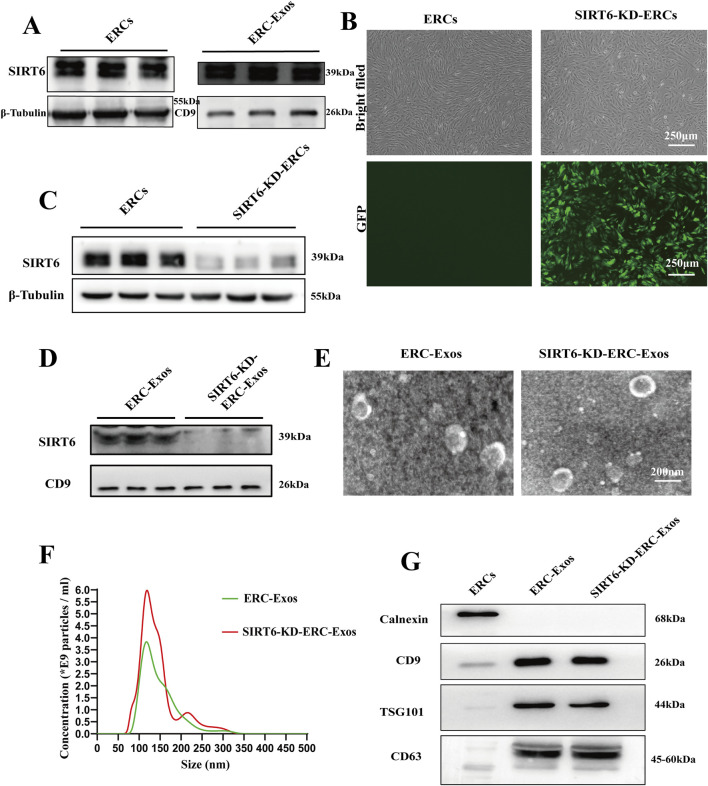
Isolation and identification of ERC-Exos and SIRT6-KD-ERC-Exos. **(A)** The expression level of SIRT6 in ERCs and ERC-Exos. **(B)** Morphology of ERCs and SIRT6-KD-ERCs at passage 2 (magnification 40×). The successful transduction of ERCs with shRNA-SIRT6 lentivirus was shown by GFP. **(C)** The expression level of SIRT6 in ERCs and SIRT6-KD-ERCs. **(D)** The expression level of SIRT6 in ERC-Exos and SIRT6-KD-ERC-Exos. **(E)** TEM revealed that both ERC-Exos and SIRT6-KD-ERC-Exos exhibited a bilayer membrane structure with a typical cup-shaped morphology. **(F)** Nanoparticle tracking analysis images of ERC-Exos and SIRT6-KD-ERC-Exos. Both ERC-Exos and SIRT6-KD-ERC-Exos were in the size range from 30 to 200 nm, and there is no difference between the two groups. **(G)** Western blot analysis of Calnexin, CD9, TSG101, and CD63 in ERCs, ERC-Exos, and SIRT6-KD-ERC-Exos (representative of three independent experiments).

In order to determine whether ERC-Exos can express SIRT6, we first identified the subcellular localization of SIRT6 in ERCs. The results showed that, consistent with previous reports, SIRT6 could be expressed in the nucleus and cytoplasm of ERCs (Supplementary Figure S1). The expression of SIRT6 in the ERC-Exos was measured using Western blot analysis. Results showed that SIRT6 did express in ERC-Exos (Figure 2A). To construct ERC-Exos with low SIRT6 expression, ERCs were transduced using a shRNA-SIRT6 lentivirus. At 72 h after transduction, GFP expression was detected in more than 90% SIRT6-KD-ERCs, and knocking down SIRT6 in ERCs would not cause changes in cell morphology (Figure 2B). Further examination showed that the expression of SIRT6 in ERCs was significantly decreased after transduction (Figure 2C). Subsequently, the supernatants were centrifuged to obtain SIRT6-KD-ERC-Exos. The result showed that SIRT6 expression was significantly knocked down and almost undetectable in SIRT6-KD-ERC-Exos (Figure 2D). Subsequently, exosomes purified from the supernatant of ERCs were identified using TEM and NTA. As previously described, TEM found that ERC-Exos exhibited a bilayer membrane structure with a typical cup-shaped morphology (Figure 2E). NTA showed that the majority of ERC-Exos were in the size range of 30–200 nm (Figure 2F). Moreover, the characteristics of SIRT6-KD-ERC-Exos were identified, and the findings were consistent with those of exosomes obtained from un-transfected ERCs. The results suggested that knocking down SIRT6 in ERC-Exos did not affect the morphological characteristics or size distribution of these exosomes (Figures 2E,F). Meanwhile, Western blot analysis identified exosome-specific markers, including CD9, CD63, and TSG101, but not the non-exosome marker calnexin in the ERC-Exo and SIRT6-KD-ERC-Exo groups (Figure 2G). These data indicated that ERC-Exos could express SIRT6 and that knocking down SIRT6 did not alter the basic characteristics of ERC-Exos.

Then, at the beginning of subsequent experiments, we set up three groups (ERC-Exos, SIRT6-NC-ERC-Exos, and SIRT6-KD-ERC-Exos) to identify exosomes and compare their functions. Results, as shown in the Supplementary Figure S2A, there was no difference in exosome morphology, particle size distribution, and surface protein markers among the three groups. Subsequently, we also compared the functions of the three groups. We executed the naïve CD4^+^ T cell differentiation experiment *in vitro*. The results showed that there was no difference in differentiation of CD4^+^ T cells between ERC-Exos and SIRT6-NC-ERC-Exos groups after treatment (Supplementary Figure S2B). Taken together, these data indicated that ERC-Exos and SIRT6-NC-ERC-Exos were identical in shape, size, and function.

### SIRT6 mediated ERC-Exos to alleviate AR in transplant recipients

**FIGURE 3:**
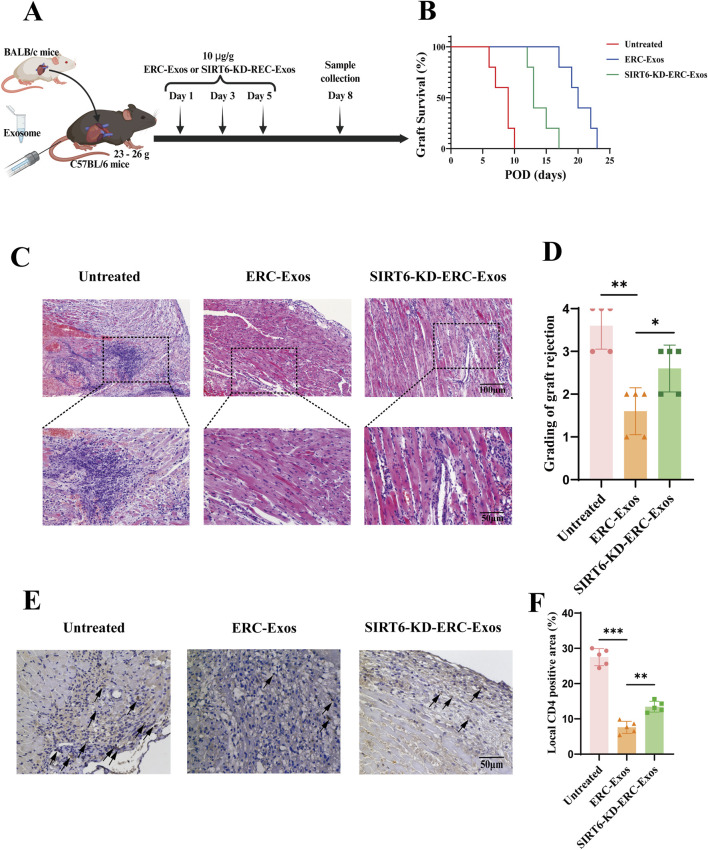
SIRT6 mediated ERC-Exos to prolong graft survival and alleviate acute allograft rejection. **(A)** A schema of *in vivo* study design. **(B)** Kaplan-Meier curves of allograft survival of cardiac transplants, in which BALB/c hearts were transplanted into C57BL/6 recipients. The allograft survival was significantly prolonged in ERC-Exo-treated group when compared with the untreated group, and the therapeutic effect was weakened in the SIRT6-KD-ERC-Exo-treated group (n = 5 per group). Statistics by Log-rank test. **(C)** Representative histology of cardiac allografts on post-operative day (POD) 8 (magnification 100× and 200× respectively). ERC-Exo-treated recipients showed a milder lymphocytes infiltration and myocyte damage compared with untreated mice, while SIRT6-KD-ERC-Exo treatment slightly reversed these changes (n = 5 per group). **(D)** Pathological score of graft rejection in different groups (n = 5 per group). Score criteria: 0, no change; 1, minimum change; 2, mild change; 3, moderate change; and 4, marked change compared with normal tissues. **(E)** Representative graft sections for immunohistological staining of intra-graft CD4^+^ T cell infiltration (magnification 200×). ERC-Exo-treated recipients revealed a milder CD4^+^ T cell infiltration compared with untreated mice, while knocking down SIRT6 in ERC-Exos slightly reversed these changes (n = 5 per group). The positive staining areas were shown by arrows. **(F)** Quantitative analysis of CD4^+^ T cell proportion in IHC. Statistics by one-way ANOVA. *p < 0.05, **p < 0.01, ***p < 0.001, ns = non-significant. For all panels, the bar graphs represent mean ± SD.

To determine whether SIRT6 mediates the therapeutic effect of ERC-Exos on AR, C57BL/6 recipient mice were transplanted with heart grafts from BALB/c donor mice and divided into three groups, namely, untreated, ERC-Exo-treated, and SIRT6-KD-ERC-Exo-treated group. As shown in Figure 3A, C57BL/6 recipient mice were injected with ERC-Exos or SIRT6-KD-ERC-Exos on the first, third and fifth day after operation, and at the same time, the untreated group mice were injected with the same volume of PBS. The results showed that ERC-Exos significantly prolonged allograft survival, and this effect was related to SIRT6 expression (Figure 3B). Specifically, the allograft survival time was significantly longer (*p* < 0.05) in the ERC-Exo-treated group (20.2 ± 2.4° days) than in the untreated group (8.2 ± 1.6° days). However, the allograft survival time was significantly shorter (*p* < 0.05) in the SIRT6-KD-ERC-Exo-treated group (14 ± 2° days) than in the ERC-Exo-treated group (20.2 ± 2.4° days). Graft pathology (Figure 3C) revealed that the grafts in the untreated group showed severe rejection with vasculitis and massive cell infiltration. After ERC-Exo treatment, the lesions in the grafts were significantly attenuated, with almost normal pathology on POD 8. However, SIRT6 knockdown reduced the therapeutic effect of ERC-Exos in mediating allograft protection, and the grafts showed typical features of AR. Graft pathology was scored according to accepted diagnostic criteria for heart rejection (Stewart et al., 2005). According to the findings, the untreated group had the highest pathological lesion score, followed by the SIRT6-KD-ERC-Exo-treated group. According to Figure 3D, the group that received ERC-Exo had the lowest pathological lesion score. In addition, ERC-Exo treatment significantly reduced intra-graft CD4^+^ T cell infiltration, but this effect was markedly inhibited when SIRT6 was knocked down in ERC-Exos (Figures 3E,F).

**FIGURE 4:**
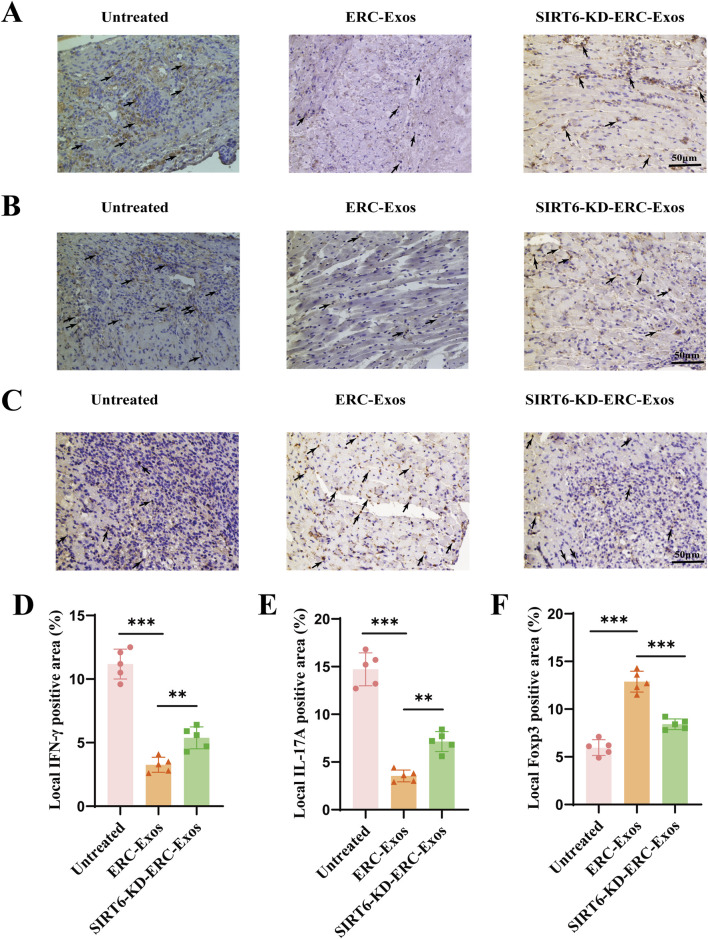
SIRT6 mediated ERC-Exos to regulate CD4^+^ T cell differentiation in the graft, alleviating the inflammatory infiltration. Representative graft sections for infiltration of different subsets of CD4^+^ T cells and immunohistochemical staining of cytokines in the graft (magnification 200×). IFN-γ **(A)** and IL-17A **(B)** were used to stain Th1 and Th17 cells and their corresponding cytokines in the graft, respectively. Foxp3 **(C)** was used to stain the Treg infiltration in the graft. The positive staining areas were shown by arrows. Quantitative analysis of IFN-γ **(D)**, IL-17A **(E)**, and Foxp3 **(F)** positive cell proportion in IHC via ImageJ software.

In order to further verify whether the therapeutic effect of ERC-Exos is related to its regulation of CD4^+^ T cell differentiation in the graft, we performed immunohistochemical staining in different (untreated, ERC-Exo treated, and SIRT6-KD-ERC-Exo treated) groups of mouse heart grafts. Different from the detection of CD4^+^ T cell infiltration level, we paid more attention to the different subtypes and cytokine levels of CD4^+^ T cells in the grafts. Results showed that in the untreated group, more inflammatory cytokines such as IFN-γ and IL-17A were found in the transplanted heart of mice, and the levels of these two cytokines decreased significantly after ERC-Exo treatment (Figures 4A,B,D,E). However, when SIRT6 was knocked down in ERC-Exos, this trend was obviously reversed (Figures 4A,B,D,E). In addition, we also detected the infiltration of anti-inflammatory cell Tregs in the graft. The infiltration distribution of Tregs showed an opposite trend. Treg infiltration was less in untreated grafts, but the proportion of Tregs in grafts increased significantly after ERC-Exo treatment. Remarkably, compared to the ERC-Exo treatment group, Treg infiltration in these grafts dramatically diminished after SIRT6-KD-ERC-Exos were administered (Figures 4C,F). These findings indicated that via modulating the proportion between different CD4^+^ T cell subtypes in the graft, SIRT6 mediated the therapeutic benefits of ERC-Exos in alleviating AR of cardiac allografts.

### SIRT6 mediated ERC-Exos to remodel the proportion of different CD4^+^ T cell subtypes in transplant recipients

**FIGURE 5:**
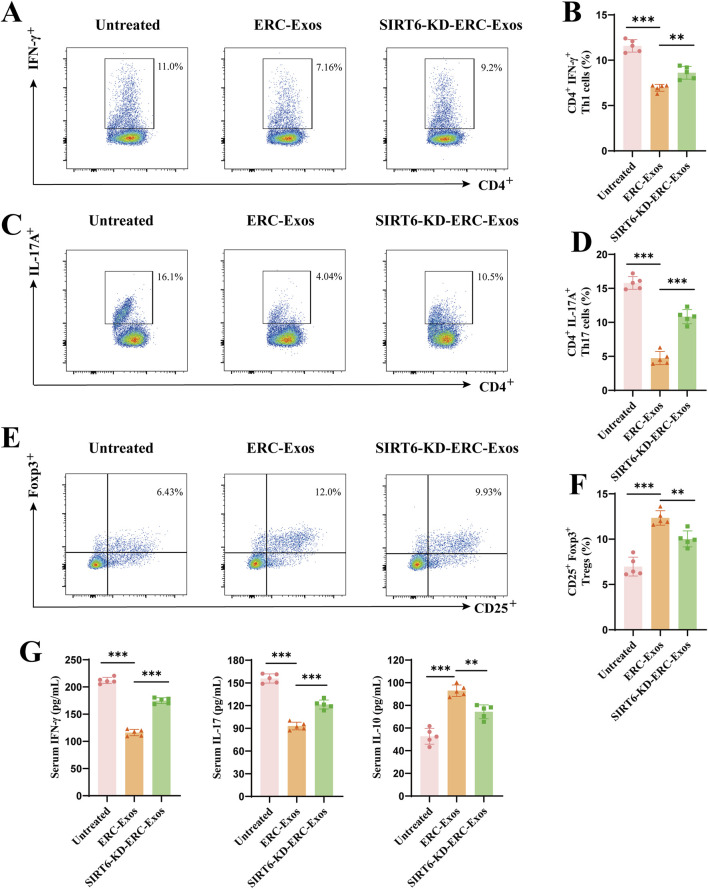
SIRT6 mediated ERC-Exos to remodel CD4^+^ T cell differentiation and serum cytokine secretion level in recipient mice. Single-cell suspensions of splenocytes obtained from the untreated, ERC-Exo-treated, and SIRT6-KD-ERC-Exo-treated groups were analyzed for the frequency of CD4^+^IFN-γ^+^, and CD4^+^IL-17A^+^ cells by flow cytometry, gated on live cells. The representative pseudocolor plots **(A,C)** and statistical graphs **(B,D)** were depicted (n = 5 per group). In addition, the frequency of CD25^+^Foxp3^+^ cells obtained from the three groups were also analyzed by flow cytometry, gated on live cells and CD4^+^ cells. The representative pseudocolor plots **(E)** and statistical graphs **(F)** were depicted (n = 5 per group). **(G)** The representative CD4^+^ T cell serum cytokine levels in the recipient mice across the three groups (n = 5 per group). Specifically, compared with untreated group, ERC-Exo treatment could significantly decrease serum pro-inflammatory cytokine (IFN-γ and IL-17) level and increase IL-10 secretion, while knocking down SIRT6 in ERC-Exos slightly reversed these trends. Statistics by one-way ANOVA. *p < 0.05, **p < 0.01, ***p < 0.001, ns = non-significant. For all panels, the bar graphs represent mean ± SD.

The above results showed that the SIRT6-expressing ERC-Exos affected the CD4^+^ T cell infiltration and the degree of pathological damage in these grafts. To explore the reasons behind this therapeutic effect, we determined the proportions of CD4^+^ T cell subtypes, including Th1, Th17, and Tregs, in the peripheral lymphoid organs of recipient mice. As shown in Figures 5A–D, the proportions of Th1 and Th17 cells were significantly lower in the ERC-Exo-treated and SIRT6-KD-ERC-Exo-treated groups than in the untreated group. Compared with the ERC-Exo-treated group, the SIRT6-KD-ERC-Exo-treated group had significantly higher proportions of Th1 and Th17 cells in the spleens of the recipient mice. Meanwhile, the Treg proportion was significantly higher (*p* < 0.001) in the ERC-Exo-treated group than in the untreated group (Figures 5E,F). Similarly, the Treg population was significantly lower (*p* < 0.01) in the SIRT6-KD-ERC-Exo-treated group than in the ERC-Exo-treated group (Figures 5E,F). Subsequently, the serum levels of cytokines, including IFN-γ, IL-17, and IL-10, were examined in the different groups. Compared with the other groups, the ERC-Exo-treated group had the lowest levels of the pro-inflammatory cytokines IFN-γ and IL-17 (IFN-γ: untreated group vs. ERC-Exo-treated group, *p* < 0.001; ERC-Exo-treated group vs. SIRT6-KD-ERC-Exo-treated group, *p* < 0.001; IL-17: untreated group vs. ERC-Exo-treated group, *p* < 0.001; ERC-Exo-treated group vs. SIRT6-KD-ERC-Exo-treated group, *p* < 0.01) and the highest anti-inflammatory cytokine IL-10 (untreated group vs. ERC-Exo-treated group, *p* < 0.001; ERC-Exo-treated group vs. SIRT6-KD-ERC-Exo-treated group, *p* < 0.01, Figure 5G). Although the SIRT6-KD-ERC-Exo-treated group had lower IFN-γ and IL-17 secretion and higher IL-10 secretion than that in the untreated group, the therapeutic effect was still stronger in the ERC-Exo-treated group than in the SIRT6-KD-ERC-Exo-treated group (IFN-γ: ERC-Exo-treated group vs. SIRT6-KD-ERC-Exo-treated group, *p* < 0.001; IL-17: ERC-Exo-treated group vs. SIRT6-KD-ERC-Exo-treated group, *p* < 0.01; IL-10: ERC-Exo-treated group vs. SIRT6-KD-ERC-Exo-treated group, *p* < 0.01, Figure 5G). These data indicated that SIRT6 mediated ERC-Exos to remodel the proportion of different CD4^+^ T cell subtypes and serum cytokine levels in transplant recipients.

### SIRT6 mediated ERC-Exos to modulate naïve CD4^+^ T cell differentiation *in vitro*

**FIGURE 6:**
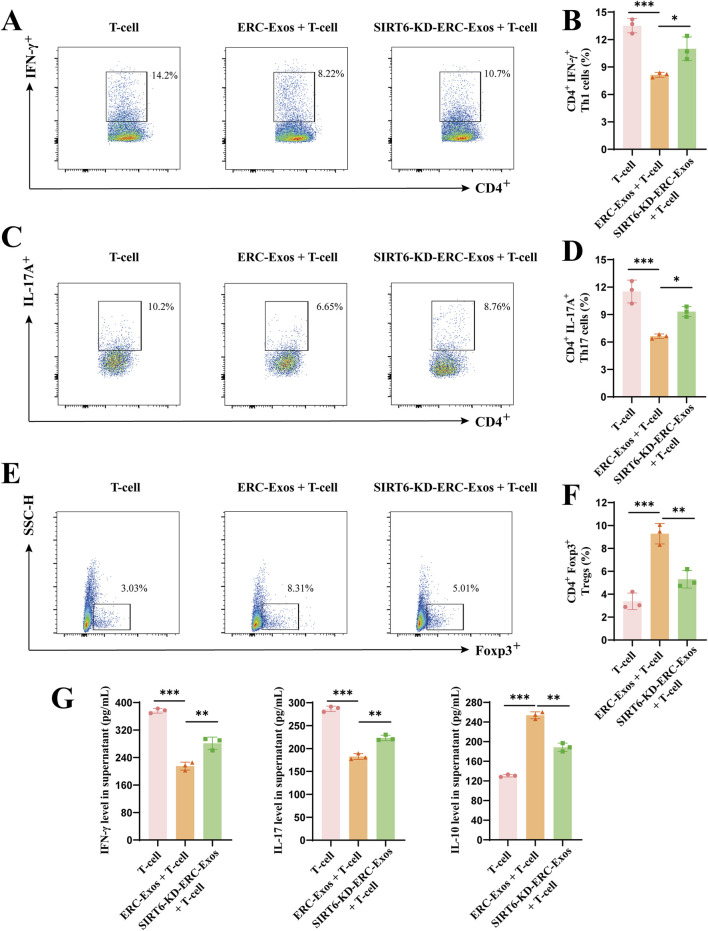
SIRT6 mediated ERC-Exos to modulate CD4^+^ T cell differentiation and cytokine secretion level *in vitro*. Naïve CD4^+^ T cells were extracted from C57BL/6 mice. In the medium added with CD3/CD28, they were co-cultured with/withou ERC-Exos and SIRT6-KD-ERC-Exo for 72 h. Single-cell suspensions were obtained from the T-cell, T-cell + ERC-Exo, and T-cell + SIRT6-KD-ERC-Exo groups and then were analyzed for the frequency of CD4^+^IFN-γ^+^, and CD4^+^IL-17A^+^ cells by flow cytometry, gated on live cells. The representative pseudocolor plots **(A,C)** and statistical graphs **(B,D)** were depicted (Repeat three independent holes). Similarly, the frequency of Foxp3^+^ cells obtained from the three groups were also analyzed by flow cytometry, gated on live cells and CD4^+^ cells. The representative pseudocolor plots **(E)** and statistical graphs **(F)** were depicted (Repeat three independent holes). **(G)** The representative cytokine levels of CD4^+^ T cell supernatant in the three groups (Repeat three independent holes). Compared with untreated group, ERC-Exo treatment could significantly decrease serum pro-inflammatory cytokine (IFN-γ and IL-17) level and increase IL-10 secretion, while absence of SIRT6 in ERC-Exos slightly reversed these trends. Statistics by one-way ANOVA. *p < 0.05, **p < 0.01, ***p < 0.001, ns = non-significant. For all panels, the bar graphs represent mean ± SD.

To further explore the role of SIRT6 in mediating ERC-Exo-induced naïve CD4^+^ T cell differentiation *in vitro*, we co-cultured ERC-Exos with naïve CD4^+^ T cells from the peripheral lymphoid organs of the C57BL/6 recipient mice. Firstly, the purity of isolated naïve CD4^+^ T cells was detected by flow cytometry after isolation. The results (Supplementary Figure S3) showed that the purity of CD4^+^ T cells isolated from C57BL/c mice spleens was more than 98%. Besides, the recognized markers of naïve CD4^+^ T cells (CD44^lo^CD62L^hi^) in mice were also used for staining, and the results showed that the purity of naïve CD4^+^ T cells was over 96% (Supplementary Figure S3). Because CD44CD62L can be used not only to identify naïve CD4^+^ T cells but also to identify effector T cells (CD44^hi^CD62L^lo^), the data show that effector T cells (0.02%) could be almost ignored in the newly extracted naïve CD4^+^ T cells of mice. Then, on the basis of the experimental grouping *in vivo*, the T-cell, T-cell + ERC-Exo, and T-cell + SIRT6-KD-ERC-Exo groups were established *in vitro*. Subsequently, the proportions of different CD4^+^ T cell subtypes were determined *in vitro* in the three groups. The T-cell group had the highest proportions of Th1 and Th17 cells and the lowest Treg proportion, whereas the T-cell + ERC-Exo group showed the opposite trend. Compared with the T-cell + ERC-Exo group, the T-cell + SIRT6-KD-ERC-Exo group had significantly higher proportions of Th1 and Th17 cells and lower Treg proportion (T-cell + ERC-Exo group vs. T-cell + SIRT6-KD-ERC-Exo group, Th1 cells: *p* < 0.05; Th17 cells: *p* < 0.05; Tregs: *p* < 0.01; Figures 6A–F). Finally, the secretion levels of cytokines IFN-γ, IL-17, and IL-10 were detected in the different groups. As shown in Figure 6G, the results were consistent with the experimental results *in vivo*; that is, ERC-Exo treatment induced cytokines to tilt in the direction of anti-inflammation, while the absence of SIRT6 in exosomes weakened this effect. These results indicated that SIRT6 mediated ERC-Exos to modulate naïve CD4^+^ T cell differentiation and cytokine secretion *in vitro*.

Subsequently, we further detected the level of cell death and proliferation during CD4^+^ T cell differentiation *in vitro*. We explored it by Annexin V/7-AAD dye and the results showed that there would be some cell death in the process of T cell differentiation. When ERC-Exo treatment was given, the proportion of apoptotic cells in T cells decreased obviously, and when SIRT6 was knocked down, the decreasing trend increased again (Supplementary Figure S4A,B). These data indicated that SIRT6 also played a certain role in mediating ERC-Exos to inhibit T cell death. As to proliferation during *in vitro* T cell differentiation experiments, we used Ki67 dye to detect the proliferation of different treatment groups. The results, as shown in the Supplementary Figure S4C, showed that ERC-Exo treatment obviously inhibited the excessive proliferation of T cells under inflammatory conditions, while the knockdown of SIRT6 in exosomes reversed this trend, which indicated that SIRT6 mediated its regulation of T cell proliferation in ERC-Exos.

### SIRT6 mediated ERC-Exos to inhibit naïve CD4^+^ T cell activation and mTORC1 activity by weakening c-Myc-dependent glutaminolysis

**FIGURE 7:**
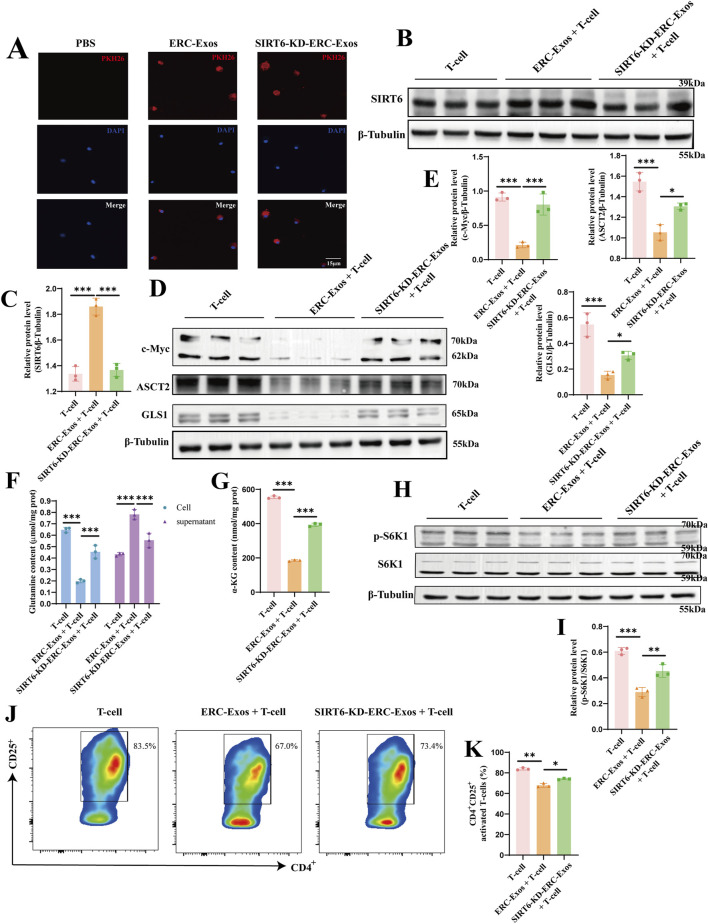
SIRT6 mediated ERC-Exos to inhibit naïve CD4^+^ T cell activation and mTORC1 activation via weakening c-Myc-dependent glutaminolysis. Firstly, naïve CD4^+^ T cells were acquired from C57BL/6 mouse spleen, then were co-cultured with PKH-26 labeled exosomes for 6 h. **(A)** Representative diagrams of ERC-Exos and SIRT6-KD-ERC-Exos phagocytosed by CD4^+^ T cells (red represents exosomes-labeled by PKH26, blue represents the naïve CD4^+^ T cell nucleus-labeled by DAPI; magnification 630×). Subsequently, ERC-Exos or SIRT6-KD-ERC-Exos were added into naïve CD4^+^ T cells respectively for 12 h, and then detected the expression level of SIRT6 in naïve CD4^+^ T cells via Western blot **(B)**. **(C)** Quantitative analysis of SIRT6 protein level. The key proteins of c-Myc-induced glutaminolysis **(D)** were detected via Western blot. **(E)** Quantitative analysis of c-Myc, ASCT2, and GLS1 protein level. Besides, the content of glutamine was detected in CD4^+^ T cells and supernatant 24 h after different treatments **(F)**. Because the glutamine content added in the culture medium was consistent, the glutamine uptake level of CD4^+^ T cells in different treatment groups could be evaluated by comparing the glutamine content in cells and supernatant, respectively. **(G)** The decomposition product α-KG of glutamine was further detected in these CD4^+^ T cells. **(H)** The activation state of mTORC1 in CD4^+^ T cells of different treatment groups was further detected. Finally, the activation level of naïve CD4^+^ T cells was analyzed for the frequency of CD25^+^ via flow cytometry. **(I)** Quantitative analysis of p-S6K1 protein level. The representative pseudocolor plots **(J)** and statistical graphs **(K)** were depicted (Repeat three independent holes). Statistics by one-way ANOVA. *p < 0.05, **p < 0.01, ***p < 0.001, ns = non-significant. For all panels, the bar graphs represent mean ± SD.

To further explore the mechanisms by which SIRT6 mediates ERC-Exos to modulate CD4^+^ T cell differentiation, we conducted an exosome phagocytosis experiment. Both ERC-Exos and SIRT6-KD-ERC-Exos were phagocytosed by naïve CD4^+^ T cells, with no significant difference in the level of phagocytosis (Figure 7A). Subsequently, the SIRT6 protein level in naïve CD4^+^ T cells were measured after co-culturing with ERC-Exos or SIRT6-KD-ERC-Exos for 12 h. Western blot analysis showed that ERC-Exo treatment significantly increased SIRT6 protein expression in naïve CD4^+^ T cells (Figures 7B,C). However, treatment with SIRT6-KD-ERC-Exos did not significantly change SIRT6 protein expression level in naïve CD4^+^ T cells (Figures 7B,C).

SIRT6 has been shown in earlier research to be able to block c-Myc transcription and translation in a variety of cell types. The transcription factor c-Myc is essential for T cell proliferation and differentiation. According to prior proteomic researches, c-Myc regulates the expression of important proteinases and amino acid transporters in immunocompetent T cells, with ASCT2 and GLS1 displaying the most notable alterations (Wang et al., 2011; Marchingo et al., 2020). To find out this change, we first used CD3/CD28 to stimulate the activation of naïve CD4^+^ T cells, and then treated them with ERC-Exos/SIRT6-KD-ERC-Exos, respectively, and then detected the transcription level of c-Myc in T cells. The results showed that after ERC-Exo treatment, the transcription level of c-Myc mRNA in naïve CD4^+^ T cells decreased significantly, and this decrease would be reversed with the knock-down of SIRT6 in exosomes (Supplementary Figure S5). Besides, the transcription level of ASCT2 and GLS1 mRNA in naïve CD4^+^ T cells also showed the same trend (Supplementary Figure S5). Then, we further use Western blot to analyze their expression at the translation level. The results showed that ERC-Exo treatment significantly inhibited c-Myc expression, whereas SIRT6-KD-ERC-Exo treatment restored this inhibitory function (Figures 7D,E). The downstream target proteins of c-Myc, including ASCT2 and GLS1, were also detected, and the result was consistent with the change in c-Myc protein expression (Figures 7D,E). In order to further clarify that this change is indeed related to SIRT6, we further explored it by using SIRT6 inhibitor OSS_128,167. In the experimental group where SIRT6 inhibitor interfered with ERC-Exo treatment, the protein levels of c-Myc, ASCT2, and GLS1 increased compared with ERC-Exo treatment (Supplementary Figure S6A,B). Subsequently, we detected the glutamine uptake of CD4^+^ T cells in different treatment groups. These results are consistent with the above changes in protein levels. Compared with the other two groups, the untreated group showed the highest glutamine intake level (Figure 7F). However, after ERC-Exo treatment, the glutamine uptake level of T cells decreased obviously, showing the lowest level (Figure 7F). Compared with ERC-Exo treatment, the uptake level of glutamine in T cells treated with SIRT6-KD-ERC-Exo increased, but it was still lower than that in the untreated group (Figure 7F). In addition, the content of α-KG in CD4^+^ T cells was further detected in the three groups, respectively. Consistent with the level of glutamine intake, the highest level of α-KG content was displayed in CD4^+^ T cells of the untreated group (Figure 7G). After ERC-Exo treatment, the content of α-KG in CD4^+^ T cells decreased significantly, but when SIRT6 in ERC-Exo was knocked down, the content of α-KG in T cells increased (Figure 7G). Similarly, after OSS_128,167 treatment, glutamine uptake and intracellular α-KG production of naïve CD4^+^ T cells were significantly inhibited compared with ERC-Exo treatment group (Supplementary Figure S6C,D). Previous studies have revealed that the activation state of mammalian target of rapamycin complex 1 (mTORC1) is regulated by glutaminolysis in various cells. Considering the decrease of glutamine uptake and its metabolite α-KG, we further explored the activation state of the mTORC1 signaling pathway through Western blot analysis. Similarly, the data showed that the ERC-Exo treatment inhibited the activation of the mTORC1 pathway in naïve CD4^+^ T cells, and this effect was related to the expression of SIRT6 in exosomes (Figures 7H,I). Administration of SIRT6-KD-ERC-Exos significantly reversed the inhibition of the mTORC1 pathway (Figures 7H,I). The Supplementary Figure S6E,F further proved this effect mediated by SIRT6 in ERC-Exos.

In order to further clarify that this effect is mediated by SIRT6, we used SIRT6 activator and SIRT6 inhibitor in Jurkat cell to explore the activated state of c-Myc/glutaminolysis/mTORC1 status respectively. The results showed that the level of c-Myc-dependent glutaminolysis increased after the activation of CD3/CD28 in Jurkat cell, which led to the activation of mTORC1 (Supplementary Figure S7). When SIRT6 activator was given, these effects were obviously inhibited (Supplementary Figure S7), indicating that SIRT6 did play a key role in inhibiting c-Myc-dependent glutaminolysis of T cells. Similarly, the opposite trend after administration of SIRT6 inhibitor further proves this effect (Supplementary Figure S7). These data demonstrated that SIRT6 did regulate the c-Myc/glutaminolysis/mTORC1 status in activated T cells.

We measured the activation level of naïve CD4^+^ T cells in light of earlier research showing that the production of important proteins linked to glutaminolysis is suppressed and that T cell activation is linked to glutaminolysis. The results showed that administration of ERC-Exos significantly restrained the activation of naïve CD4^+^ T cells compared with untreated T cells (T-cell + ERC-Exo group vs. T-cell group, *p* < 0.01, Figures 7J,K; Supplementary Figure S8). However, SIRT6 knockdown in ERC-Exos led to an increased activation of naïve CD4^+^ T cells (T-cell + ERC-Exo group vs. T-cell + SIRT6-KD-ERC-Exo group, *p* < 0.05, Figures 7J,K; Supplementary Figure S8). Taken together, these data showed that SIRT6 mediated ERC-Exos to inhibit naïve CD4^+^ T cell activation and mTORC1 activity by weakening c-Myc-dependent glutaminolysis.

## Discussion

Immunosuppressive medications are currently the primary strategy for managing AR following transplantation. As previously mentioned, it is impossible to overlook these medications’ limits with regard to infection, carcinogenesis, and chronic graft failure (Söderlund and Rådegran, 2015). So, safe and effective methods to alleviate AR must be explored. Clinical trials have shown that MSCs from different sources, such as umbilical cord-derived MSCs or bone marrow-derived MSCs, can be administered as injection therapy after kidney transplantation, and they all have obvious therapeutic effects in reducing the dosage of immunosuppressive drugs with no adverse reactions (Sun et al., 2018; Wei YC. et al., 2021). Besides, our preliminary work confirmed that ERCs and ERC-Exos can alleviate AR and prolong cardiac allograft survival in a mouse cardiac allograft model (Hu et al., 2021; Xu et al., 2024). Notably, previous studies have confirmed that the therapeutic effect of ERCs is gender-independent (Izawa et al., 2025; Lu et al., 2024), and it can play a therapeutic role in both female and male mice (Fathi-Kazerooni and Tavoosidana, 2019). Considering that female mice may have some extra-experimental interference due to the changes of the menstrual cycle and hormone level, this experiment adopts male mice for heart transplantation.

**FIGURE 8:**
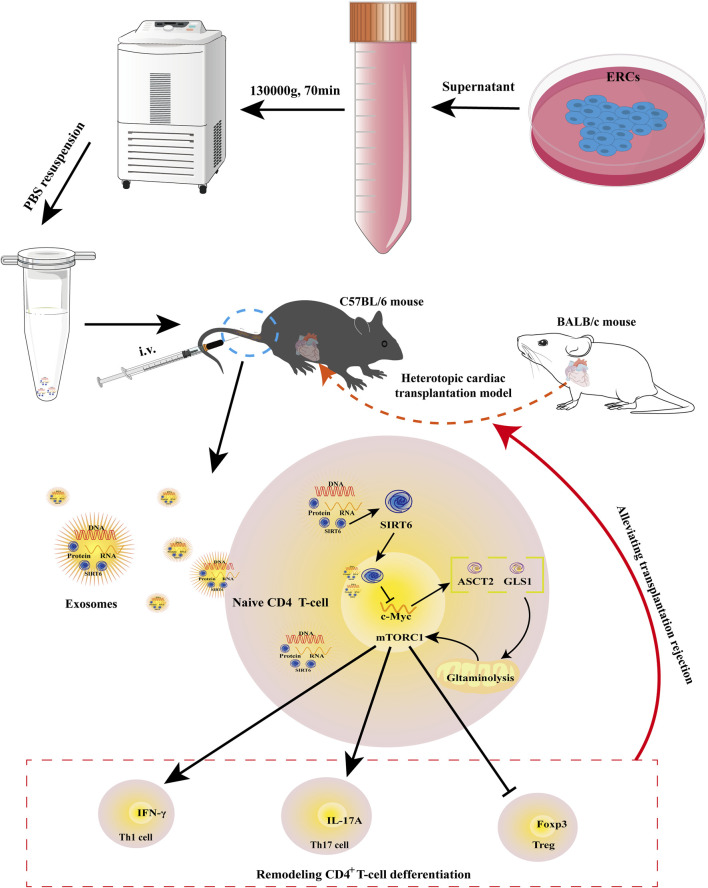
Schematic diagram of the therapeutic effects mediated by SIRT6-expressing ERC-Exos on AR. As previously described, ERC-Exos are obtained from the supernatant of ERCs by ultracentrifugation. Then, BALB/c hearts are transplanted into the abdomen of C57BL/6 recipients to construct a heterotopic cardiac transplantation model. And ERC-Exo treatment can alleviate AR in recipient mice by tail vein injection. In terms of mechanism, after the ERC-Exos are injected into recipient mice, they reach all parts of the body with blood circulation, including lesion sites or peripheral lymphoid organs, such as the spleen, and then can be phagocytized by peripheral naïve CD4^+^ T cells, leading to an increased SIRT6 expression level in these T cells. Increased SIRT6 in naïve CD4^+^ T cells can inhibit the transcription and translation of c-Myc through its histone deacetylation and then downregulate the key proteins of glutaminolysis, ASCT2 and GLS1. Thus, SIRT6-expressing ERC-Exos can weaken c-Myc-dependent glutaminolysis in naïve CD4^+^ T cells. Due to the weakened glutaminolysis, the activity of mTORC1, the downstream pathway of glutaminolysis process, is also inhibited, so the differentiation of naïve T cells into Th1/Th17 is weakened and Treg differentiation is increased.

In this study, we first found that SIRT6 expressed in ERC-Exos. We then used the shRNA-SIRT6 lentiviral to successfully obtain SIRT6-KD-ERC-Exos, a type of ERC-Exo with extremely low SIRT6 expression. Though using these two different exosomes to treat intra-abdominal heterotopic cardiac transplantation mice, we found that ERC-Exo treatment alleviated AR in these model mice. Specifically, ERC-Exo treatment prolonged graft survival, reduced the infiltration of inflammatory cell as well as cytokines in the graft, remodeled CD4^+^ T cell differentiation, and rectified the inordinate secretion of CD4^+^ T cell inflammatory cytokines. However, when SIRT6 was knocked down in ERC-Exos, these therapeutic effects were significantly weakened, resulting in a shorter graft survival, heavier intra-graft inflammatory cell and cytokines infiltration, disordered CD4^+^ T cell differentiation, and serum pro-inflammatory cytokine secretion. This cardioprotective effect may be attributed to the therapeutic effect of ERC-Exos mediated by SIRT6 on acute cardiac allograft rejection by remodeling peripheral CD4^+^ T cell differentiation. Follow-up mechanistic studies demonstrated that ERC-Exo treatment downregulated c-Myc protein expression, thereby weakening the uptake and utilization of glutamine. As mentioned earlier, c-Myc is a hub protein that can regulate the relative expression of key proteins related to glutaminolysis, especially ASCT2 and GLS1 (Marchingo et al., 2020). The expression levels of ASCT2 and GLS1 were verified to be downregulated, and the activation of the mTORC1 pathway was suppressed. Likewise, the expression level of these proteins, the uptake rate, and the glutamine utilization rate were all reversed while SIRT6 was knocked out in ERC-Exos. Taken together, these results indicated that it was SIRT6 in ERC-Exos that alleviated AR by weakening c-Myc-dependent glutaminolysis (Figure 8).

Exosomes are 30–200 nm membrane extracellular vesicles composed of various proteins, enzymes, transcription factors, lipids, extracellular matrix proteins, receptors, and nucleic acids (Kalluri and LeBleu, 2020). Previous studies have shown that ERC-Exos can promote angiogenesis, anti-apoptosis, and immunoregulation through a variety of microRNAs and proteins contained in it (Marinaro et al., 2019). Our previous research found that loading siSLAMF6 within ERC-Exos can effectively prolong the graft survival time in cardiac transplantation mice (Xu et al., 2024). Interestingly, in this study, we found that compared with the untreated group, ERC-Exo treatment can also significantly prolong the graft survival time, and significantly inhibit the trend of CD4^+^ T cells differentiating into pro-inflammatory subtypes (Xu et al., 2024). This means that there are some therapeutic molecules in ERC-Exos. Although ERC-Exos with artificial modification does show better therapeutic effect, its large-scale development is limited due to the complicated artificial modification process of foreign bodies. Therefore, exploring the self-mechanism of ERC-Exo therapeutic effect is imperative.

With the discovery of transcription factors in exosomes (Kalluri and LeBleu, 2020; Qiu et al., 2018), some proteins that were located in the nucleus and could enter/leave the nucleus, including the SIRT family proteins, were gradually explored in exosomes. The SIRT family proteins have seven members (SIRT1-7) with different subcellular localization and function (Warren and MacIver, 2019). These proteins play many important roles in cell metabolism, anti-oxidative stress and DNA repair (Warren and MacIver, 2019). Among them, the roles of SIRT1 and SIRT3 in adaptive immune regulation have been revealed (Warren and MacIver, 2019). A recent study found that the decrease of SIRT1 expression was related to the severity of inflammation and tubulitis after renal transplantation (Aksungur et al., 2025). Because SIRT1 and SIRT3 are located in the nucleus and mitochondria, respectively, there are few previous studies on them in exosomes. Encouragingly, with the development of research, the extracellular exploration of SIRT3 may be further developed with the help of the research hotspot of mitochondria transfer. Besides, SIRT6 also showed good anti-inflammatory potential (Pillai and Gupta, 2020). For instance, inhibiting SIRT6 reduced the percentage of Tregs in the peripheral blood and synovial fluid in rheumatoid arthritis (Wang et al., 2019). However, whether SIRT family proteins can be expressed in extracellular structure is still a puzzle. Until recent years, Chamberlain *et al.* found that SIRT2 is expressed in oligodendrocyte-derived exosomes and deacetylated (Chamberlain et al., 2021). Wei *et al.* also demonstrated that SIRT6 expressed in mouse bone marrow-derived MSC exosomes and alleviates aortic calcification by deacetylating HMGB1 (Wei WQ. et al., 2021). These reports aroused our interest in exploring the role and function of SIRT6 in ERC-Exos for immunotherapy. Hou *et al.* also reported that SIRT6 can be released from the nucleus and accumulate in the cytoplasm, and this effect can be amplified by palmitic acid treatment (Hou et al., 2022). Combining these reports with our results, we speculate that the therapeutic effect of ERC-Exos can be improved by pretreatment with palmitic acid followed by exosome collection. As for other SIRT family proteins, based on their anti-oxidative stress function, they may also have the potential to regulate immunity through a series of intracellular physiological activities, but the specific mechanism may be different and needs further exploration.

Previous studies have reported that T cells can change their differentiation fate or function by phagocytosing exosomes secreted by other cells (Wang et al., 2018; Bolandi et al., 2020). In the present study, the ERC-Exos injected into the recipient mice were distributed into all parts of the body via blood circulation, including lesion sites or peripheral lymphoid organs, such as the spleen, and then phagocytosed by peripheral naïve CD4^+^ T cells, leading to an increase in SIRT6 expression, thus exerting its therapeutic effects. As previously reported, SIRT6 may also play a deacetylating role in the exosomes (Wei WQ. et al., 2021). SIRT6 plays a therapeutic role in many immune-related diseases through deacetylation (Li et al., 2022; Pillai and Gupta, 2020). For example, Oraby *et al.* found that SIRT6 improves cell inflammation and blepharoptosis caused by ulcerative colitis in acetic acid-treated rats by deacetylating FoxC1 (Oraby et al., 2024). Studies have shown that SIRT6 can decrease c-Myc activity by promoting its deacetylation at the ninth lysine site of H3 histone (H3K9) and the 56th lysine site of H3 histone (H3K56) (Stewart et al., 2005; Tao et al., 2013). Although we did not detect H3K9 or H3K56 acetylation, our results did indicate that SIRT6 reduced c-Myc expression. Therefore, combined with previous reports, we deduced that ERC-Exo treatment increased SIRT6 expression in naïve CD4^+^ T cells and then decreased the transcription and translation levels of c-Myc by promoting its deacetylation at the H3K9 or H3K56 sites, which consequently decreased the expression of key proteins ASCT2 and GLS1 for glutaminolysis.

ASCT2 is an important amino acid transporter for glutamine uptake into the cytoplasm, and GLS1 is the first rate-limiting enzyme for glutaminolysis after entering the cytoplasm (Liu et al., 2023). They control glutaminolytic uptake and enzymolysis, respectively. Previous studies have shown that glutaminolysis is closely related to the activation and differentiation of naïve CD4^+^ T cells (Yu et al., 2022; Nakaya et al., 2014). For example, Yu *et al.* revealed that inhibiting the activation of naïve CD4^+^ T cells and Th1/Th17 cell differentiation by blocking glutaminolysis play a therapeutic role in autoimmune hepatitis (Yu et al., 2022). Consistent with these reports, our study found that, even with the addition of CD3/CD28, SIRT6-expressing ERC-Exos inhibited naïve CD4^+^ T cell activation. Additionally, previous studies have shown that Th1/Th17 cell generation requires mTORC1 activation, whereas Tregs do not (Nakaya et al., 2014; Delgoffe et al., 2009). Nakaya *et al.* also found that after the T cell receptor stimulation by CD3/CD28, naïve CD4^+^ T cells begin to absorb large quantities of glutamine via ASCT2, thereby activating the downstream target mTORC1 and promoting the differentiation of naïve CD4^+^ T cells into Th1 and Th17 cells (Nakaya et al., 2014). However, Treg differentiation is not affected by glutamine intake. Subsequently, the knockout experiment confirmed that the deletion of ASCT2 affected the differentiation of Th1 and Th17 cells by reducing glutamine uptake but had no effect on Treg differentiation, indicating that Tregs can differentiate normally under glutamine deficiency (Nakaya et al., 2014). Similarly, our data showed that SIRT6-expressing ERC-Exos inhibited mTORC1 activation by weakening glutaminolysis, thus reducing the differentiation of Th1/Th17 cells and increasing that of Tregs.

mTORC1, a center for sensing and integrating various signals from the environment to control metabolism, plays an indispensable role in integrating the metabolic spectrum and guiding the differentiation of naïve CD4^+^ T cells (Huang et al., 2020). Previous studies have demonstrated that Ras homolog enriched in brain (Rheb) (an upstream activating protein of mTORC1)-deficient CD4^+^ T cells suppress Th1 cell differentiation by weakening the T cell response to IL-12 and preventing T-bet transcription (Delgoffe et al., 2011; Chornoguz et al., 2017). Regarding Th17 cells, the activated mTORC1 can increase the phosphorylation level of STAT3 at tyrosine 705 (Tyr705) in naïve CD4^+^ T cells, which is necessary for retinoic acid receptor-related orphan receptor gamma t (RORγt) expression (Wang et al., 2020). As a negative regulator of STAT3, activated mTORC1 also promotes STAT3 phosphorylation and upregulates RORγt expression by blocking suppressor of cytokine signaling 3 (SOCS3) (Wang et al., 2020). Then, activated mTORC1 upregulates HIF-1α expression to promote glycolysis in naïve CD4^+^ T cells, consequently promoting Th17 cell differentiation (Dang et al., 2011). Finally, activated mTORC1 enhances Th17 cell differentiation in a ribosomal S6 kinase 1/2 (S6K1/2)-dependent manner. Specifically, S6K1 inhibits the expression of Gfi1, a negative regulator of Th17 cell differentiation, and S6K2 promotes the nuclear localization of RORγt, thereby upregulating RORγt expression (Kurebayashi et al., 2012). However, the function of mTORC1 is reversed during Treg differentiation. For example, activated mTORC1 can block Treg generation by preventing Smad3 phosphorylation or H3K4 methylation close to the Foxp3 transcription start site, both of which promote Foxp3 transcription (Kurebayashi et al., 2012). Moreover, mTORC1 improves glycolytic activity by inducing HIF-1α expression, but Treg differentiation does not rely on glycolytic metabolism to provide energy compared to Th17 cells, resulting in a significant difference in the differentiation of Th17 cells and Tregs (Dang et al., 2011; Shi et al., 2011). In summary, these reports showed the different roles of mTORC1 in CD4^+^ T cell differentiation; that is, activated mTORC1 promotes the pro-inflammatory cell differentiation, such as Th1/Th17 cells, while inactivated mTORC1 promotes the differentiation of anti-inflammatory cells, such as Tregs.

Amino acids, especially glutamine, leucine, arginine, and methionine, play an important role in the activation of the mTORC1 signaling pathway (Jewell et al., 2015; Meng et al., 2020; Gu et al., 2017). ASCT2, an essential amino acid transporter for Glutamine uptake, regulates the mTORC1 activation status (Nakaya et al., 2014). Nakaya *et al.* demonstrated that ASCT2 mediates TCR-stimulated mTORC1 activation in naïve CD4^+^ T cells (Nakaya et al., 2014). As mentioned above, our results showed that the expression of ASCT2 in naïve CD4^+^ T cells was downregulated after administration of ERC-Exos. Subsequently, we further detected that the glutamine uptake efficiency and α-KG content in these T cells also decrease. Therefore, we further explored mTORC1 activity, and our findings were consistent with those of previous studies showing that mTORC1 is inactivated when ASCT2 is downregulated in naïve CD4^+^ T cells (Nakaya et al., 2014; Huang et al., 2020). α-KG, the metabolic product of glutamine, promotes mTORC1 assembly and positively regulates the mTORC1 pathway when present in lysosomes (Cabré et al., 2021; Durán et al., 2013). In addition to this direct effect, glutaminolysis also regulates the activation of mTORC1 by affecting energy generation in cells. In specific, glutaminolysis metabolic products such as α-KG can participate in tricarboxylic acid cycle, which is the main pathway to produce energy (Cabré et al., 2021). Excessive energy production in cells inhibits adenosine 5′-monophosphate (AMP)-activated protein kinase (AMPK), an upstream negative regulatory target of mTORC1 (González et al., 2020). Hence, combining these reports with our results, we speculate that α-KG inactivates mTORC1 by reducing energy generation in naïve CD4^+^ T cells to activate AMPK or directly affecting mTORC1 assembly. Although we were not clear about how α-KG inactivates mTORC1 in CD4^+^ T cells, our results showed that SIRT6-expressing ERC-Exos could indeed inactivate mTORC1 by reducing α-KG, a key metabolite of glutamine decomposition.

Previously, studies have revealed that CD4^+^ effector T cell subsets participate in allograft rejection (Wa et al., 2011). Th1 and Th17 cells are closely related to the occurrence of transplant rejection, while Tregs are often related to the formation and maintenance of immune tolerance (Wa et al., 2011). When CD4^+^ T cells are activated, they can produce IL-2, which can be used as a growth factor to induce the proliferation of Th1 cells expressing transcription factor T-bet expressed in T cells (Hall, 2015). And Th1 cells can become an important part of allograft rejection by secreting IFN-γ. On the one hand, when Th1 cells are recruited to inflammatory tissues, they can directly mediate tissue damage by releasing IFN-γ (Hall, 2015). On the other hand, IFN-γ can also be used as a bridge to connect Th1 cells and induce other anti-inflammatory cell activation, such as M1 macrophages (Hall et al., 1984). In addition, Th17 cells also play an important role in tissue injury on AR. IL-17A, a pro-inflammatory cytokine, can be produced by CD8^+^ memory T cells, eosinophils, neutrophils, and monocytes, but its main secretory cell is still Th17 cells (Afzali et al., 2007). IL-17A can not only activate the inflammatory signal pathway (such as the mitogen-activated protein kinase (MAPK) and NF-κB pathways) of target cells by binding to its receptors but also induce further inflammatory response by recruiting neutrophils (Laan et al., 2001; Miyamoto et al., 2003). Besides, Li *et al.* also reported that the antagonism of IL-17A signaling pathway could reduce the IFN-γ production in the graft and prolong the graft survival (Li et al., 2006). As for Tregs, Tregs are one of the important factors to maintain immune tolerance, which inhibits excessive T cell activation and proliferation by secreting IL-10 (Afzali et al., 2007; Edemir et al., 2008). Specifically, IL-10 inhibits the response of antigen-specific effector cells by inhibiting the production of pro-inflammatory cytokines (Edemir et al., 2008). Studies had demonstrated that the upregulation of IL-10 gene could improve renal function and prolong the survival of renal allografts in a rat transplantation model (Chen et al., 2007). Our results are consistent with these previous reports. In our mouse transplantation model, IFN-γ and IL-17A showed high levels in serum and grafts, while IL-10 levels were relatively low in serum. After ERC-Exo treatment, the proinflammatory cytokines IFN-γ and IL-17 decreased in serum and grafts, but the level of IL-10 increased in serum. And this effect is obviously weakened with the knockdown of SIRT6 in ERC-Exos. Due to the massive secretion of these cytokines, the therapeutic effect of ERC-Exos on AR can be explained not only by CD4^+^ T cells but also by other immune cells, which is also worth our further study.

In conclusion, we confirmed the therapeutic effects of targeting T cell glutaminolysis in allogeneic transplantation using ERC-Exo therapy. By inhibiting the c-Myc-dependent glutaminolysis of naïve CD4^+^ T cells to remodel their differentiation, ERC-Exos can specifically alleviate acute transplant rejection, and this effect is mediated by SIRT6 in exosomes.

## Conclusion

The present study affirmed the therapeutic effect of ERC-Exos in alleviating AR. Moreover, we emphasized the crucial role of SIRT6 in ERC-Exo-mediated immune tolerance formation via remodeling inordinate CD4^+^ T differentiation. In a word, this study paves a way for the theoretical basis of the immunoregulatory potential of ERC-Exos and will guide the future clinical application of ERC-Exos in the treatment of AR.

## Data Availability

The raw data supporting the conclusions of this article will be made available by the authors, without undue reservation.
