# Supplementary figures and images for: Sirtuin 6 mediates the therapeutic effect of endometrial regenerative cell-derived exosomes in alleviation of acute transplant rejection by weakening c-myc-dependent glutaminolysis
Source: Front Cell Dev Biol. 2025 Sep 18;13:1564382. doi: 10.3389/fcell.2025.1564382 (PMC12488686; doi:10.3389/fcell.2025.1564382)

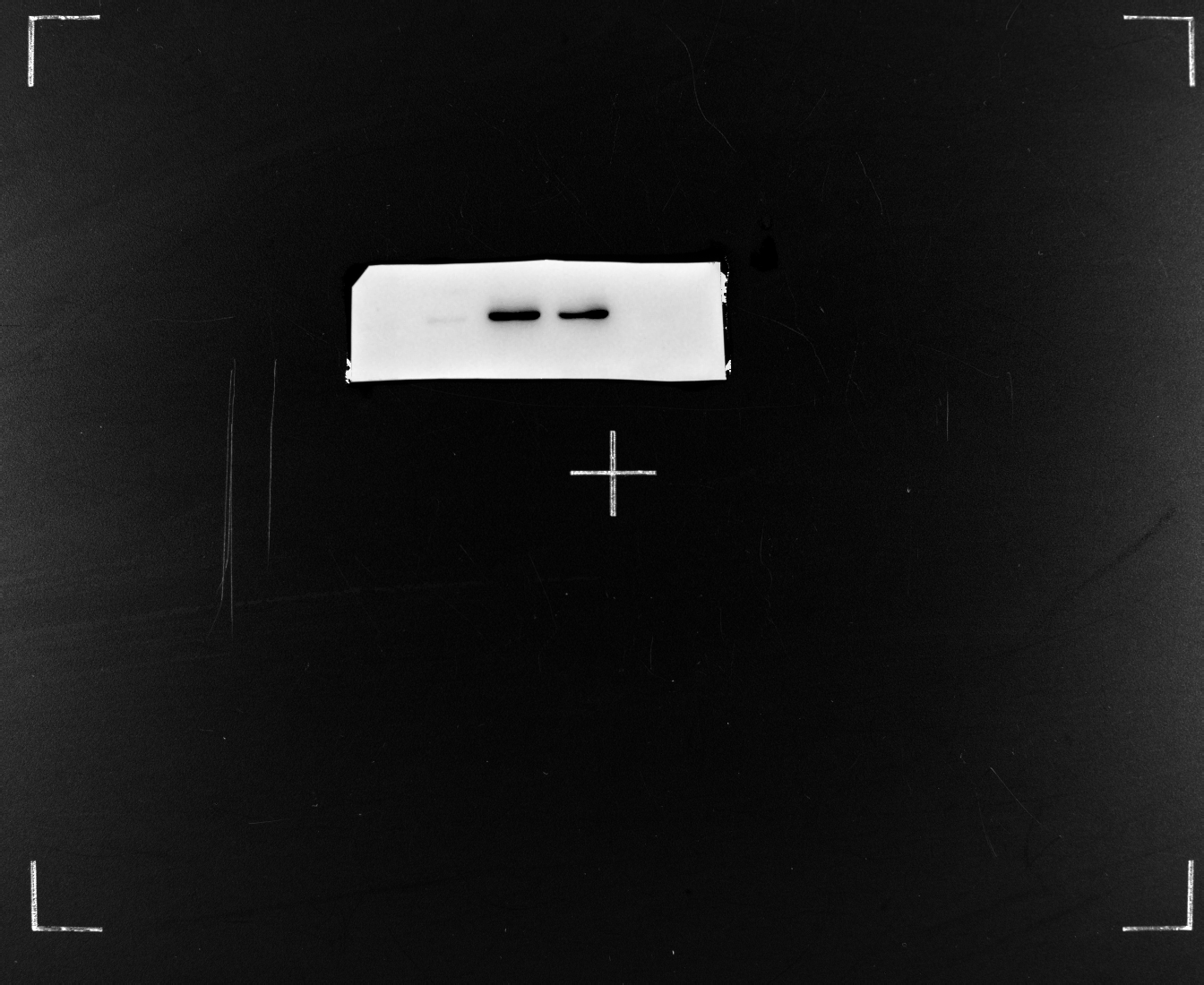

Supplement: Supplementary file 1 [file DataSheet3.zip › Fig. 2/0-merger.tif]

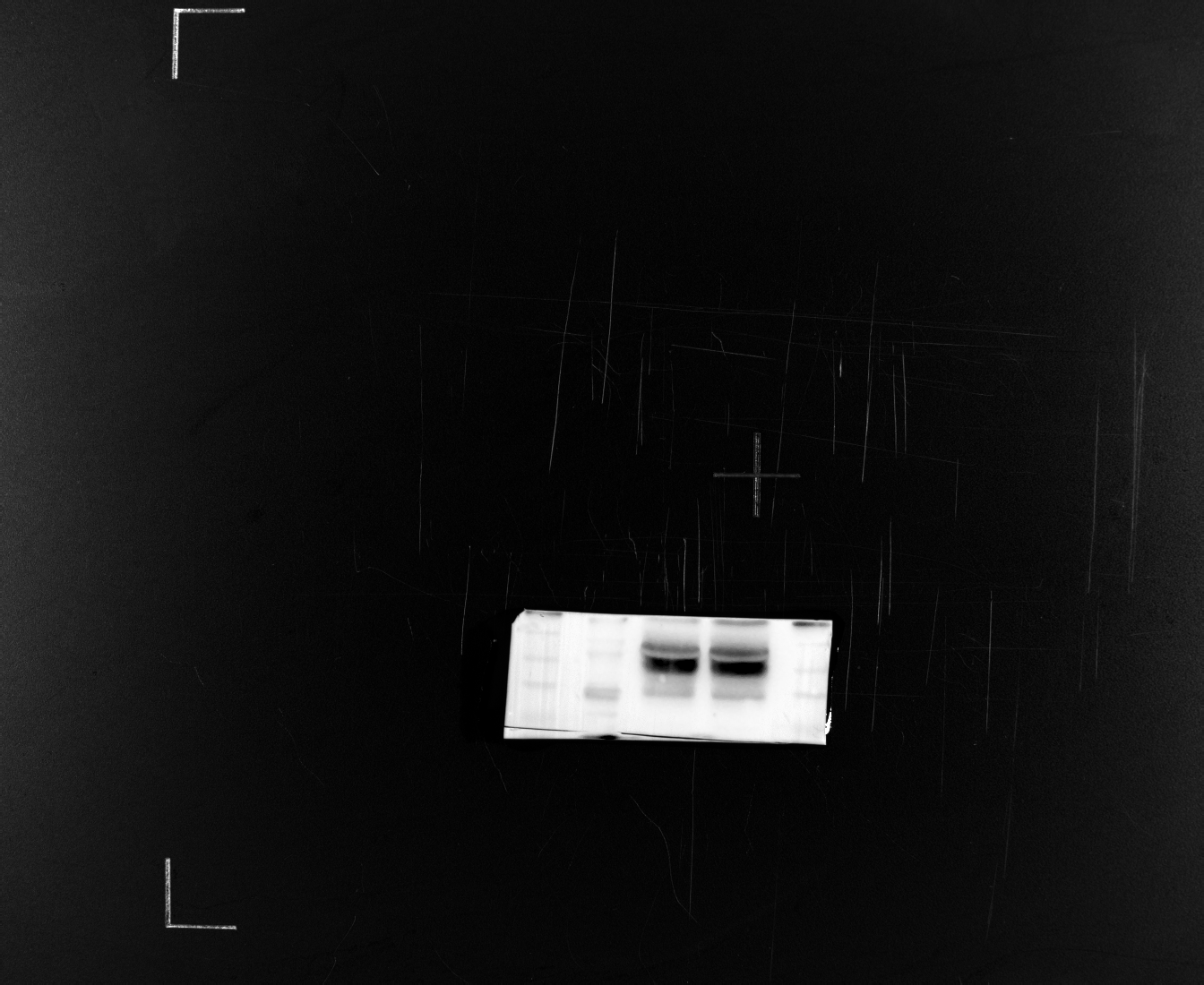

Supplement: Supplementary file 1 [file DataSheet3.zip › Fig. 2/00-merger.tif]

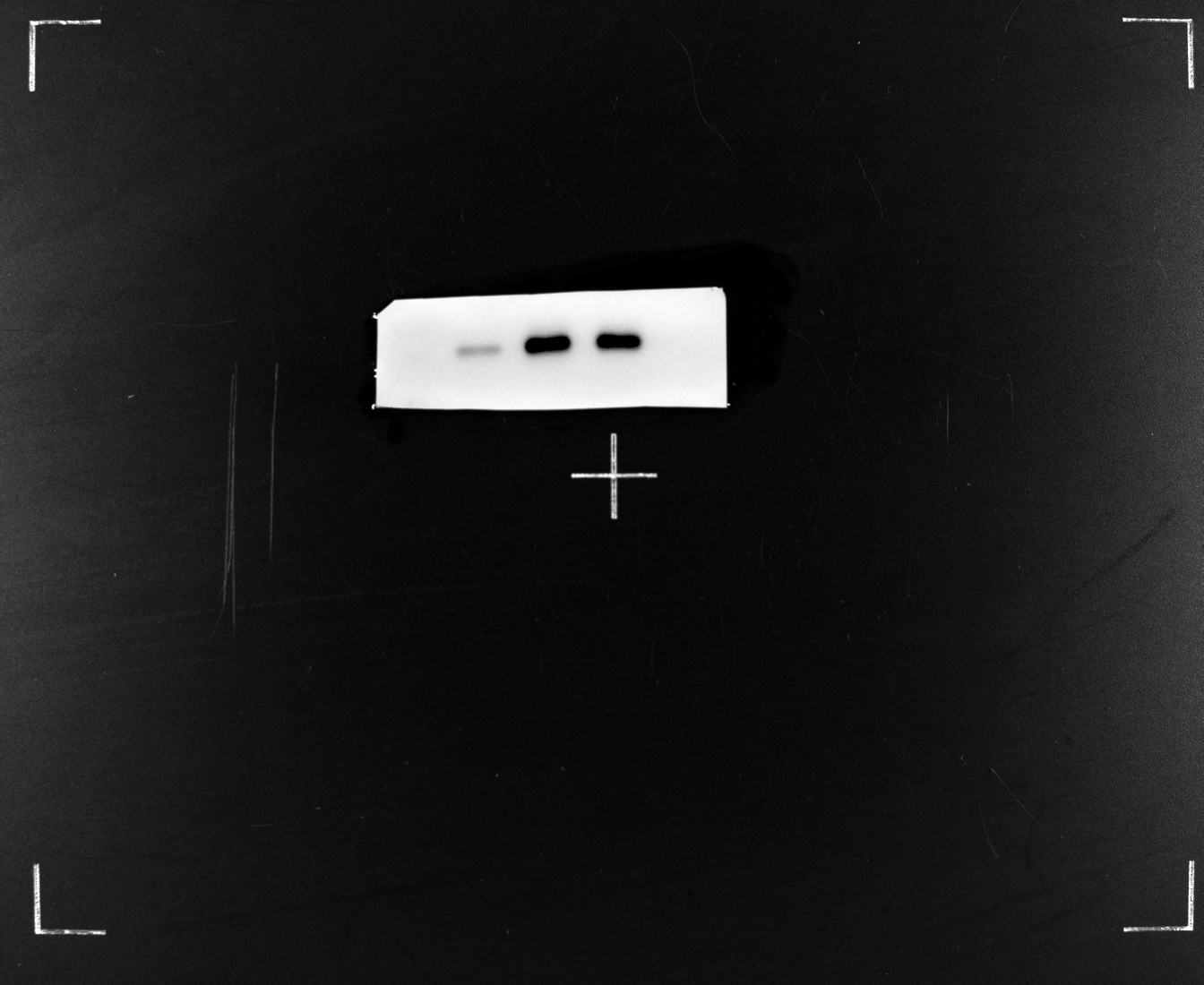

Supplement: Supplementary file 1 [file DataSheet3.zip › Fig. 2/000-merger.tif]

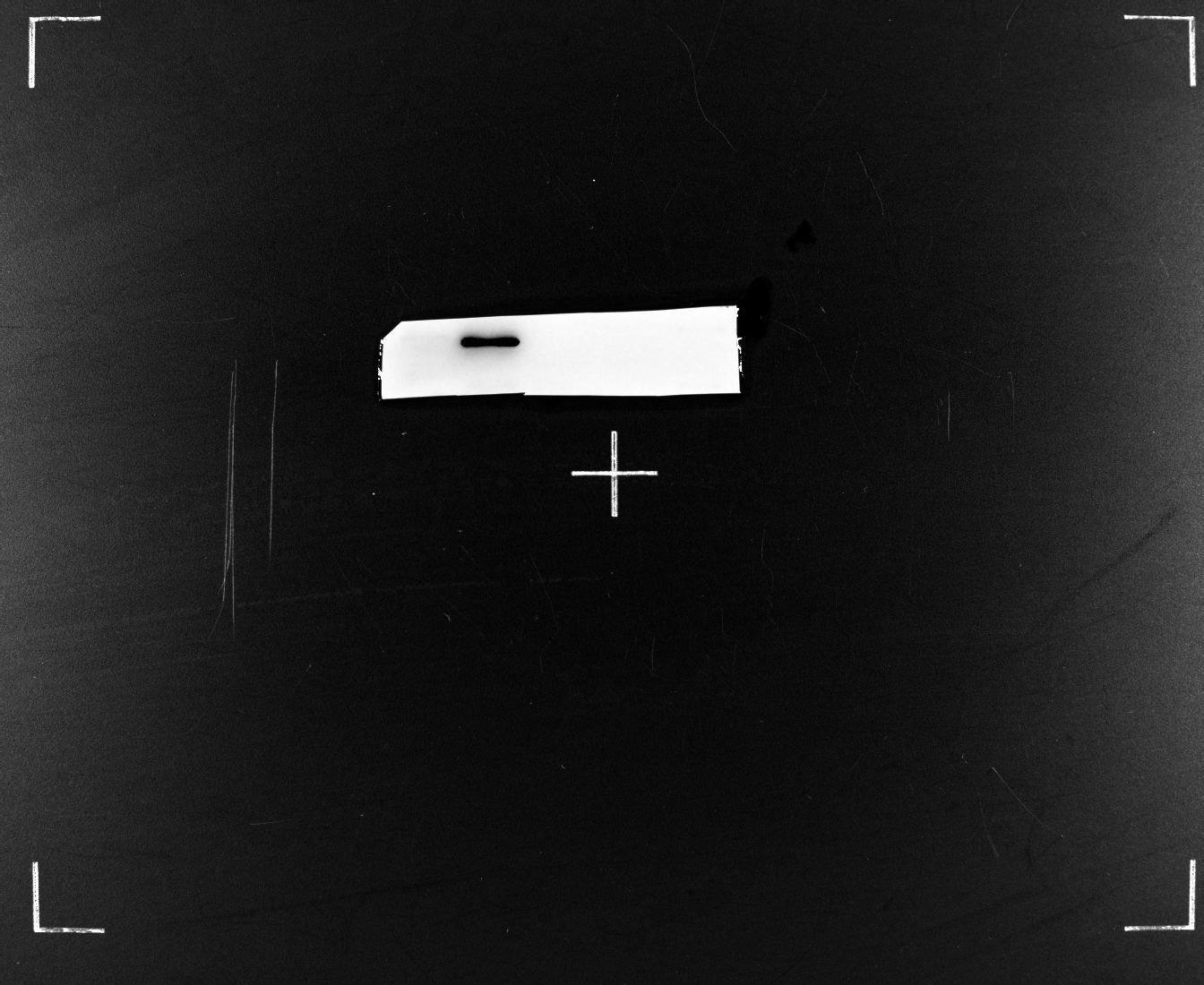

Supplement: Supplementary file 1 [file DataSheet3.zip › Fig. 2/0000-merger.tif]

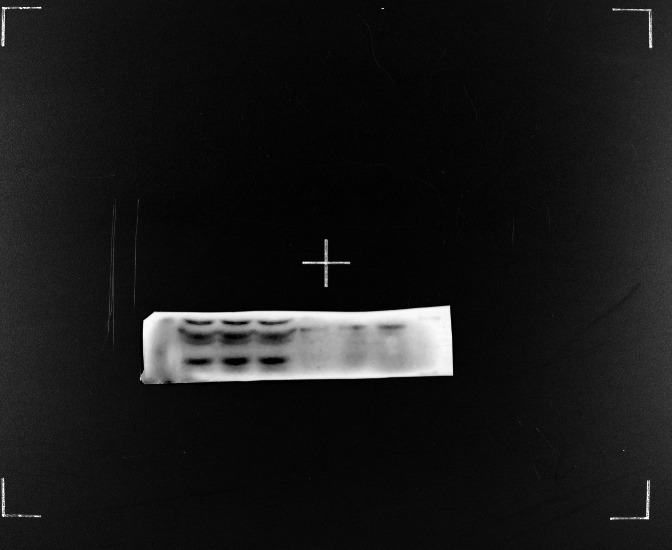

Supplement: Supplementary file 1 [file DataSheet3.zip › Fig. 2/001-merger.tif]

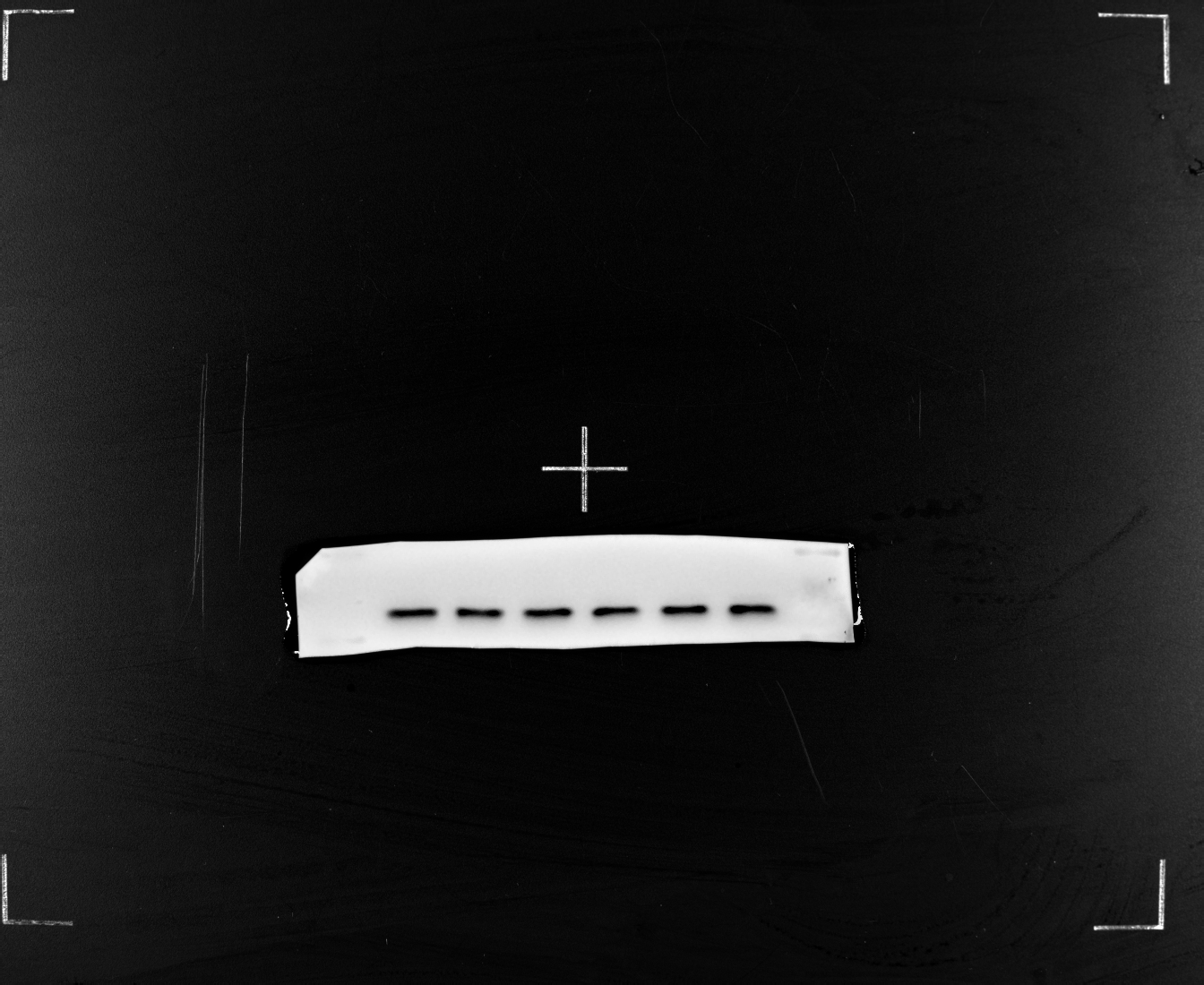

Supplement: Supplementary file 1 [file DataSheet3.zip › Fig. 2/002-merger.tif]

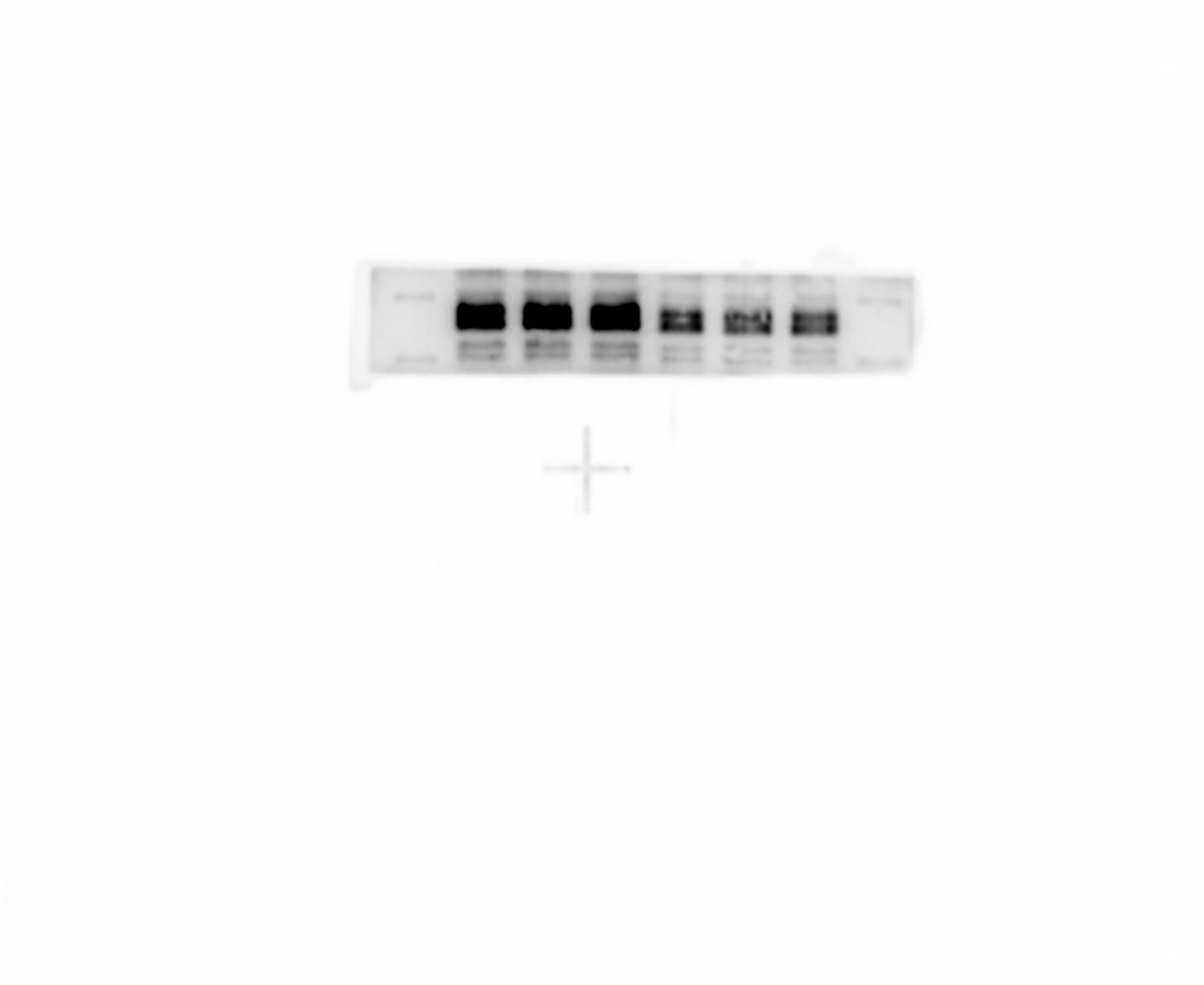

Supplement: Supplementary file 1 [file DataSheet3.zip › Fig. 2/003-luminescence[s2].tif]

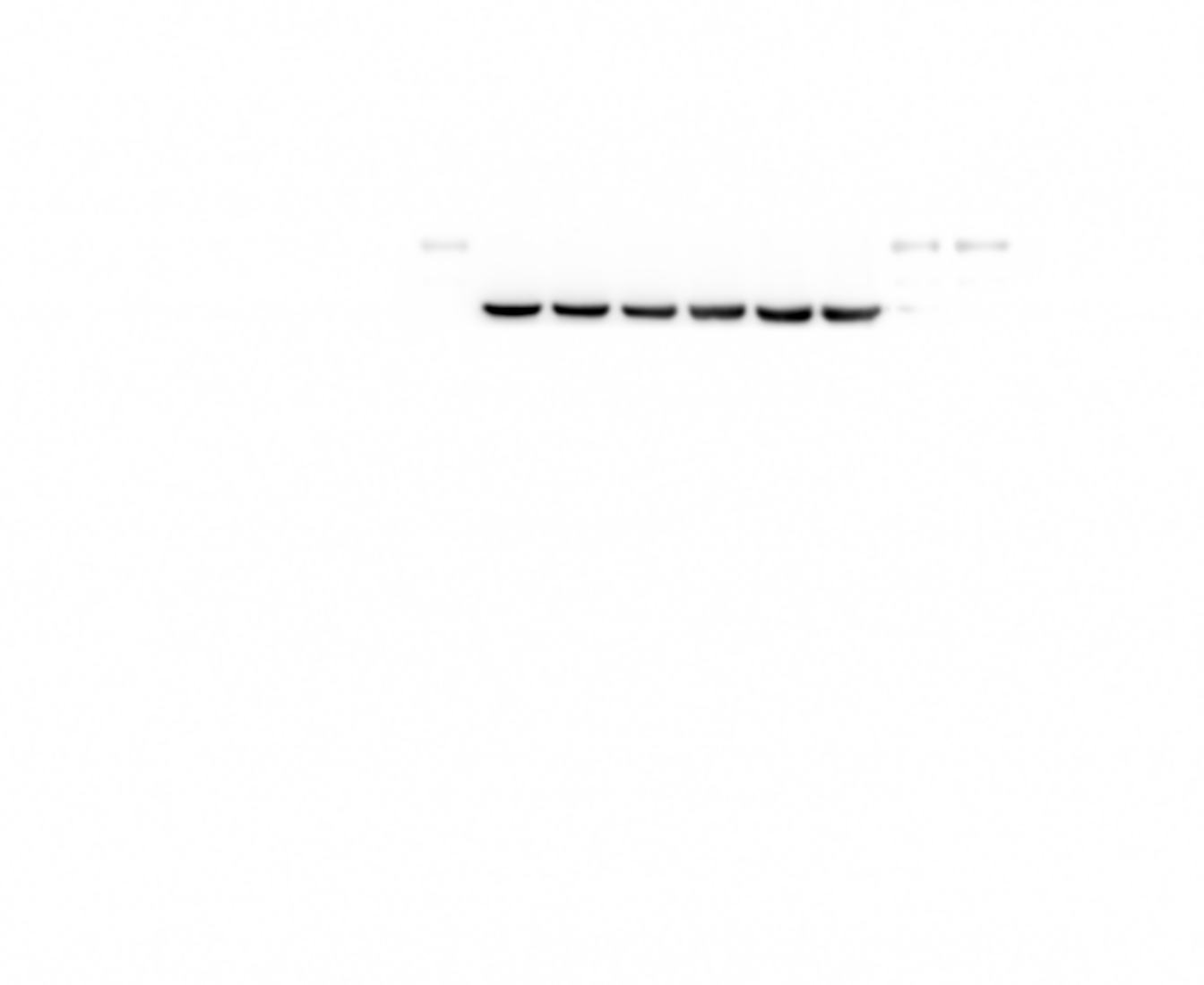

Supplement: Supplementary file 1 [file DataSheet3.zip › Fig. 2/005-luminescence[beta-sirt6-2].tif]

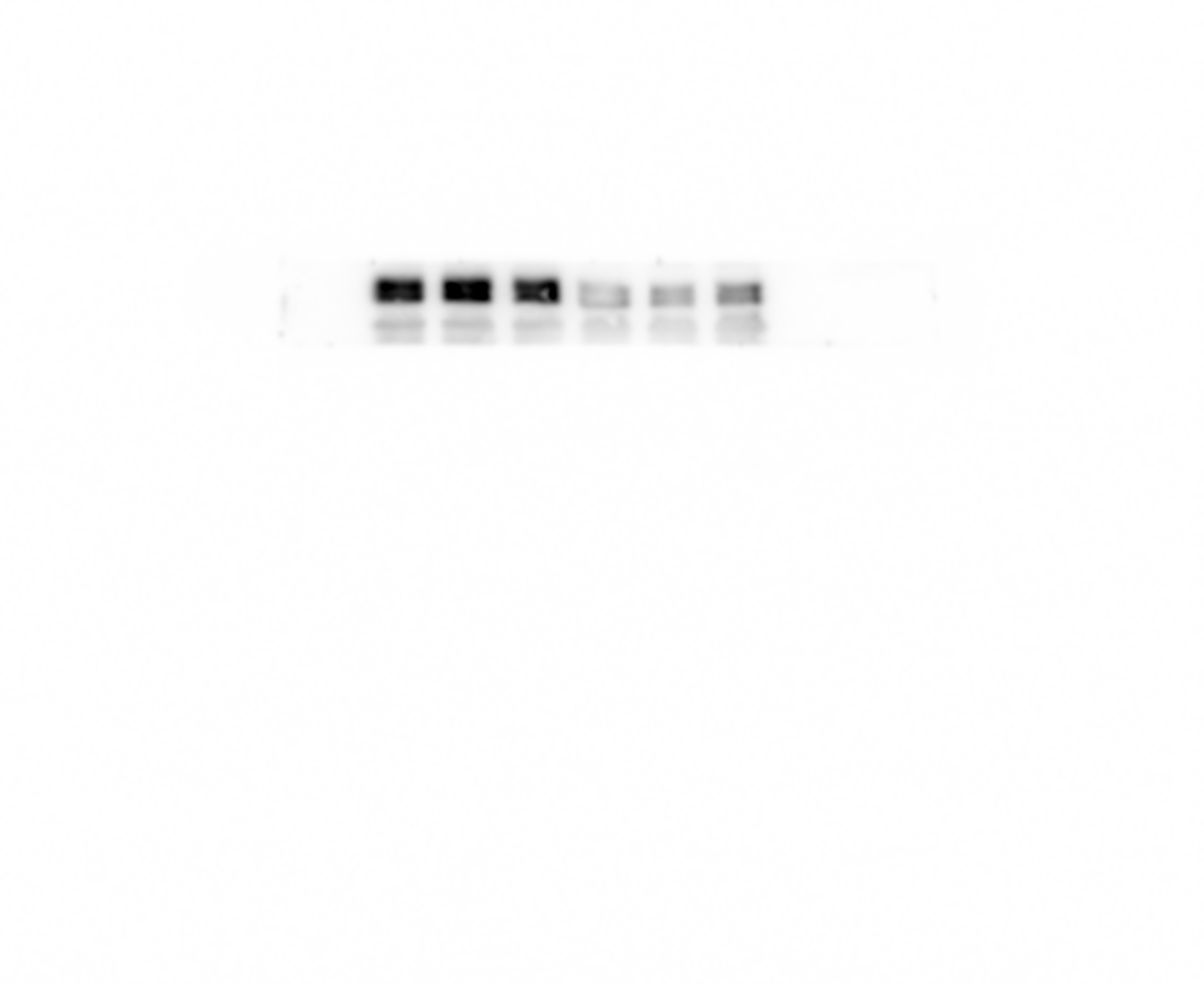

Supplement: Supplementary file 1 [file DataSheet3.zip › Fig. 2/011-luminescence[sirt6].tif]

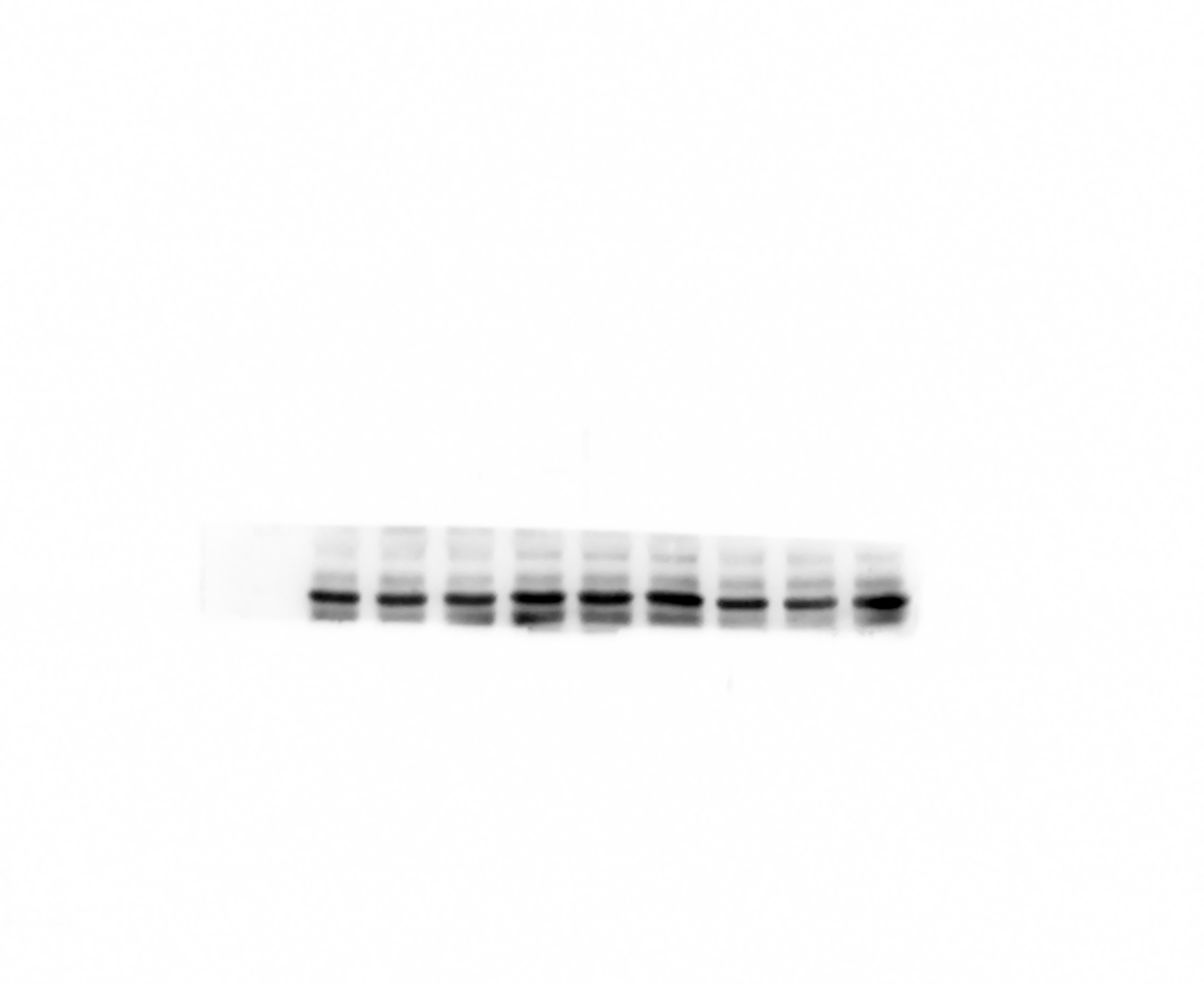

Supplement: Supplementary file 3 [file DataSheet4.zip › 001-luminescence[S T].tif]

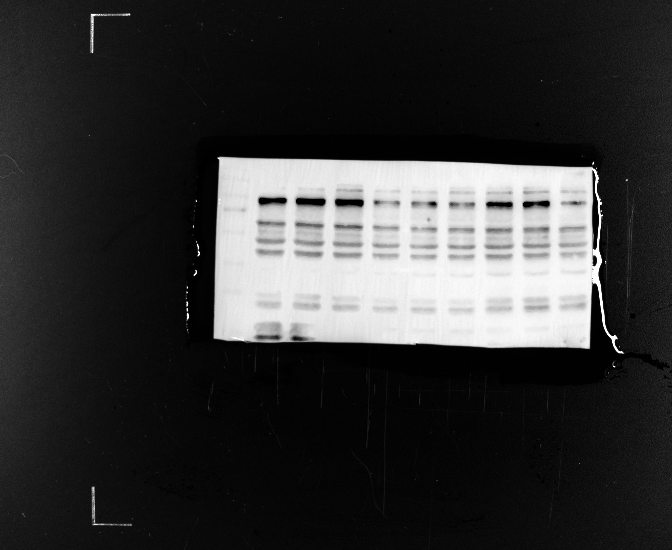

Supplement: Supplementary file 3 [file DataSheet4.zip › 001-merger [pS6].tif]

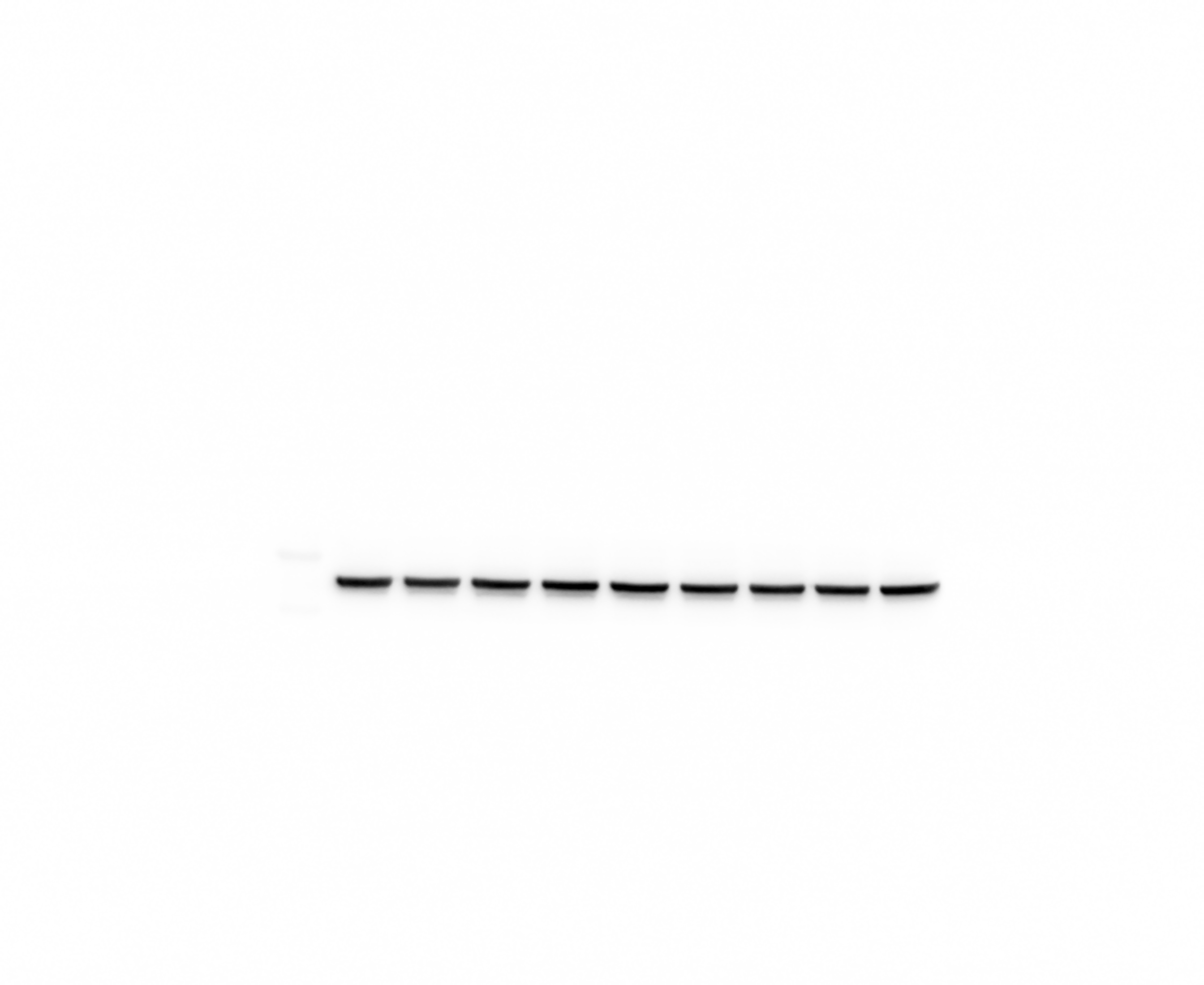

Supplement: Supplementary file 3 [file DataSheet4.zip › 002-luminescence[b].tif]

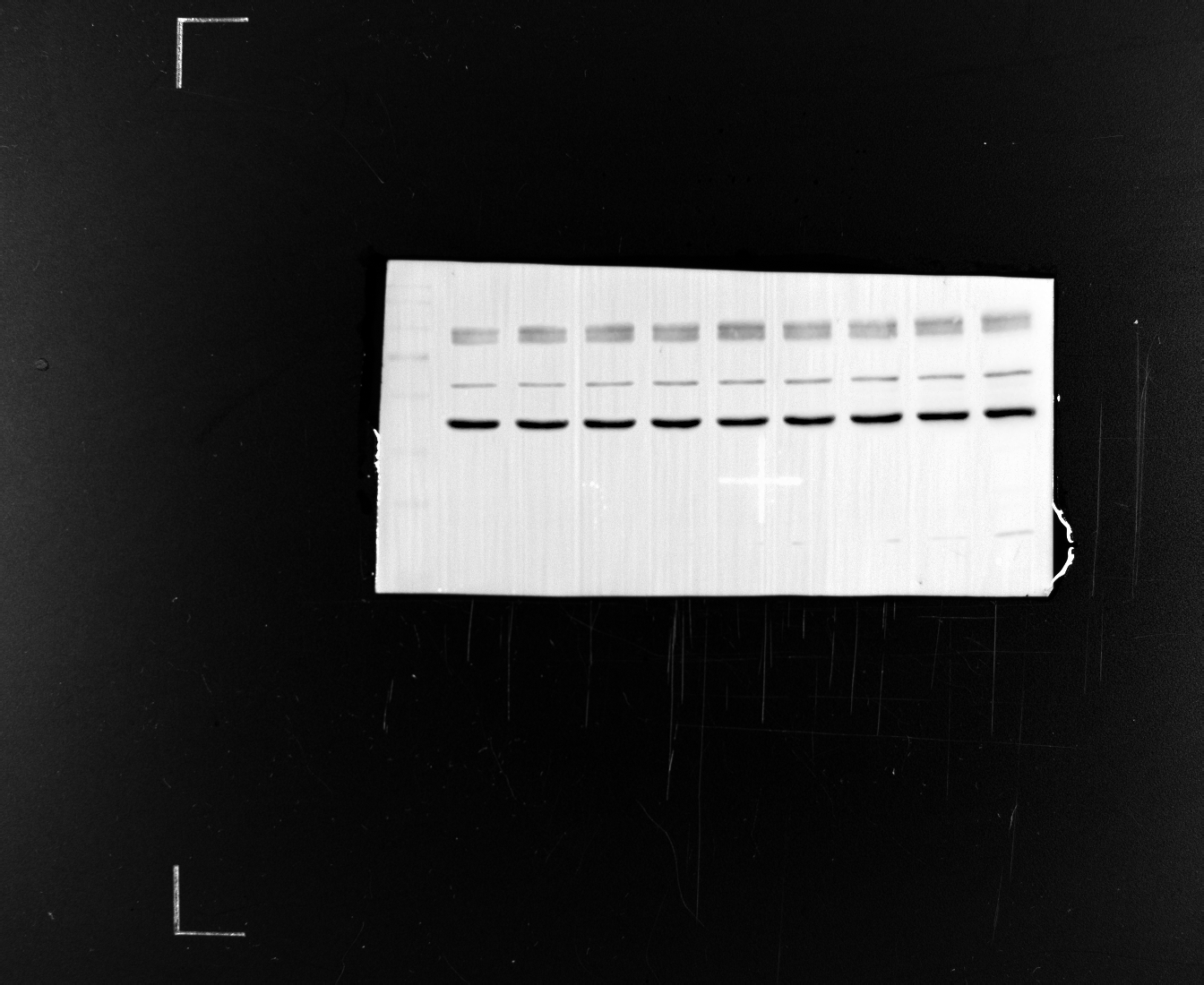

Supplement: Supplementary file 3 [file DataSheet4.zip › 003-merger [S6K].tif]

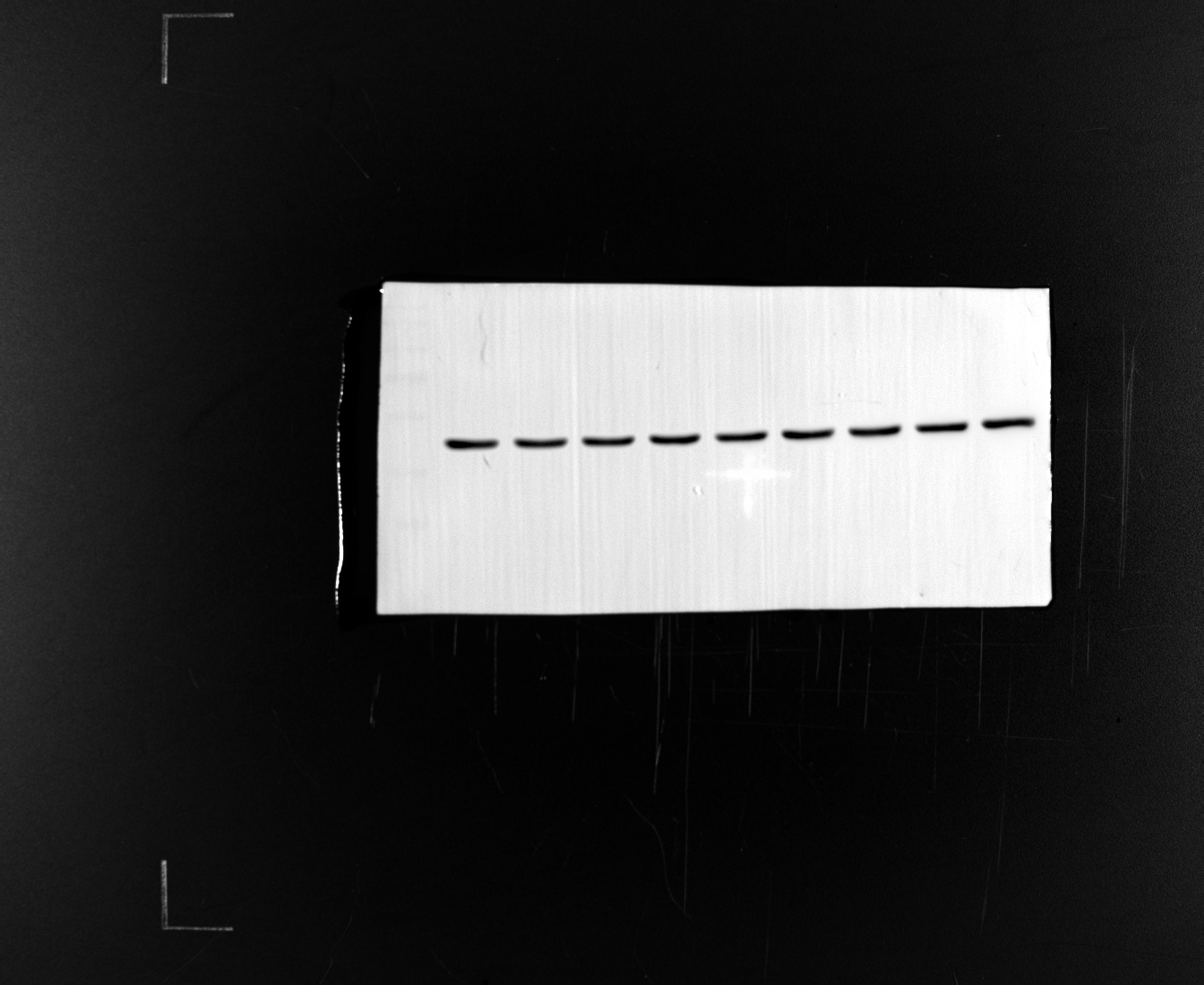

Supplement: Supplementary file 3 [file DataSheet4.zip › 401-merger [B].tif]

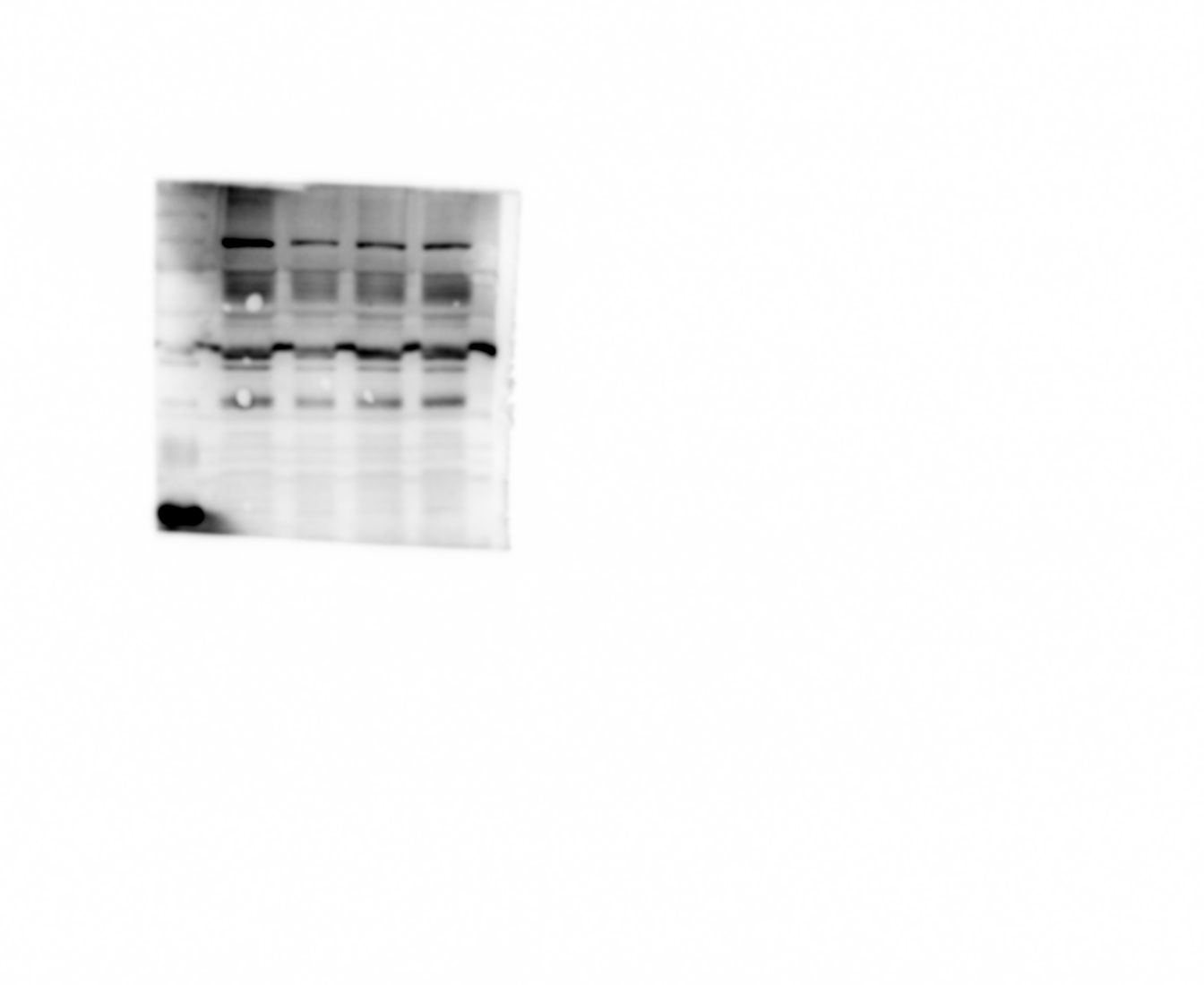

Supplement: Supplementary file 4 [file DataSheet1.zip › 001-luminescence[a2-2].tif]

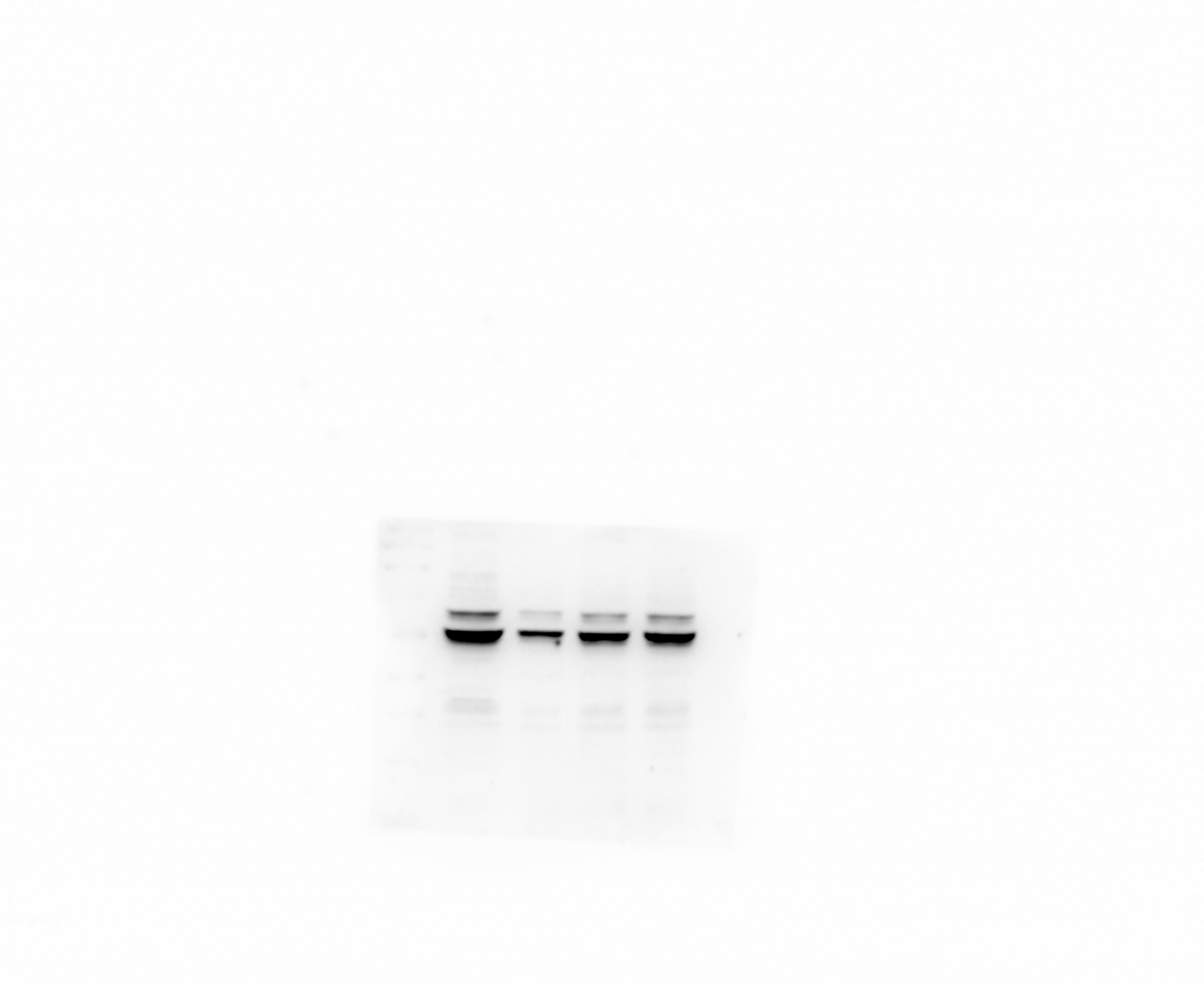

Supplement: Supplementary file 4 [file DataSheet1.zip › 001-luminescence[PSSS].tif]

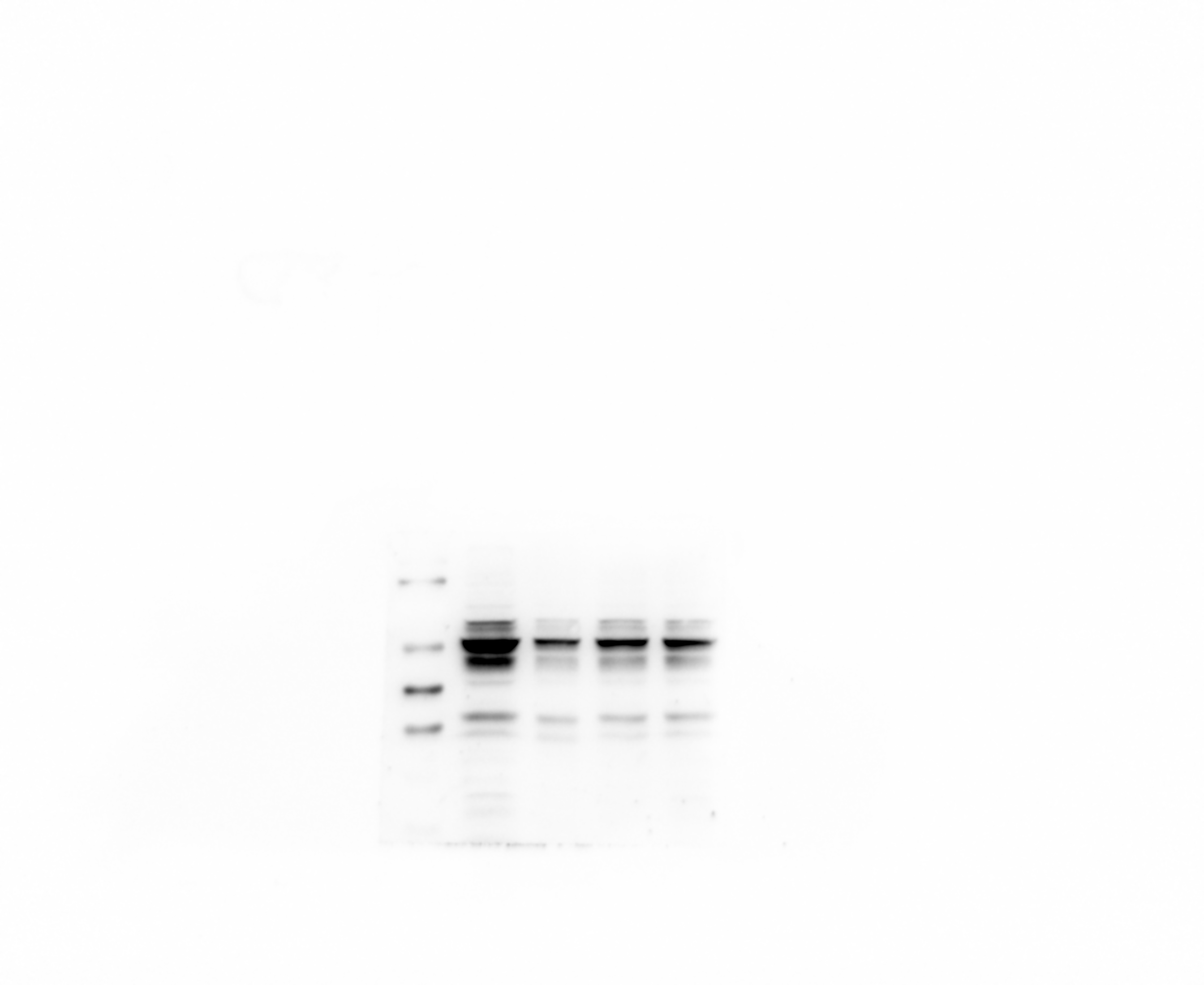

Supplement: Supplementary file 4 [file DataSheet1.zip › 003-luminescence[MYC2].tif]

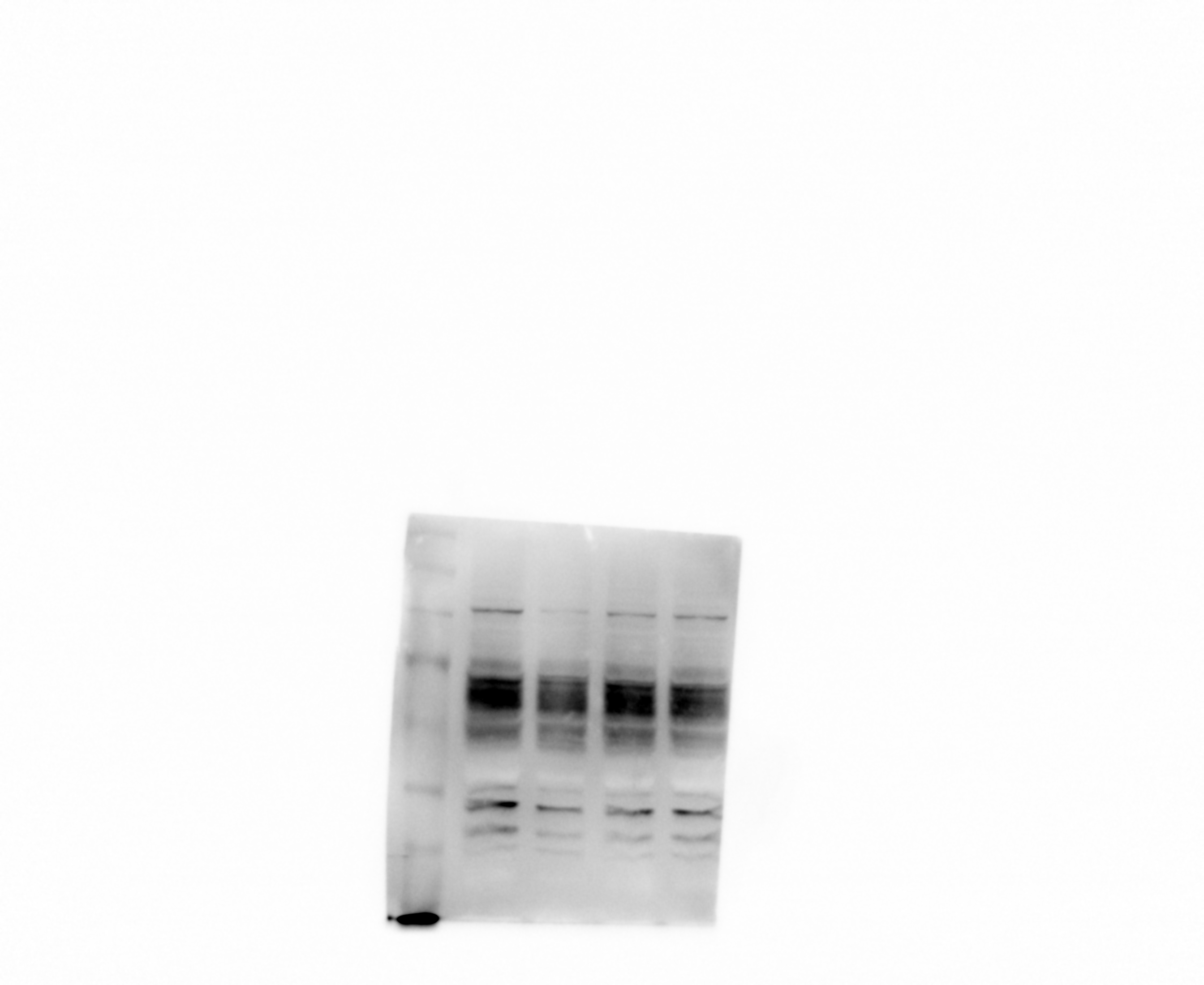

Supplement: Supplementary file 4 [file DataSheet1.zip › 004-luminescence[A3].tif]

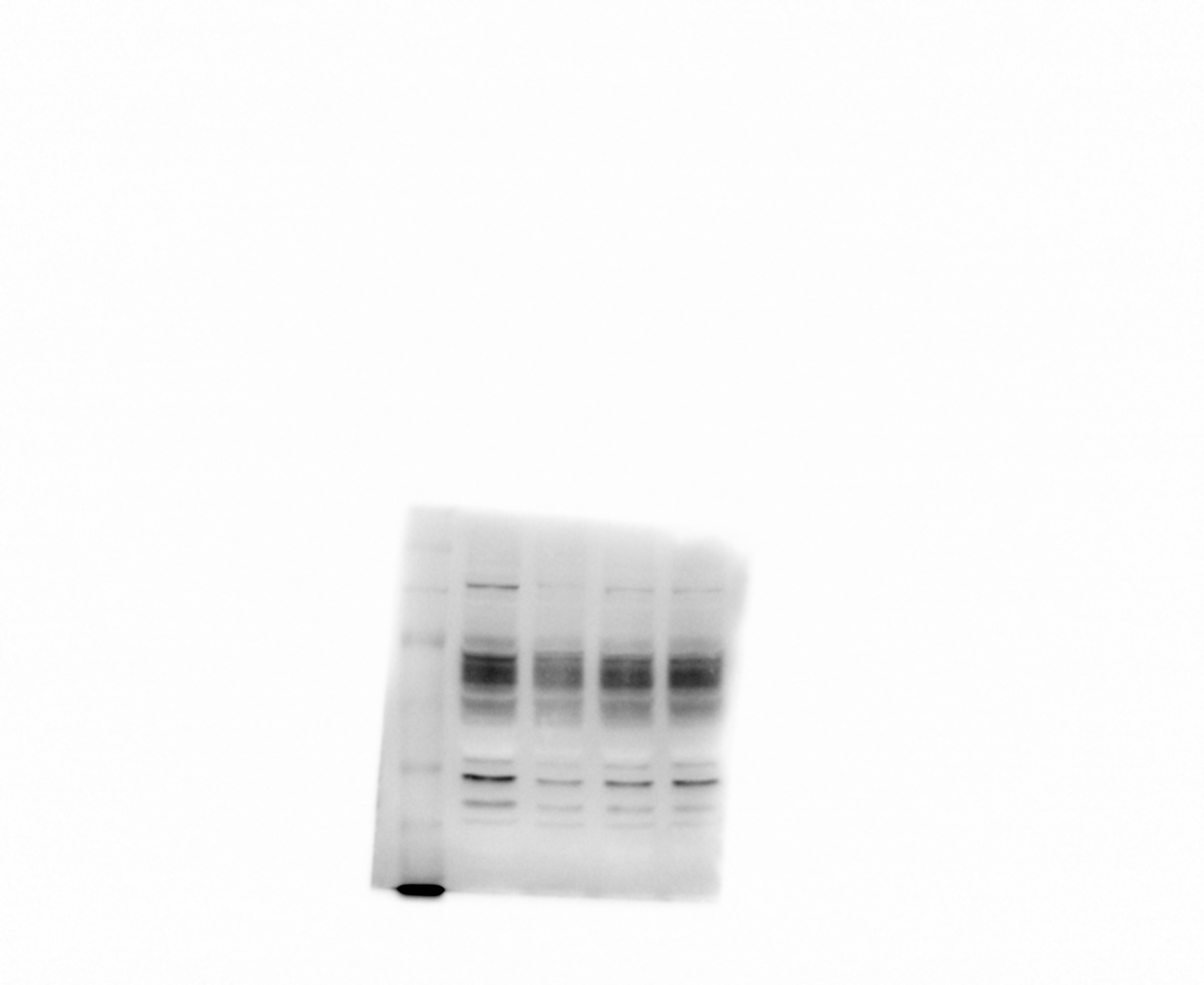

Supplement: Supplementary file 4 [file DataSheet1.zip › 005-luminescence[A2].tif]

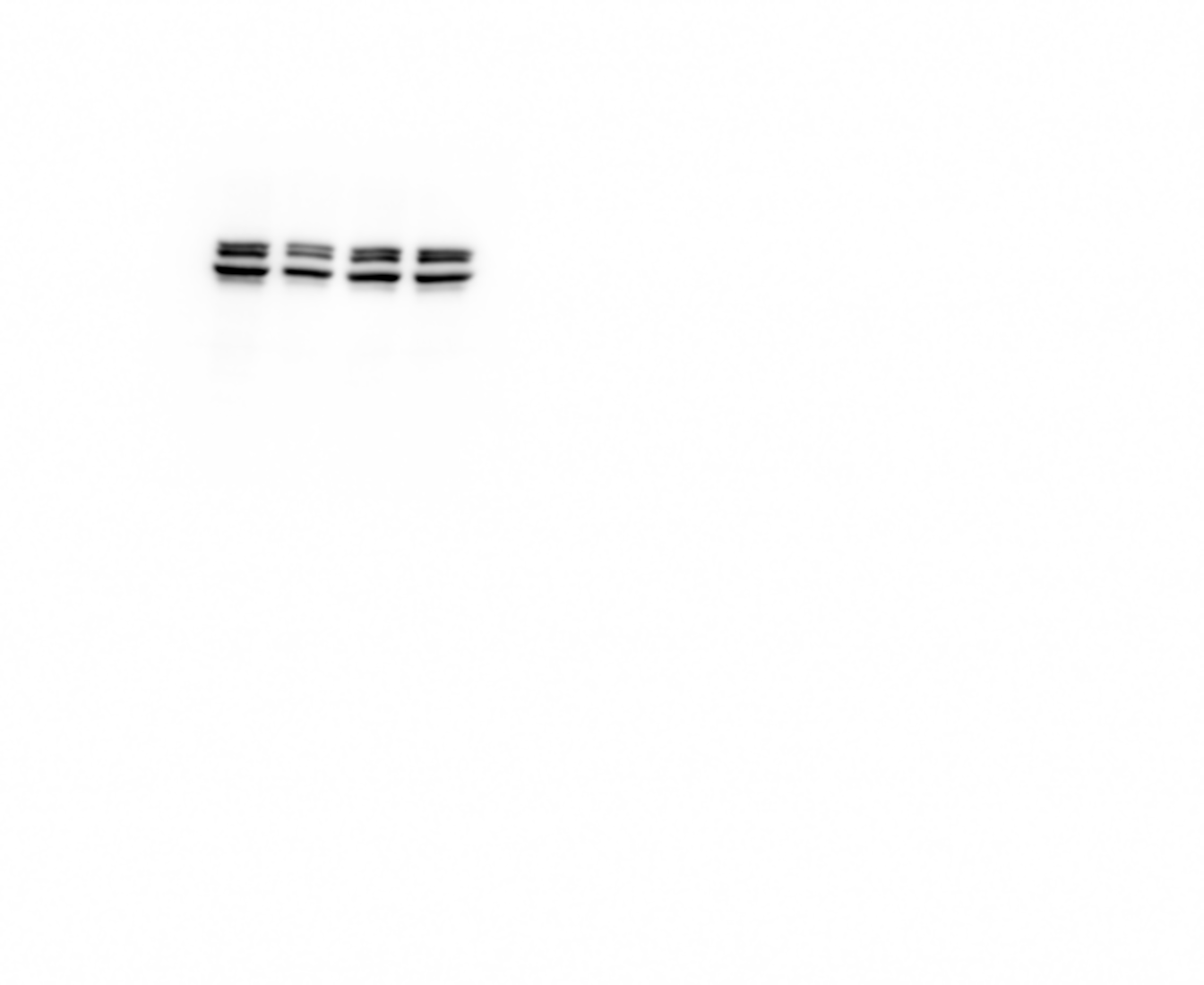

Supplement: Supplementary file 4 [file DataSheet1.zip › 005-luminescence[g2-2].tif]

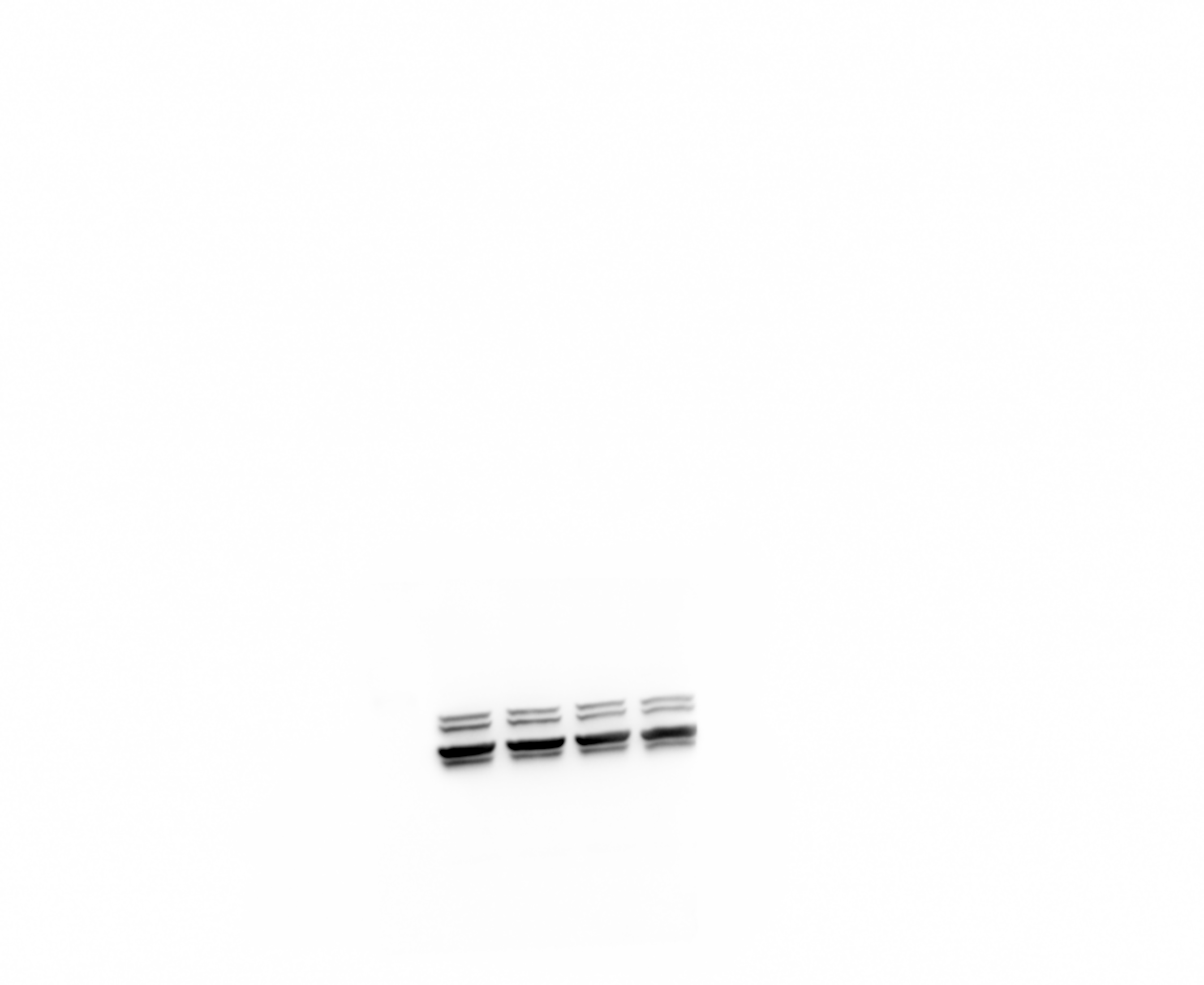

Supplement: Supplementary file 4 [file DataSheet1.zip › 005-luminescence[tub2].tif]

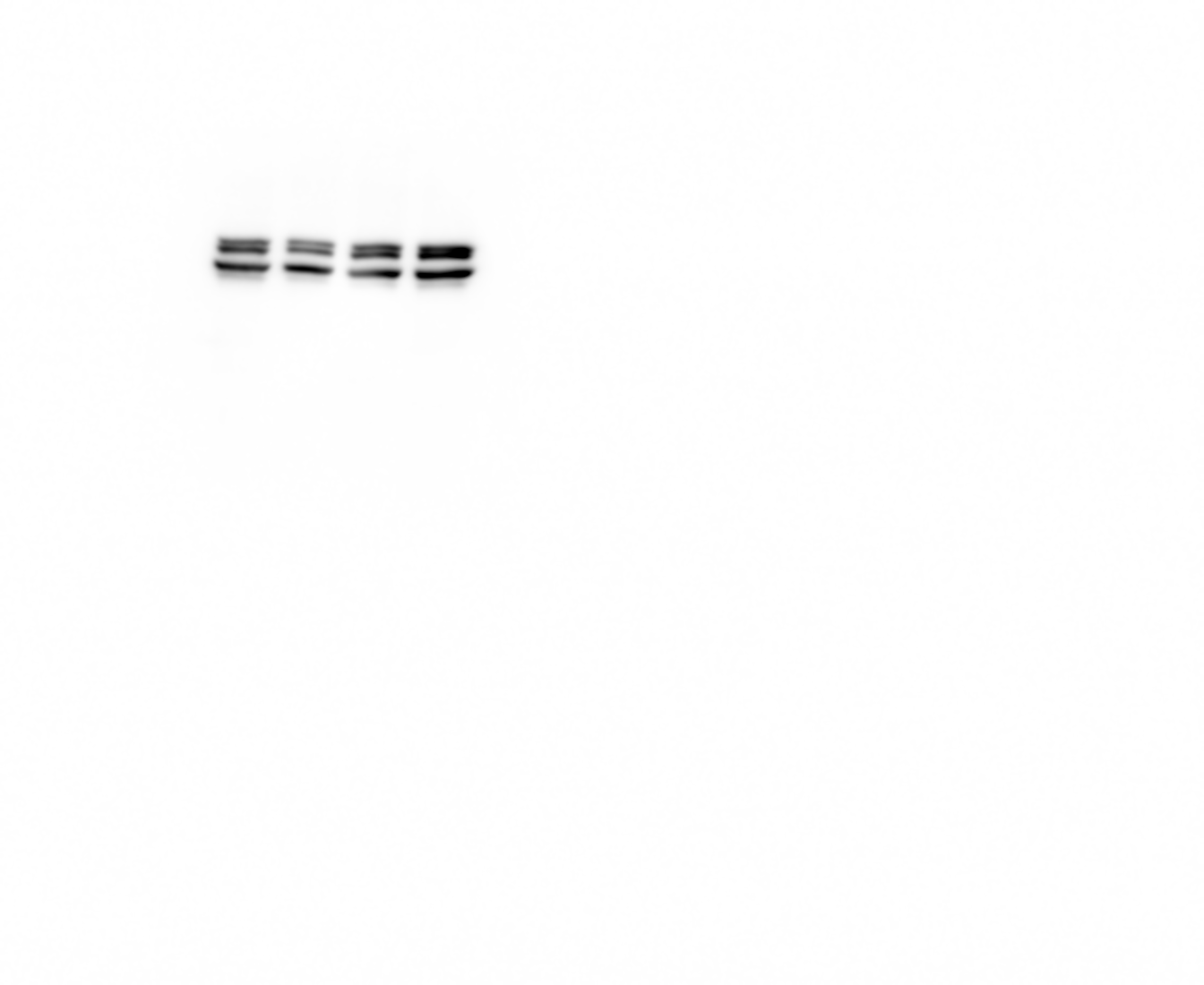

Supplement: Supplementary file 4 [file DataSheet1.zip › 006-luminescence[g2].tif]

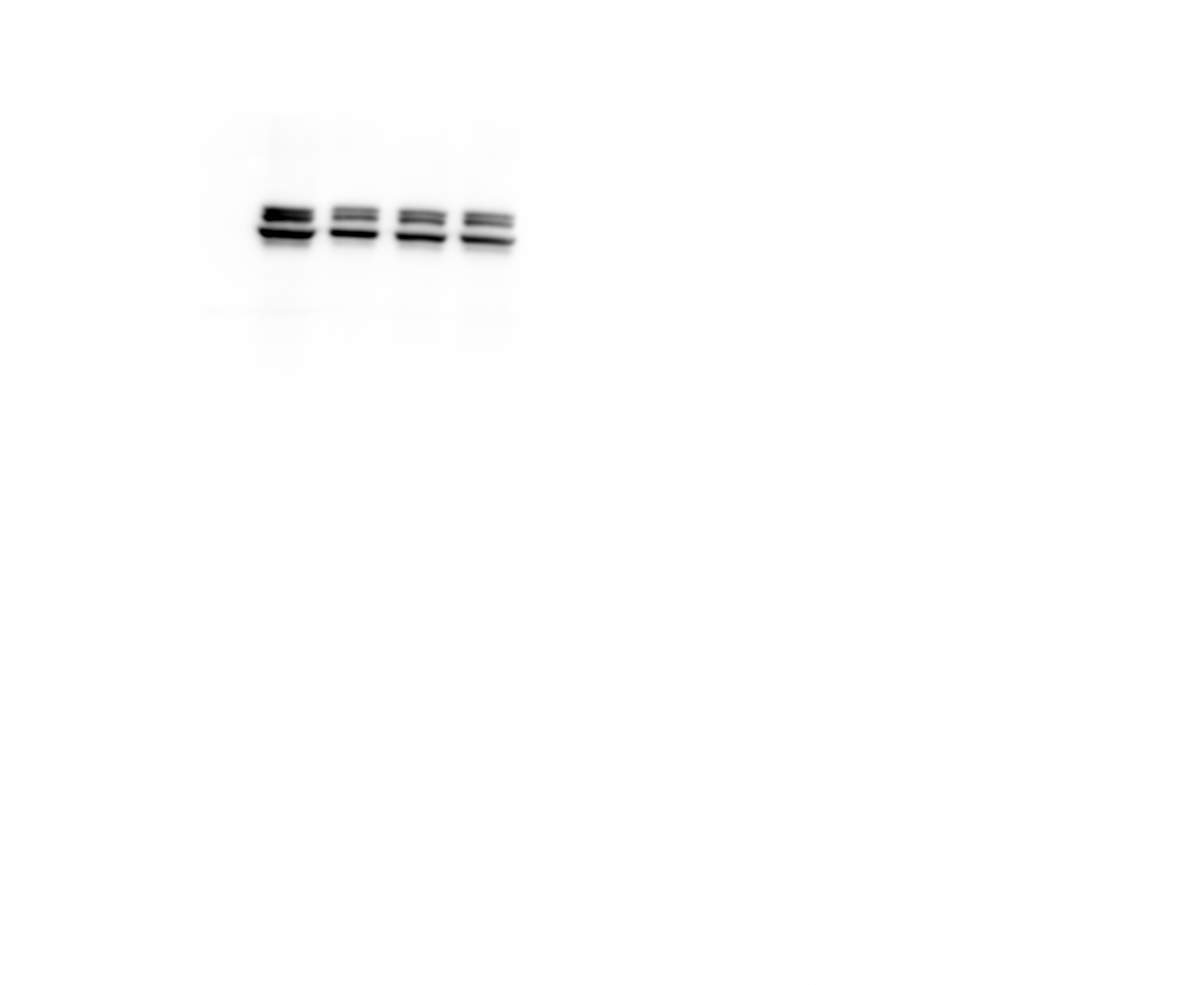

Supplement: Supplementary file 4 [file DataSheet1.zip › 007-luminescence[g1].tif]

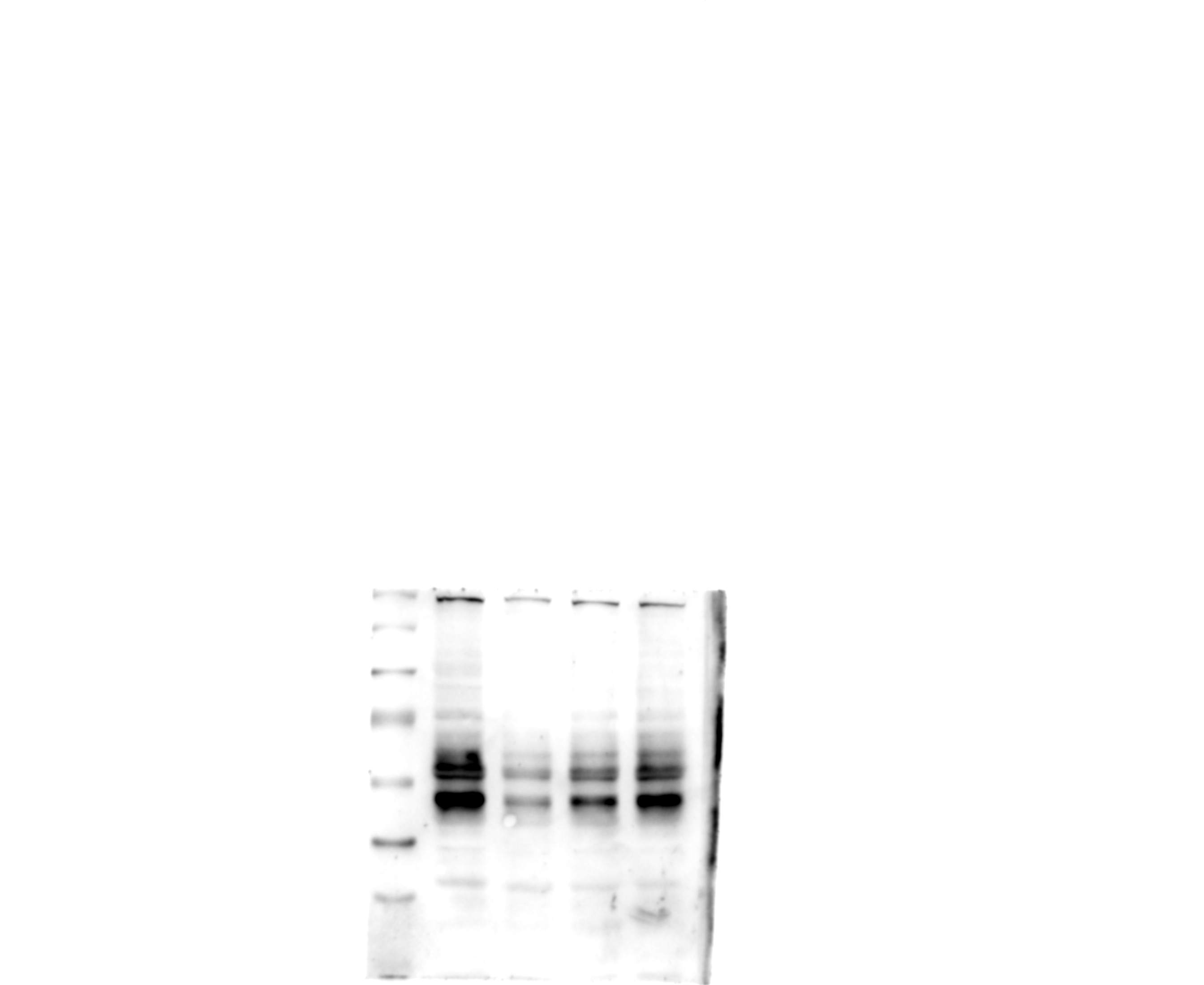

Supplement: Supplementary file 5 [file DataSheet2.zip › 001-luminescence[cmy22].tif]

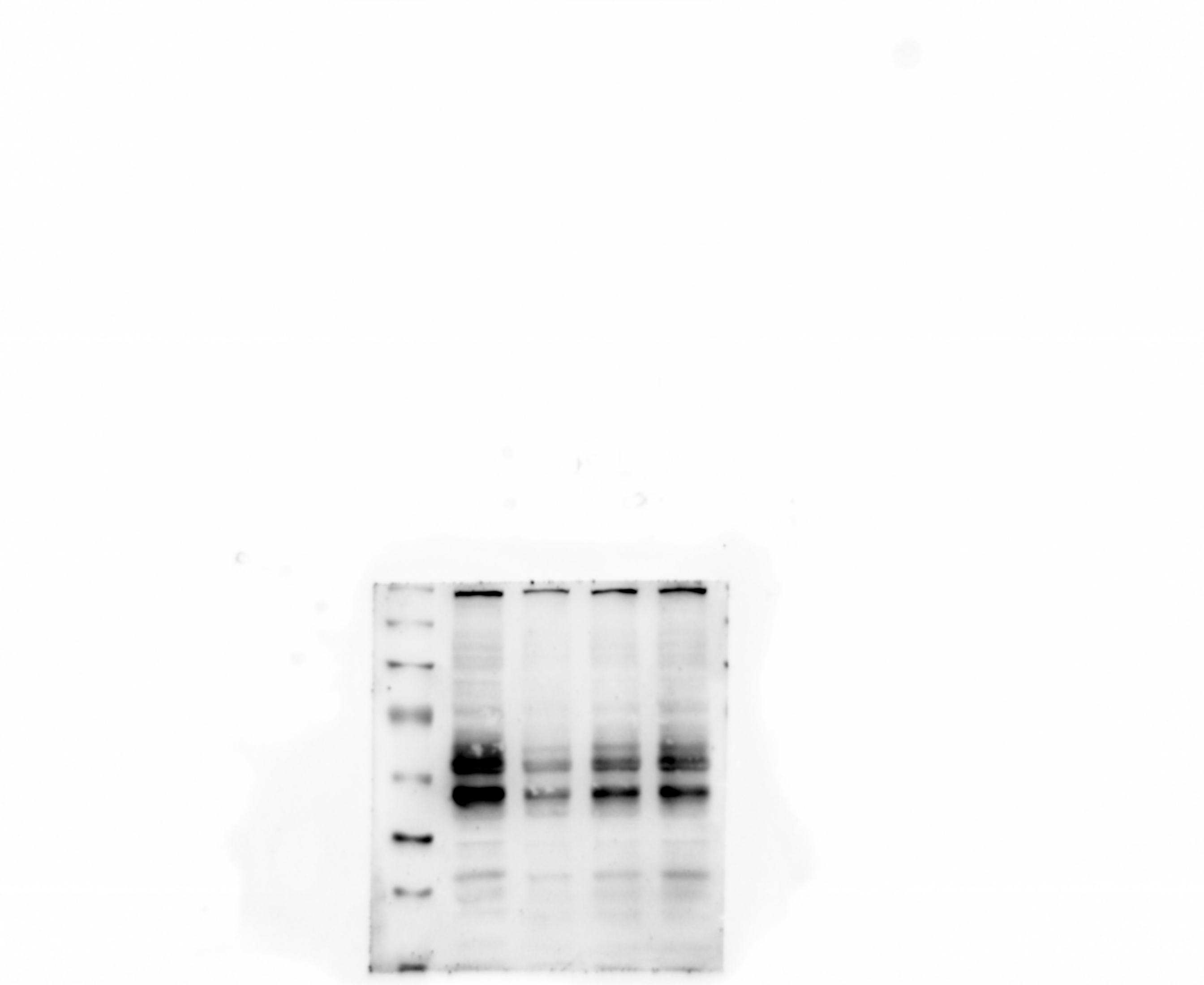

Supplement: Supplementary file 5 [file DataSheet2.zip › 003-luminescence[cmy1].tif]

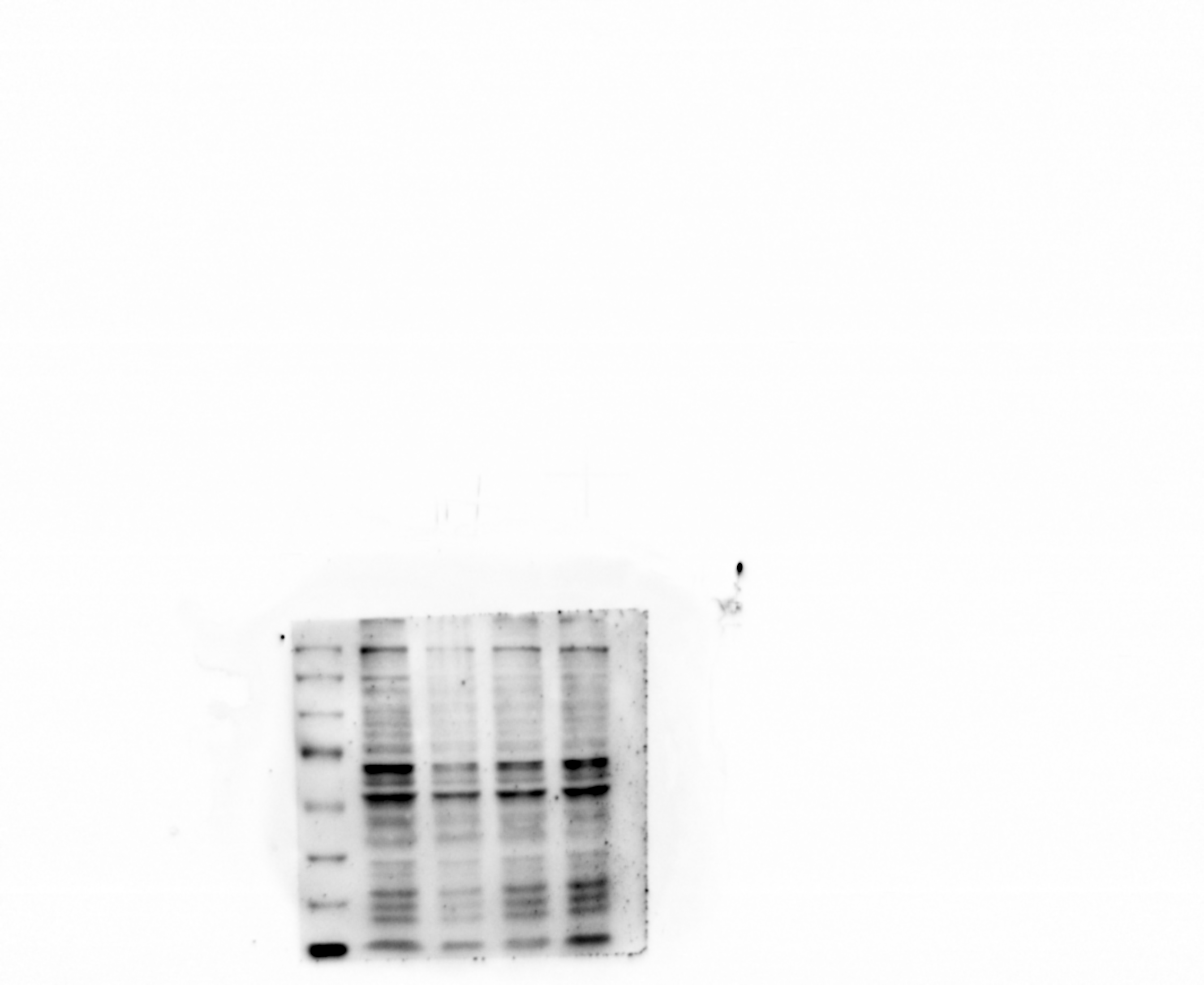

Supplement: Supplementary file 5 [file DataSheet2.zip › 003-luminescence[PS6-3].tif]

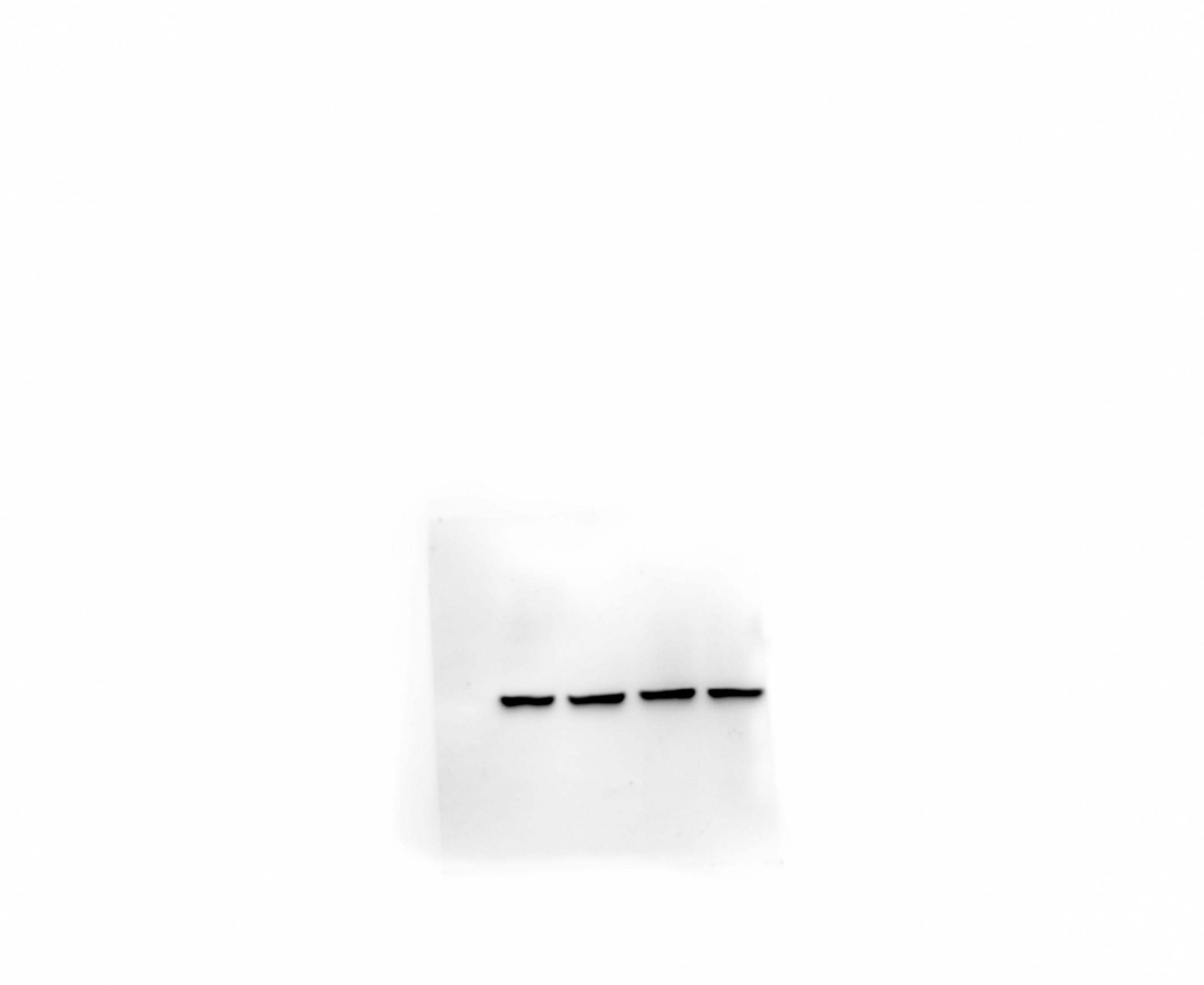

Supplement: Supplementary file 5 [file DataSheet2.zip › 009-luminescence[B2].tif]

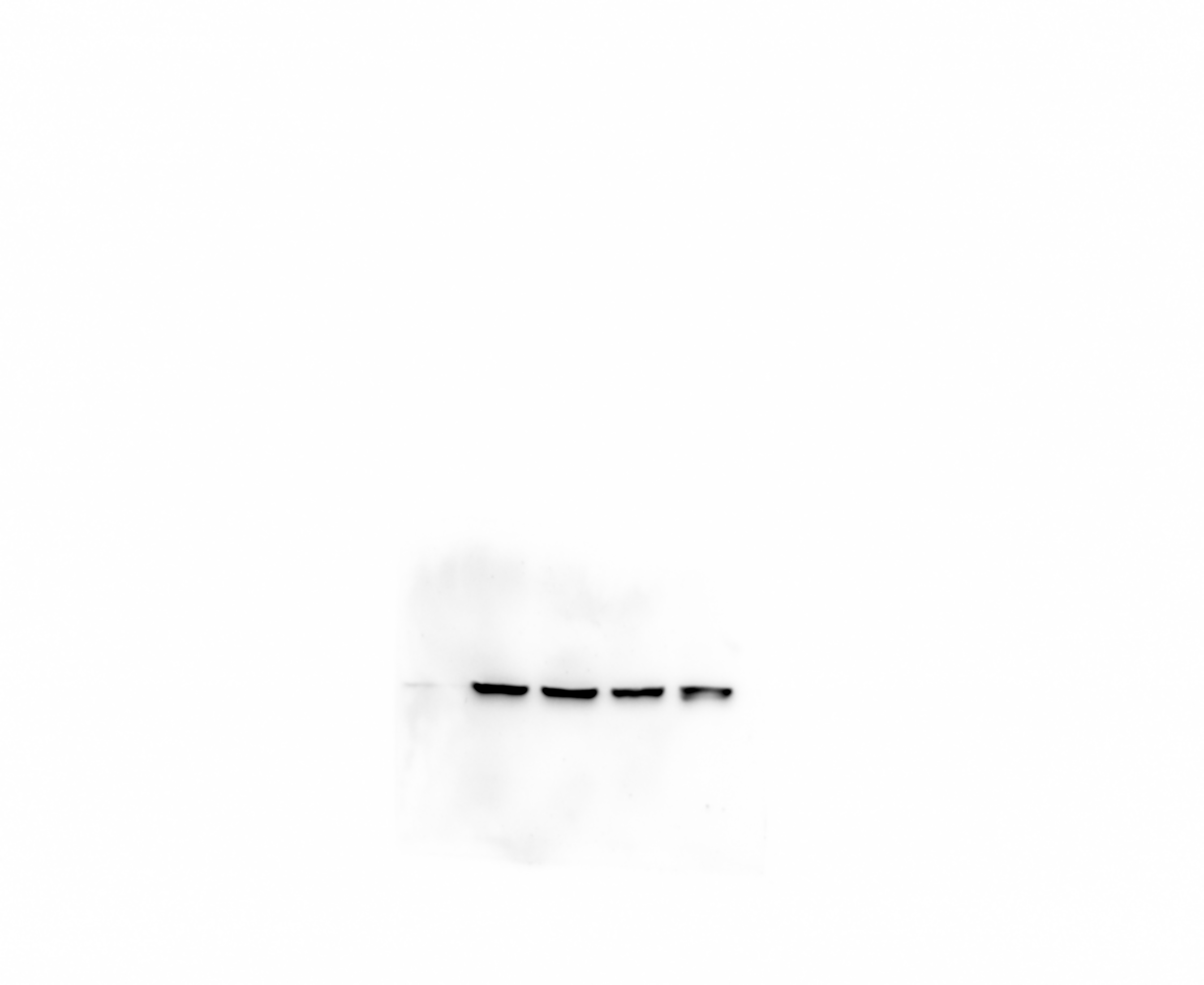

Supplement: Supplementary file 5 [file DataSheet2.zip › 010-luminescence[B1].tif]

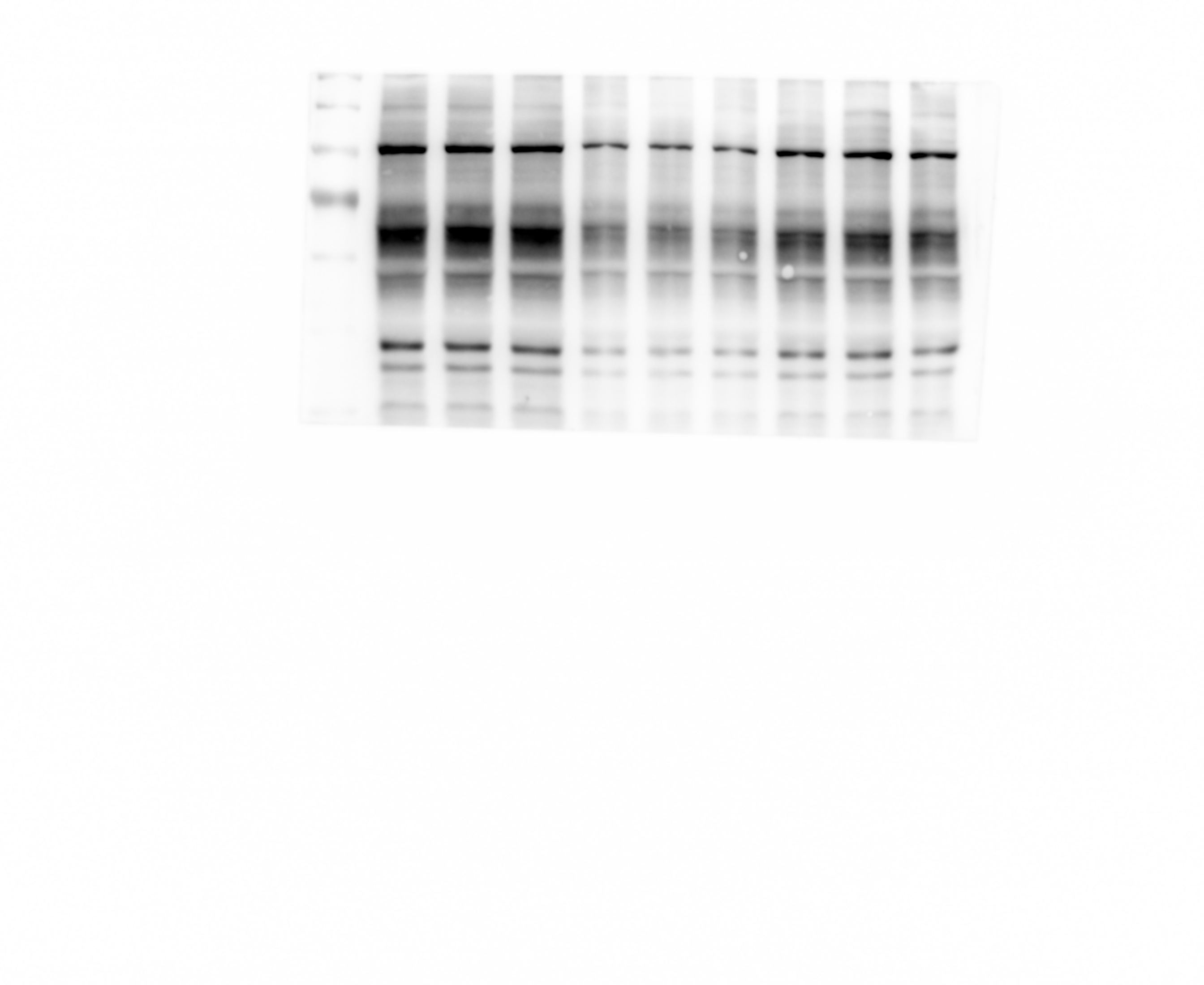

Supplement: Supplementary file 6 [file DataSheet5.zip › 003-luminescence[A].tif]

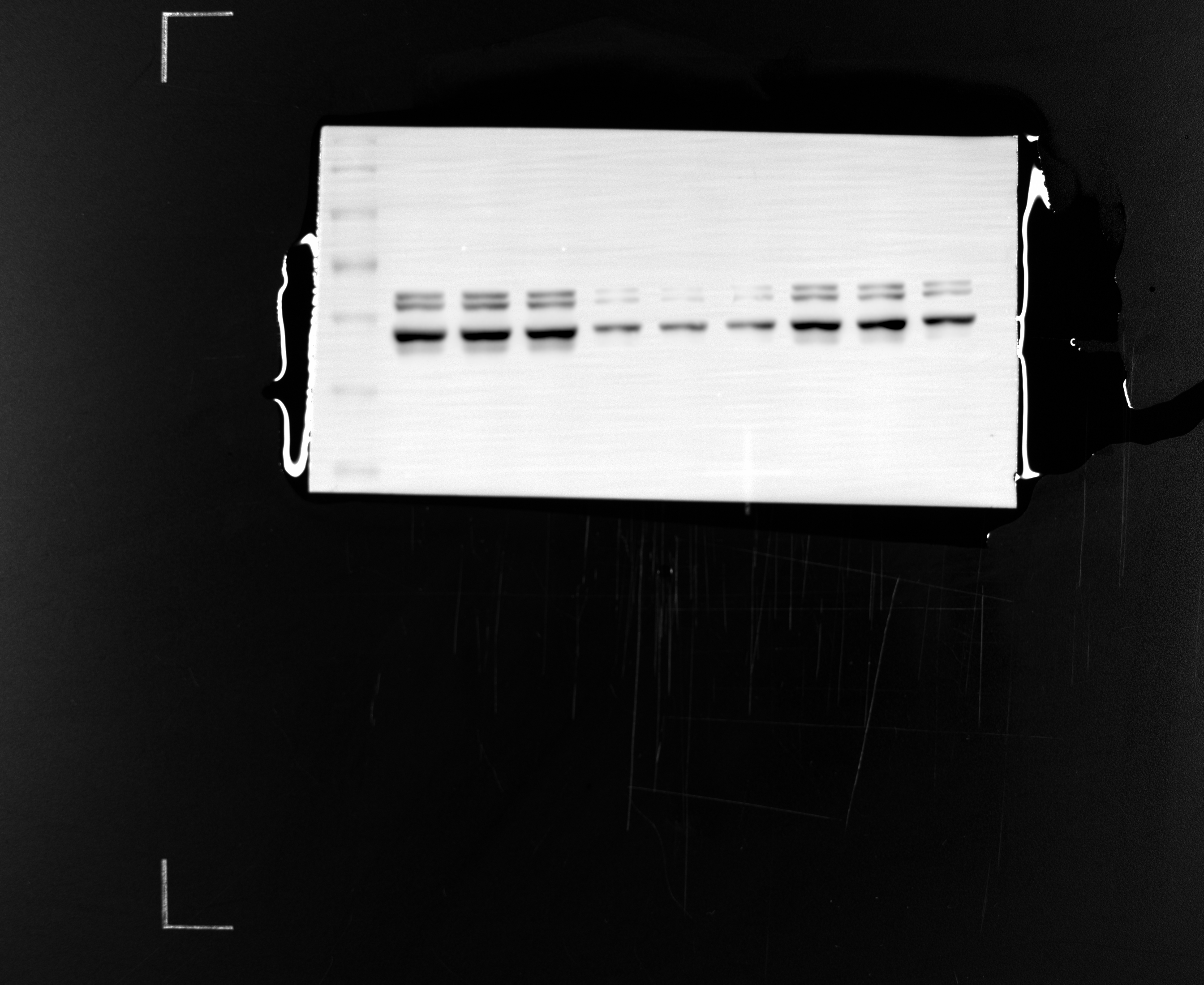

Supplement: Supplementary file 6 [file DataSheet5.zip › 011-overlay[G].tif]

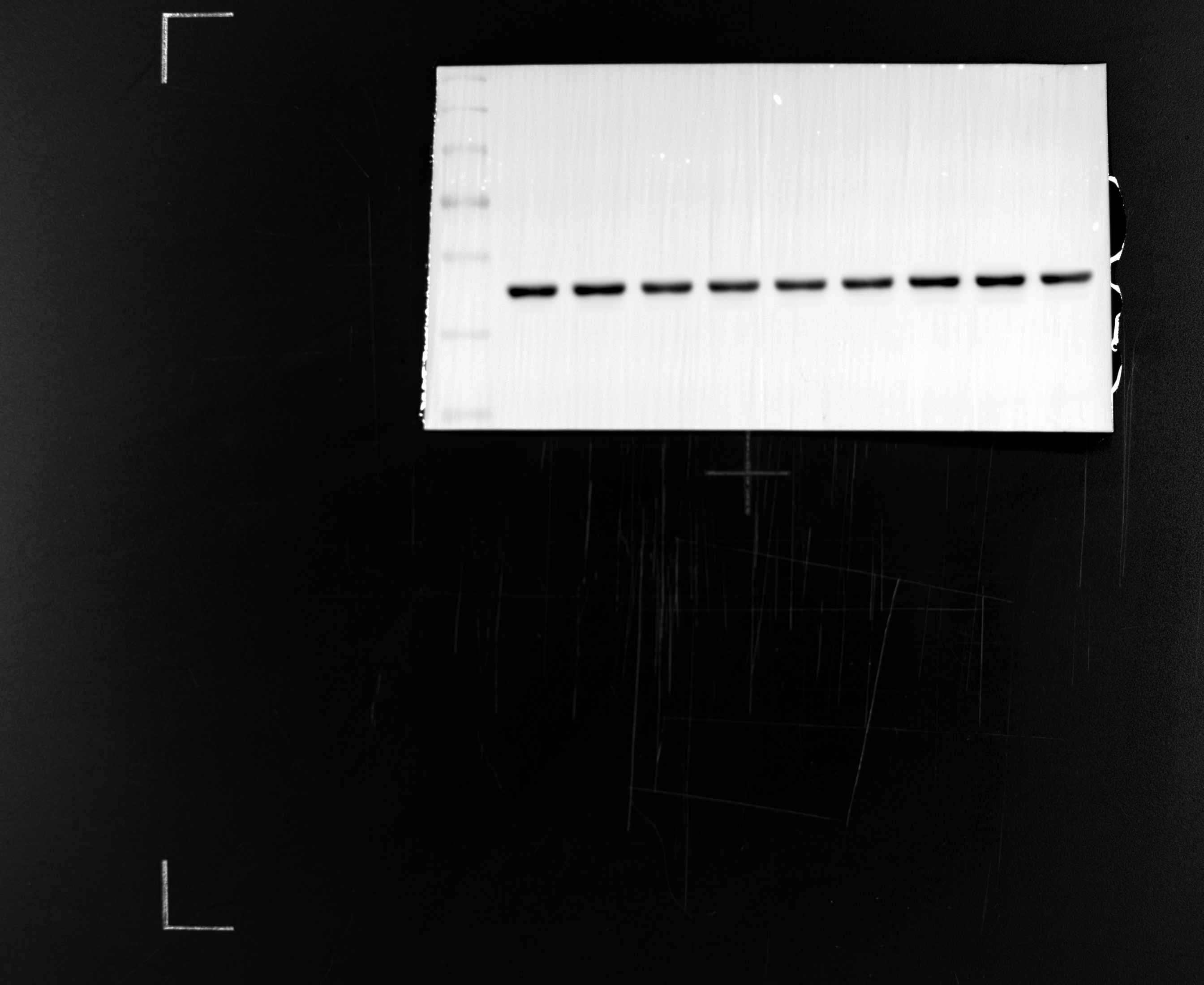

Supplement: Supplementary file 6 [file DataSheet5.zip › 013-overlay[btubulin].tif]

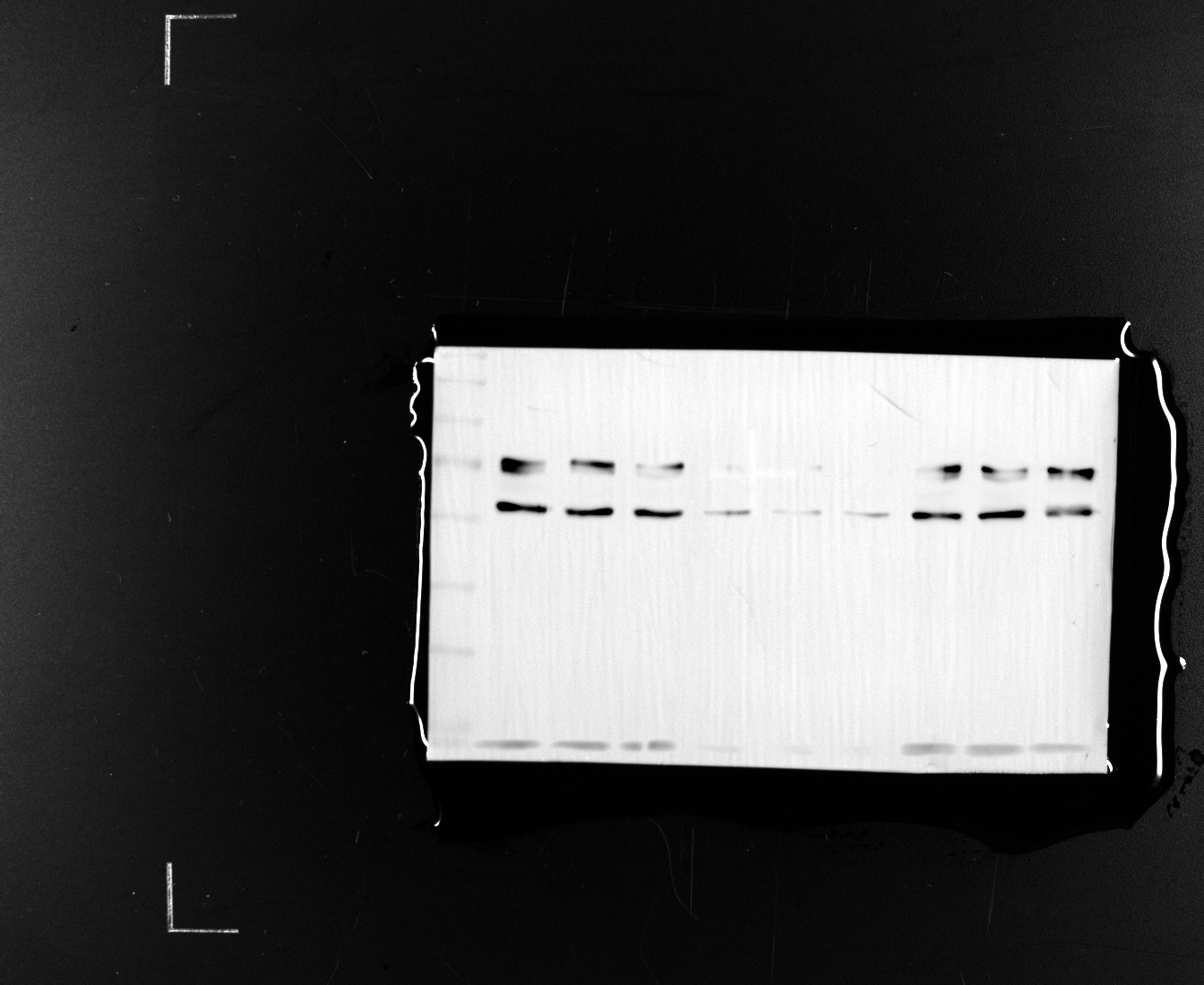

Supplement: Supplementary file 6 [file DataSheet5.zip › 179-merger [M].tif]
