# Supplementary material for: Sirtuin 6 mediates the therapeutic effect of endometrial regenerative cell-derived exosomes in alleviation of acute transplant rejection by weakening c-myc-dependent glutaminolysis
Source: Front Cell Dev Biol. 2025 Sep 18;13:1564382. doi: 10.3389/fcell.2025.1564382 (PMC12488686; doi:10.3389/fcell.2025.1564382)
Supplement: Supplementary file 2 [file Supplementaryfile1.docx]

**Supplementary Figures and Materials:**

**Supplementary Figures:**


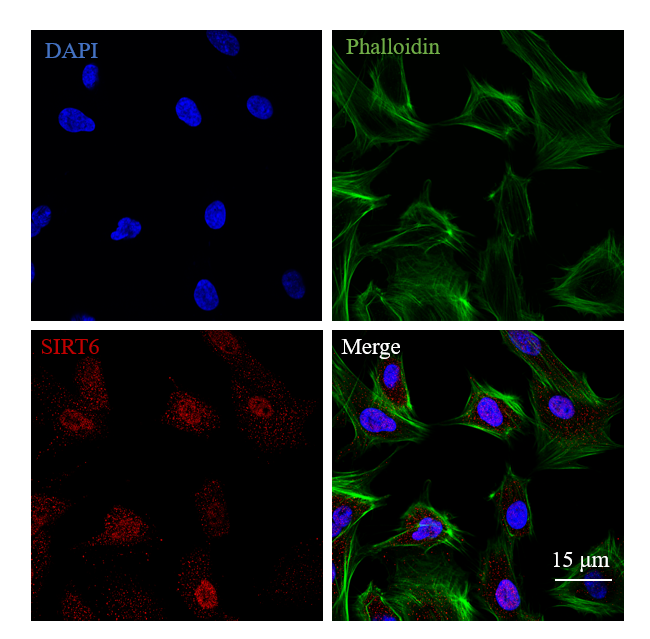


**Supplementary Figure S1: Subcellular localization of SIRT6 in ERCs.** Immunofluorescence staining showed the subcellular localization of SIRT6 in ERCs, in which blue represents DAPI stained nucleus, green represents phalloidin stained cells skeleton, and red represents SIRT6 stained SIRT6 protein (magnification 630×).


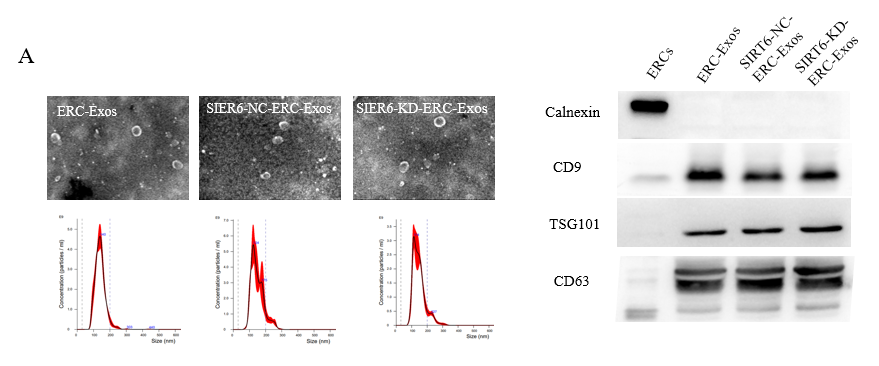


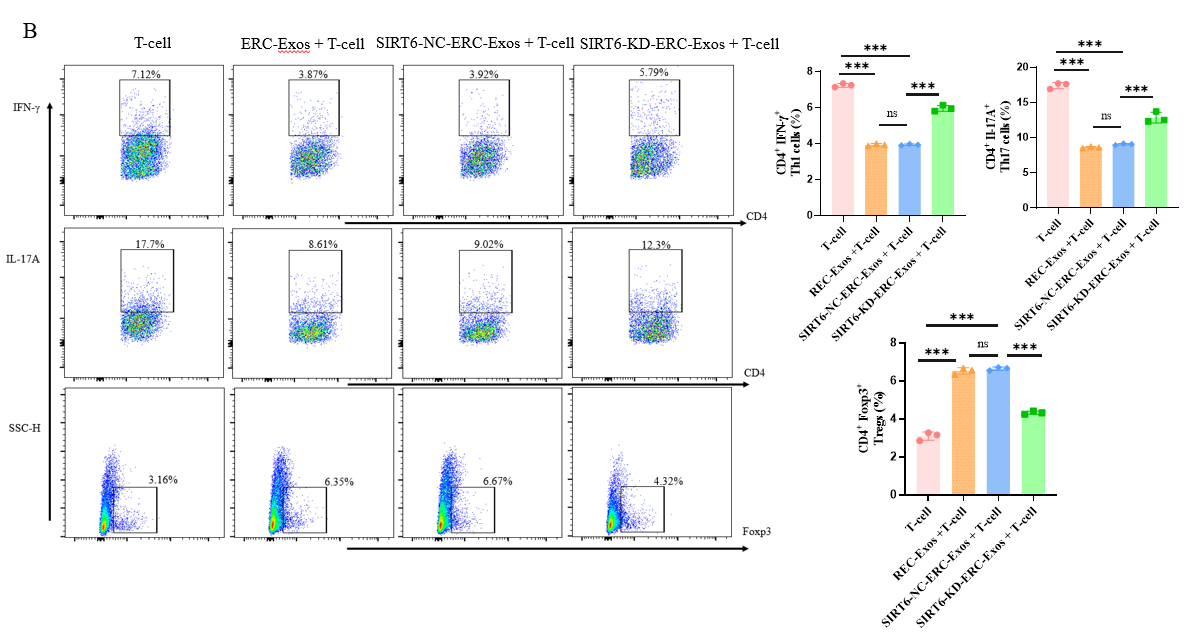


**Supplementary Figure S2: ERC-Exos and SIRT6-NC-ERC-Exos were identical in shape, size, and function.** There is no difference between ERC-Exos and SIRT6-NC-ERC-Exos through three landmark experiments (**A**) of exosome identification and CD4^+^ T cell differentiation experiments *in vitro* (**B**).


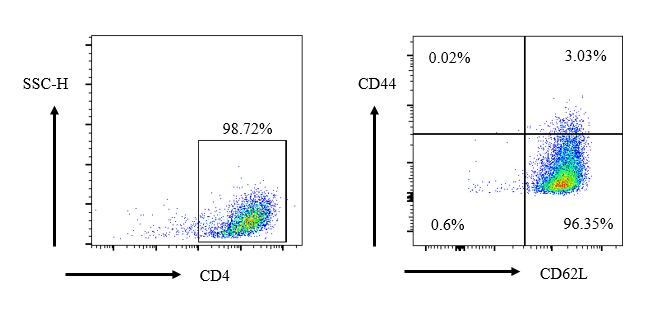


**Supplementary Figure S3: The purity of isolated naïve CD4^+^ T cells.** Naïve CD4+ T cells were immediately detected by flow cytometry after isolation, and the results showed that the purity of CD4^+^ T cells isolated from C57BL/c mice spleens was more than 98%. Besides, the recognized markers of naïve CD4^+^ T cells (CD44^lo^CD62L^hi^) in mice were also used for staining, and the results showed that the purity of naïve CD4+ T cells was over 96%. Because CD44CD62L can be used not only to identify naïve CD4^+^ T cells but also to identify effector T cells (CD44^hi^CD62L^lo^), the data show that effector T cells (0.02%) can be almost ignored in the newly extracted naïve CD4^+^ T cells of mice.


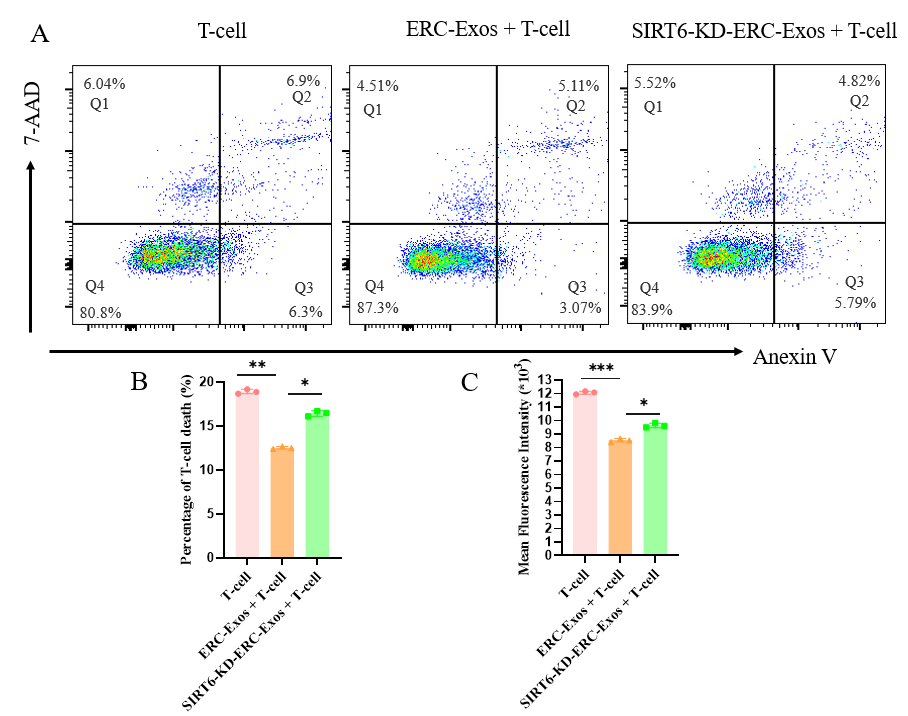


**Supplementary Figure S4:** **The cell death and proliferation during CD4^+^ T cell differentiation *in vitro*. A.** The representative pseudocolor plots of T cell death during CD4^+^ T cell differentiation *in vitro*. When ERC-Exo treatment was given, the proportion of dead cells in T cells decreased obviously, and when SIRT6 was knocked down, the decreasing trend increased again. **B.** The quantitative analysis of T cell death (Q1+Q2+Q3). **C.** The mean fluorescence intensity of Ki-67 of T cell proliferation during CD4^+^ T cell differentiation *in vitro*. ERC-Exo treatment obviously inhibited the excessive proliferation of T cells under inflammatory conditions, while the knock-down of SIRT6 in exosomes changed this trend.


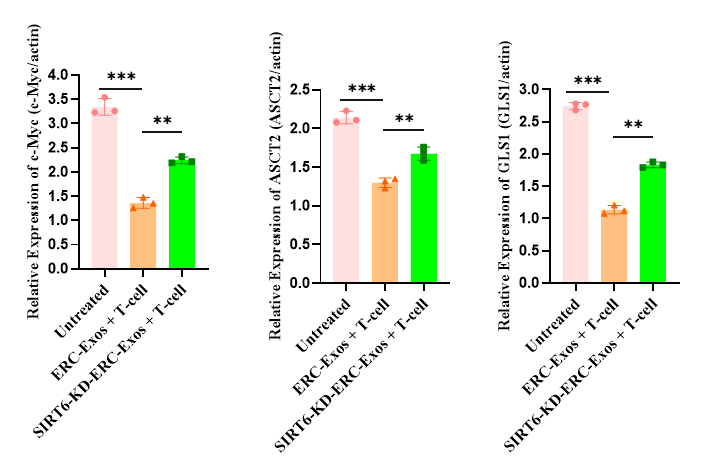


**Supplementary Figure S5: Quantitative real-time PCR detection of naïve CD4^+^ T cells.** After different treatments (untreated, ERC-Exo treated, SIRT6-KD-ERC-Exo treated) for 16-24 h, the mRNA expression level of c-Myc, ASCT2, and GLS1 were quantified via comparing with actin mRNA. The relative expression was normalized to actin by the 2^-ΔΔCt^ method.


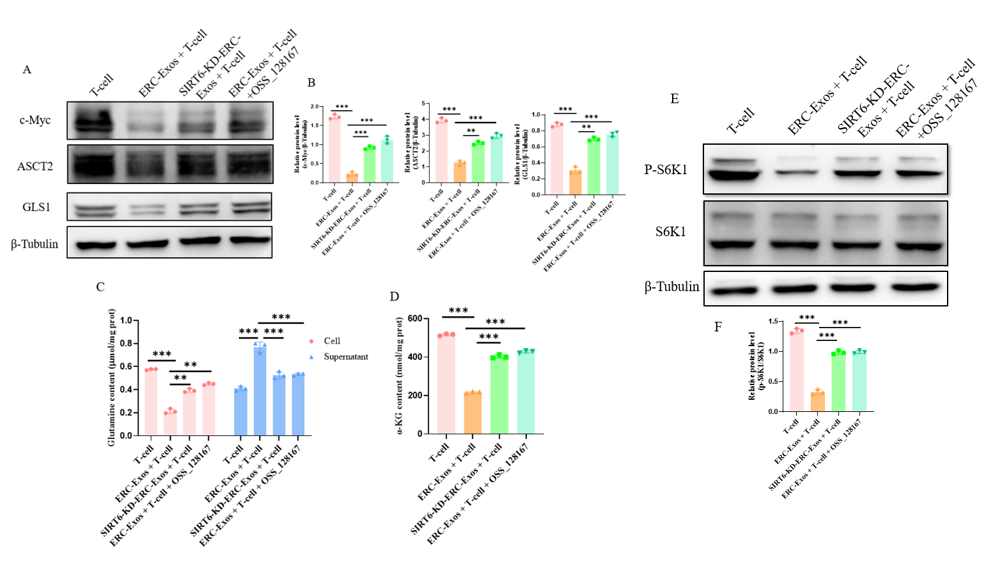


**Supplementary Figure S6: SIRT6 inhibitor OSS_128167 reverted the effect of ERC-Exos on regulating c-Myc/ glutaminolysis/ mTORC1 status.** When ERC-Exos was used to treat naïve CD4^+^ T cells, 100 μM OSS_128167 was added, and its effect on c-Myc-dependent glutaminolysis level of T cells and the activation state of mTORC1 were detected. **A.** The protein level change of c-Myc, ASCT2, and GLS1 after administration of 100 μM OSS_128167. **B.** The quantitative analysis of c-Myc, ASCT2, and GLS1 protein level. **C.** Detection of glutamine intake. **D.** Detection of intra-cellular α-KG content. **E.** The activation state of mTORC1 (represented by p-S6K1 level) after administration of 100 μM OSS_128167. **F.** The quantitative analysis of p-S6K1 protein level.


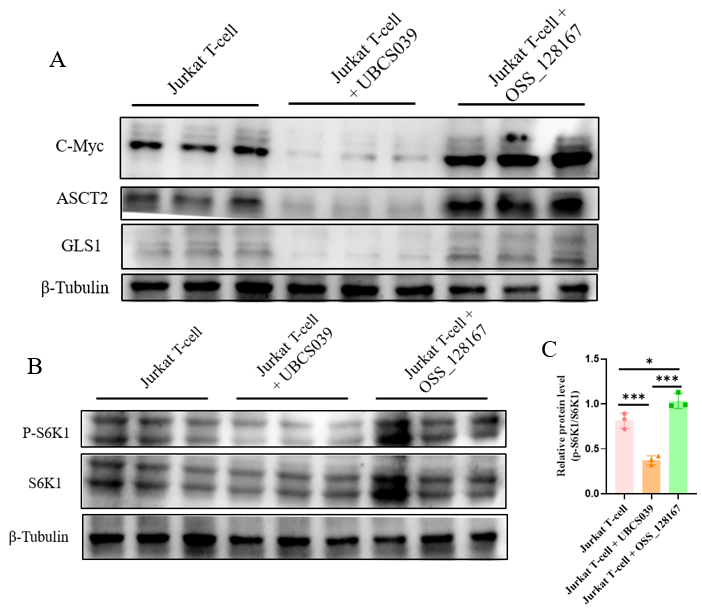

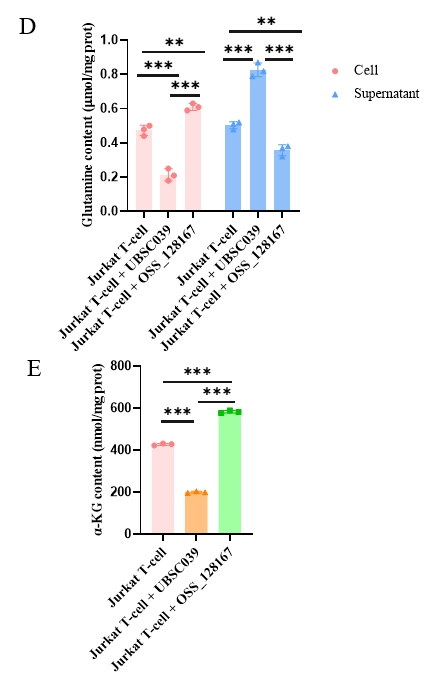


**Supplementary Figure S7: The effect of SIRT6 activation and inhibition on lymphocytes regarding glutaminolysis and c-Myc/mTORC1 status.** The function (glutaminolysis and cMyc/mTORC1 status) of SIRT6 in Jurkat cell was detected by using SIRT6 inhibitor (OSS_128167, 100 μM; MCE, Cat.No.: HY-107454) and activating agent (UBCS039, 38 μM; MCE, Cat.No.: HY-115453) after the activation of CD3/CD28 in Jurkat cell. **A.** The protein level change of c-Myc, ASCT2, and GLS1 after administration of OSS_128167 or UBCS039. **B.** The activation state of mTORC1 (represented by p-S6K1 level) after administration of OSS_128167 or UBCS039. **C.** The quantitative analysis of p-S6K1/S6K1 level. **D.** Detection of glutamine intake. **E.** Detection of intra-cellular α-KG content.


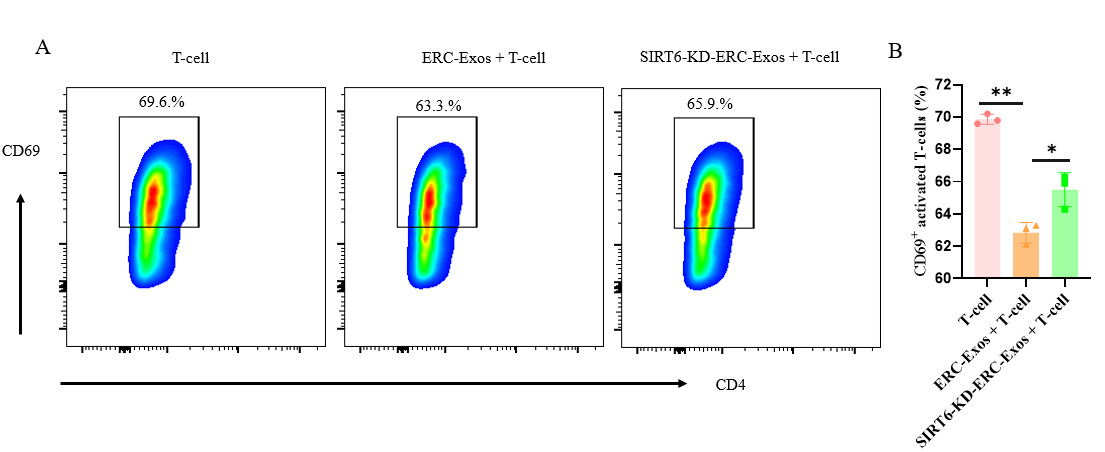


**Supplementary Figure S8: The activation level of naïve CD4^+^ T cells.** After different treatments (untreated, ERC-Exo treated, SIRT6-KD-ERC-Exo treated) for 12-24 h, the activation status of T cells was examined by flow cytometry. **A.** The level of T cell activation was detected by flow cytometry analysis of CD69, an early activation marker of T cells. **B.** Quantitative analysis of CD69 positive CD4^+^ T cells.

**Supplementary materials:**

**Immunofluorescence staining**

ERCs with a good growth state at passage 5 were adopted. The day before, the suspended ERCs were inoculated in a 12-well plate containing round coverslips, with a density of about 1×10^4^ cells/ml/well. When the cell aggregation reached 20-40%, the cells were taken out and washed twice with PBS, then 1 ml of 4% paraformaldehyde fixed solution was added to each well for cell fixation at room temperature for 20 min, then PBS was washed twice, and 0.1% Triton X-100 solution was added for cell permeability at room temperature for 15 min. Similarly, ERCs was washed twice with PBS, and 1% BSA blocking solution was added for nonspecific antigen blocking at room temperature for 1 h. Subsequently, the excess BSA blocking solution was sucked off, and SIRT6 antibody (dilution at 1:100, Abcam) was added for incubation at 4°C overnight. The next day, the incubated antibody was taken out and returned to room temperature for 30 min. After PBS washing twice, the corresponding cy3-labeled secondary antibody (dilution at 1:50, Proteintech) was incubated for 1 h at room temperature in the dark. After PBS washing twice, phalloidin (Yeason, Shanghai, China) working solution was added and incubated at room temperature for 1 h in the dark. After PBS washing twice, the round coverslip was taken out and dripped with neutral resin containing DAPI staining for sealing. The cells were observed under a confocal microscope.

**Annexin V/7-AAD flow staining**

Naïve CD4^+^ T cells were extracted from C57BL/6 mice and co-cultured with or without ERC-Exos or SIRT6-KD-ERC-Exos in 96-well plates to investigate whether SIRT6-expressing ERC-Exos can influence cell death during CD4^+^ T cell differentiation in vitro. In short, after 3 days of co-culture, T cell suspension was collected. Firstly, T cells were collected by centrifugation for 5 min at 300 *g* 4℃. Secondly, dilute 4× binding buffer (4 mL binding buffer + 12 mL deionized water) with 4 times deionized water to prepare 1× binding buffer. Subsequently, the cells were washed twice with PBS precooled at 4℃, each time with 300 *g*, and centrifuged at 4℃ for 5 min. 250 μL 1× binding buffer was added to resuspend the cells and to adjust their concentration to 1×10^6^ cells/mL. Then, 100 μL cell suspension was taken out and placed in a 5 mL flow tube, and 5 μL Annexin V-PE and 10 μL 7-AAD (Yeason, Shanghai, China) were added to each tube, which were gently mixed and reacted at room temperature in the dark for 15 min. Finally, 400 μL 1× binding buffer was added to each tube and mixed evenly, and the sample was detected within 1 h by flow cytometry. The percentage of different cells was analyzed using FlowJ and Graphpad software.

**Ki-67 flow staining**

Naïve CD4^+^ T cells were extracted from C57BL/6 mice and co-cultured with or without ERC-Exos or SIRT6-KD-ERC-Exos in 96-well plates to investigate whether SIRT6-expressing ERC-Exos can influence cell proliferation during CD4^+^ T cell differentiation in vitro. In short, after 3 days of co-culture, T cell suspension was collected. The collected cells were divided into 100 μL single cell suspensions and then stained with fluorescent-labeled antibodies, including zombie dye, anti-CD4-FITC, and anti-Ki67-APC, which were purchased from eBioscience (Thermofisher, USA) and BioLegend (Biolegend, USA). Finally, the mean fluorescence intensity (MFI) of different grouping T cells was analyzed using FlowJ software.

**SIRT6 inhibitor and** **active agent**

Sirt6 inhibitor (OSS_128167, 100 μM; MCE, Cat.No.: HY-107454)

Sirt6 active agent (UBCS039, 38 μM; MCE, Cat.No.: HY-115453)

SIRT6 inhibitors and activators involved in T cell experiments are all added to the culture system together with ERC-Exos to treat naïve CD4^+^ T cells or Jurkat cell line for 24 h.

**Quantitative RT-PCR**

Total RNA of naïve CD4^+^ T cell after different treatments for 16-24 h was isolated from the indicated cells using RNA extraction kit (Genstar, Beijing) and subjected to cDNA synthesis using cDNA reverse transcription kit (Genstar, Beijing). Then quantitative real-time PCR detection was carried out by commercial kit (TransGen, Beijing). Gene-specific primer sets (all for murine genes) used in real-time PCR assays were: c-Myc, 5’-GCCTCAGAGTGCATCGAC-3’ and 5’-TCCACAGAAACAACATCG-3’, ASCT2, 5’-GAAGACTCAAACAACAGAGGG-3’, and 5’-TCCTCTGTGGCGAGGGGCAG-3’, GLS1, 5’-TCTACAGGATTGCGAACGTCT-3’ and 5’-CTTTGTCTAGCATGACACCATCT-3’, ACTIN, 5’-TCTGGCACCACACCTTCTAC-3’ and 5’-GATAGCACAGCCTGGATAGC-3’. The relative expression was normalized to actin by the 2^-ΔΔCt^ method.

**Supplementary Raw Data Files contents:**

Data Sheets 1: This folder corresponds to the supplementary figures. Where "G" corresponds to the triplicate experiment of GLS1 protein. "A" represents three replications of ASCT2 protein. "Tub" stands for β-tubulin protein. "MYC" stands for c-Myc protein. "PSSS" stands for p-S6K1 protein. Due to the limited file size, the results of the other two repeated experiments of β-Tubulin, c-Myc and p-S6K1 are in other folders.

Data Sheets 2: This folder corresponds to the supplementary figures. Where "B" corresponds to the other two repeated experiments of β-tubulin protein, which matches folder 1. "cmy" corresponds to the other two repeated experiments of c-Myc protein, which matches folder 1. "PS6" stands for p-S6K1 protein.

Data Sheets 3: This folder corresponds to figure 2 in the manuscript. Among them, "SIRT6" and "beta-SIRT6" are a corresponding set, representing SIRT6 and β-tubulin, respectively. "001-merger" and "002-merger" are a corresponding set, representing exosome proteins SIRT6 and CD9, respectively. "0", "00", "000" and "0000" are corresponding sets, representing exosome marker proteins TSG101, CD63, CD9 and calnexin, respectively.

Data Sheets 4: This folder corresponds to figure 7 in the manuscript. "B" and "b" represent three replications of β-tubulin protein. "S6K" represents three replications of S6K1 protein. "pS6" represents three replications of p-S6K1 protein. "S T" represents three replications of SIRT6 protein in T-cells.

Data Sheets 5: This folder corresponds to figure 7 in the manuscript. "M" represents three replications of c-Myc protein. "G" represents three replications of GLS1 protein. "A" represents three replications of ASCT2 protein. "btubulin" represents three replications of β-tubulin protein.
